# All-soft organic electrochemical diodes based on asymmetric active layers for signal isolation in deformable electronics

**DOI:** 10.1126/sciadv.aei3178

**Published:** 2026-10-21

**Authors:** Sehyun Kim, Minji Kim, Haechan Park, Kihyun Sim, Jihyeon Jang, Yerin Kim, Jinyeong Park, Donghyung Shin, Myeonghyeon Na, Kyoseung Sim

**Affiliations:** ^1^ Department of Chemistry, Ulsan National Institute of Science and Technology (UNIST), Ulsan, 44919, Republic of Korea.; ^2^ X-Dynamic Research Center, Ulsan National Institute of Science and Technology (UNIST), Ulsan, 44919, Republic of Korea.

## Abstract

All-soft electronics have emerged as a promising platform for deformable and human-centric systems, yet the development of mechanically compliant diodes remains a major challenge due to limitations in material compatibility, device architecture, and stable operation under deformation. Electrochemical approaches offer a viable route to address this challenge by enabling bulk, low-voltage charge modulation in soft materials without relying on rigid junction interfaces. Here, we report, to the best of our knowledge, the first all-soft organic electrochemical diode (OECD) based on an asymmetric active-layer geometry. The device incorporates a transfer-printed ultrathin ion-gel interface that preserves conformal coverage and the active electrochemical architecture under strain. The device achieves high rectification through polarity-dependent electrochemical potential redistribution, with a rectification ratio of up to 2.23 × 10^4^ at ±1 V and stable operation under tensile strains of up to 30% and repeated mechanical deformation. This ion-gel-based architecture preserves the electrochemical asymmetry required for diode operation while maintaining structural integrity under strain. Beyond the individual device, the all-soft OECD enables deformable rectifiers and logic gates and functions as a key signal-isolation element that suppresses cross-talk in a deformable pressure-sensing array. These results establish an electrochemical diode platform that combines mechanical deformability, high rectification performance, and circuit-level compatibility, providing a pathway toward fully deformable and integrated human-centric electronic systems.

## INTRODUCTION

All-soft electronics, composed entirely of mechanically soft materials, represent an ideal model of deformable electronic systems for emerging human-centric applications, including electronic skin (*1*–*4*), wearable healthcare systems (*5*–*8*), implantable devices (*9*–*12*), and human-machine interfaces (*13*–*16*). Although recent efforts on all-soft transistors, sensors, and energy devices have highlighted their strong potential (*17*–*22*), the development of all-soft diodes, which are indispensable for rectification, protection, and signal processing, remains relatively limited compared with other device classes. Previous efforts to develop all-soft diodes using polymeric semiconductors (*23*–*25*) have relied on complex multilayer architectures and demanding fabrication processes, and their purely electronic operation was incompatible with ion-rich environments, collectively limiting their scalability and applicability in emerging electronic systems. Ionic diodes, as another attempt, rectify ionic currents and provide valuable functions in iontronics and biofluidic systems (*26*–*28*), but their reliance on liquid electrolytes, slow ionic mobility, and limited integrability make them unsuitable for conventional electronic circuits. These limitations highlight the urgent need for a new diode platform that is inherently compatible with soft electronics.

A new paradigm for soft diodes lies in exploiting electrochemical doping, following the well-established principles of organic electrochemical transistors (OECTs), which involve simple, solution-processable device architectures with inherent compatibility to ion-rich environments (*29*–*31*). This approach not only overcomes the limitations of purely electronic or ionic diodes but also offers a critical advantage through process and material compatibility with OECTs, a leading technology in soft and bio-integrated electronics. Indeed, Yoon and co-workers recently demonstrated, for the first time, an organic electrochemical diode (OECD) by employing asymmetric electrochemical doping and dedoping induced by an unbalanced spatial active layer design, thereby achieving high performance with a simple planar structure and a facile fabrication process (*29*). This landmark study highlighted the strong potential of OECDs as indispensable components in the field of soft electronics. However, this approach still relies on rigid substrates and electrodes, leading to mechanical mismatch with the human body, and its dependence on liquid electrolytes such as NaCl solution involves further challenges in scalability, encapsulation, and long-term stability, which are particularly critical for translation into emerging soft electronic systems. Therefore, achieving all-soft OECDs through innovations in material design, device architecture, and fabrication strategy, a critical challenge that has remained unresolved, marks a key step toward advancement of soft electronics.

Here, we report, to the best of our knowledge, the first all-soft OECD based on an asymmetric active-layer geometry, featuring high rectification performance at low operating voltages. The device overcomes the intrinsic rigidity of the previously reported concept and establishes a fully deformable platform by integrating entirely soft components with a conformal ultrathin ion-gel interface formed via transfer printing. Importantly, this architecture preserves the active-layer asymmetry under tensile strain, allowing polarity-dependent electrochemical potential redistribution and the associated doping and dedoping processes to remain intact during deformation. Beyond individual device performance, the OECD is readily extended to circuit-level implementations, including deformable rectifiers and logic gates, highlighting its versatility as a functional building block. Furthermore, integration with soft sensing components enables cross-talk-free operation in a deformable pressure-sensing array, demonstrating its key role as a signal-isolation element at the system level. These results establish the all-soft OECD as a mechanically robust and system-compatible diode platform, providing a viable pathway toward fully deformable and human-centric electronic systems.

## RESULTS

### All-soft OECDs

Figure 1A shows an optical image of a fabricated 5 × 5 all-soft OECD array under tensile strain, composed entirely of mechanically soft components, including polydimethylsiloxane (PDMS) as the soft substrate, gold nanoparticle-coated silver nanowires embedded in PDMS (AuNPs-AgNWs/PDMS) as the soft electrodes, poly(3-hexylthiophene) nanofibrils/PDMS (P3HT-NFs/PDMS) as the soft semiconducting layer, and an ion-gel as the soft electrolyte, as described in Materials and Methods. The operation of the all-soft OECD is based on polarity-dependent electrochemical potential redistribution induced by an asymmetric active-layer geometry, which leads to unbalanced ionic doping and dedoping of the P3HT-NFs/PDMS layer (*29*). As shown in Fig. 1B, the asymmetric geometry leads to unequal volumetric capacitances at the anode and cathode, resulting in a larger local electrochemical potential shift at the smaller-area electrode under an applied voltage. Under forward bias (*V*_Bias_ > 0 V), this amplified potential shift exceeds the oxidation onset potential (*E*^0^), enabling anion injection into the anode-side P3HT-NFs/PDMS and inducing electrochemical hole doping accompanied by a downward shift of the electronic energy levels. In contrast, under reverse bias (*V*_Bias_ < 0 V), the local electrochemical potential remains below *E*^0^, suppressing anion injection and stabilizing a dedoped, non-conductive state with an upward shift of the energy levels. This area-dependent electrochemical potential redistribution under opposite bias polarities gives rise to current rectification, following the operating principle previously reported for asymmetric OECDs (*29*).

**Fig. 1.**
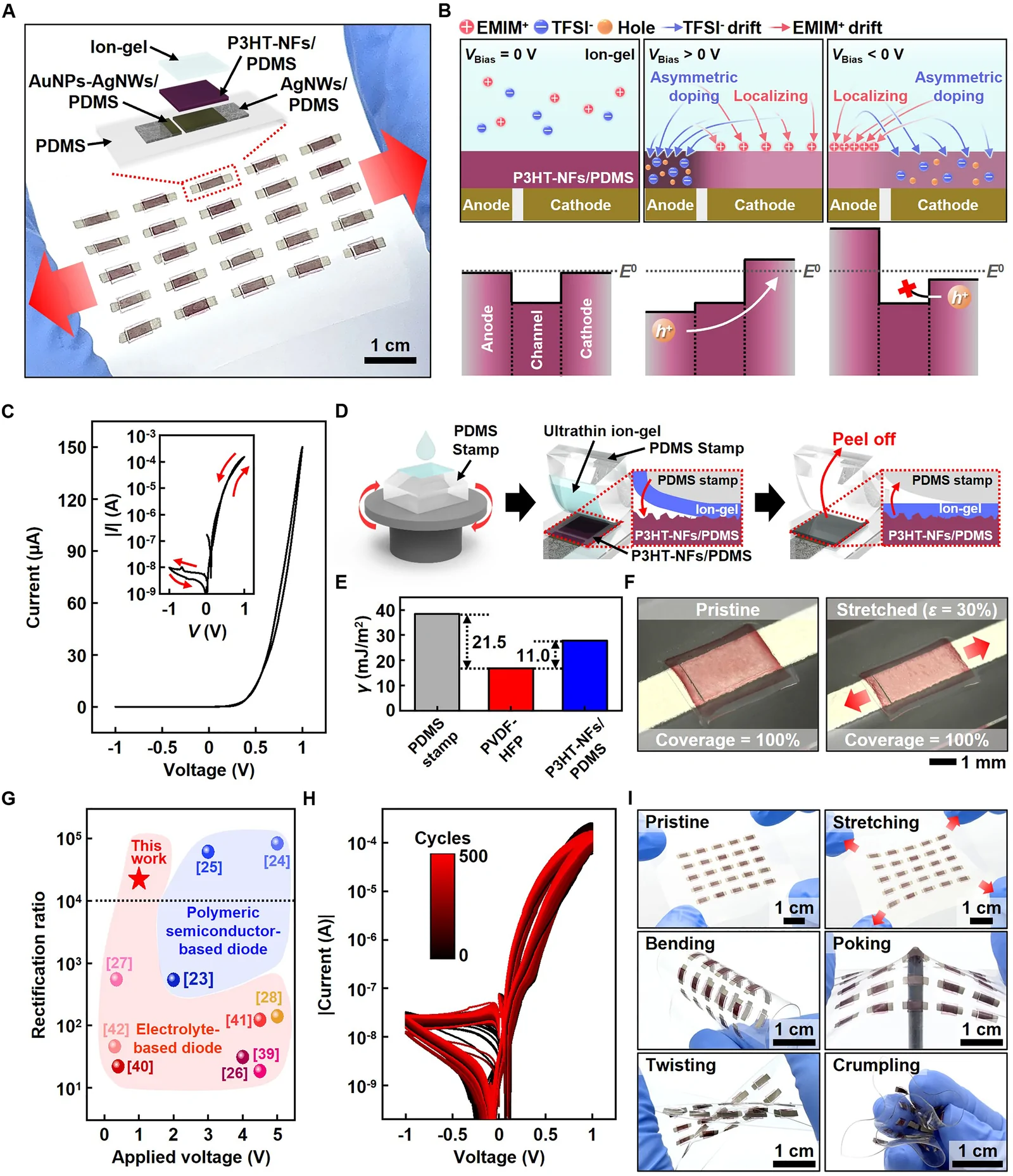
All-soft OECD enabled by asymmetric active-layer geometry. (**A**) Optical image of a 5 × 5 all-soft OECD array. Inset shows an exploded schematic illustration of a single all-soft OECD. (**B**) Schematic illustrations of the ionic distributions and corresponding local energy-level shifts under zero (left), positive (middle) and negative (right) bias, showing asymmetric electrochemical doping in the P3HT-NFs/PDMS layer. (**C**) Current-voltage (*I-V*) characteristics of the all-soft OECD. Inset shows the same data on a semi-logarithmic scale. (**D**) Schematic illustration of the transfer-printing process used to integrate the ultrathin ion-gel onto the P3HT-NFs/PDMS layer. (**E**) Obtained surface energy of PDMS stamp, PVDF-HFP, and P3HT-NFs/PDMS. (**F**) Optical images of the transferred ultrathin ion-gel on the P3HT-NFs/PDMS layer in the pristine state and under 30% tensile strain. (**G**) Comparison of the rectification ratio and operating voltage of the all-soft OECD with those of previously reported diodes for deformable electronics. (**H**) *I-V* characteristics of the all-soft OECD measured over 500 consecutive cycles. (**I**) Optical images of the all-soft OECD array under various mechanical deformations, including stretching, bending, poking, twisting, and crumpling.

The rectification mechanism relies on ion-selective electrochemical doping of P3HT, in which only anion injection contributes to charge modulation, as confirmed by electrochemical impedance spectroscopy (EIS) (fig. S1). Accordingly, a device with a degree of asymmetry (DoA) of 90% exhibits clear diode characteristics (Fig. 1C and fig. S2), consistent with those of previously reported rigid OECDs based on the same working mechanism (*29*). In contrast, control devices without the ion-gel or without the P3HT-NFs/PDMS layer show no rectifying behavior, confirming that chemical doping is indispensable for diode operation (figs. S3 and S4). Because the electrochemical modulation of the all-soft OECD occurs throughout the P3HT-NFs/PDMS active layer, the active-layer thickness was optimized by varying the P3HT-NFs solution concentration. The optimized condition provided balanced diode performance in terms of forward current (*I*_F_), rectification ratio (RR), hysteresis, and operational stability during repeated cycling and was adopted for all subsequent devices (fig. S5).

In addition to active-layer asymmetry, ion-gel configuration also determines diode performance and mechanical robustness. Symmetric ion-gel distributions that expose large electrode areas (anode side) increase the reverse current (*I*_R_) due to excessive interfacial charge accumulation at electrode-electrolyte contacts, which redistributes the local electrochemical potential and prevents the formation of a stable dedoped state under reverse bias (fig. S6). These results indicate that maintaining ion-gel coverage over the semiconductor is essential for preserving stable rectification during mechanical deformation. When interfacial adhesion between the ion-gel and the semiconductor is insufficient to fully accommodate the mechanical mismatch with the substrate, strain transfer within the device stack becomes strongly thickness dependent (*32*–*34*). Under uniaxial stretching, deformation of the ion-gel layer is governed primarily by its axial stiffness and interfacial shear strength (*34*, *35*). As schematically illustrated in fig. S7, thick ion-gels, with relatively high axial stiffness, undergo internal strain dissipation and interfacial slippage, leading to partial decoupling from the underlying layers. In contrast, ultrathin ion-gels exhibit substantially reduced axial stiffness, enabling efficient strain transfer through interfacial shear and mechanically constraining the ion-gel to deform conformally with the device stack while maintaining uniform coverage on the P3HT-NFs/PDMS layer.

Because the handling of ultrathin ion-gels is not well suited to conventional cut-and-stick approaches widely used in soft electronics (*36*–*38*), we integrated the ultrathin ion-gel via a transfer-printing process, as shown in Fig. 1D. The successful transfer is enabled by favorable wetting of the ionic liquid on the P3HT polymeric network (fig. S8) and closer surface-energy matching between PVDF-HFP and P3HT compared with that between PVDF-HFP and the PDMS stamp (Fig. 1E, fig. S9, note S1 and table S1). As a result, the transferred ultrathin ion-gel deforms synchronously with the device under applied strain (Fig. 1F). This optimized ion-gel with an average thickness of 62.7 μm, determined from systematic deformation and coverage studies, was used for all subsequent devices (fig. S10). In addition, comparable rectification across different ion-gel thicknesses indicates sufficient ion supply for electrochemical doping during OECD operation (fig. S11).

The complete fabrication process for the all-soft OECD is schematically illustrated in fig. S12 and described in detail in the Materials and Methods. AgNWs/PDMS electrodes were selected as the base soft conductor because the percolated AgNW network provides high electrical conductivity while maintaining mechanical compliance within the elastomeric PDMS matrix. The AuNP coating on the exposed AgNWs within the AgNWs/PDMS electrodes reduces the contact resistance at the electrode-semiconductor interface (*37*, *38*), allowing the applied potential to be delivered more efficiently to the active layer during operation. This reduced interfacial potential loss enhances the *I*_F_ while suppressing the reverse current (fig. S13), leading to a high RR at low applied voltages compared with previously reported diodes (Fig. 1G and table S2) (*23*–*28*, *39*–*42*). In addition, the device maintains reasonably stable rectification behavior over 500 repeated measurement cycles, with the slight RR decreasing from 7.81 × 10^3^ to 4.19 × 10^3^ (Fig. 1H and fig. S14). The device also retained rectifying characteristics after 21 days of ambient storage without substantial degradation, further supporting its practical stability (fig. S15). Owing to its entirely soft material composition, the device maintains structural integrity under mechanical deformation without observable physical damage (Fig. 1I).

### Characterization of all-soft OECDs

The fabricated all-soft OECD array exhibits uniform device configuration and electrical characteristics across the substrate in the undeformed state, as shown in Fig. 2 (A and B). The array shows an average rectification ratio (RR_AVE_) of 8.31 × 10^3^ at an applied voltage of ±1 V, with a maximum value of 2.23 × 10^4^, and an average forward current (*I*_F, AVE_) of 225 μA and a maximum value of 411 μA. These results demonstrate that the rectifying performance is consistently reproduced across the array, confirming excellent device uniformity and functional reproducibility (Fig. 2C and fig. S16). The high rectification performance originates from the asymmetric electrochemical architecture, which maximizes bulk electrochemical conduction under forward bias while strongly suppressing current under reverse bias through polarity-dependent electrochemical potential redistribution. This mechanism is fundamentally distinct from interfacial barrier-controlled or contact-limited transport commonly exploited in previously reported soft diodes (*24*, *27*).

**Fig. 2.**
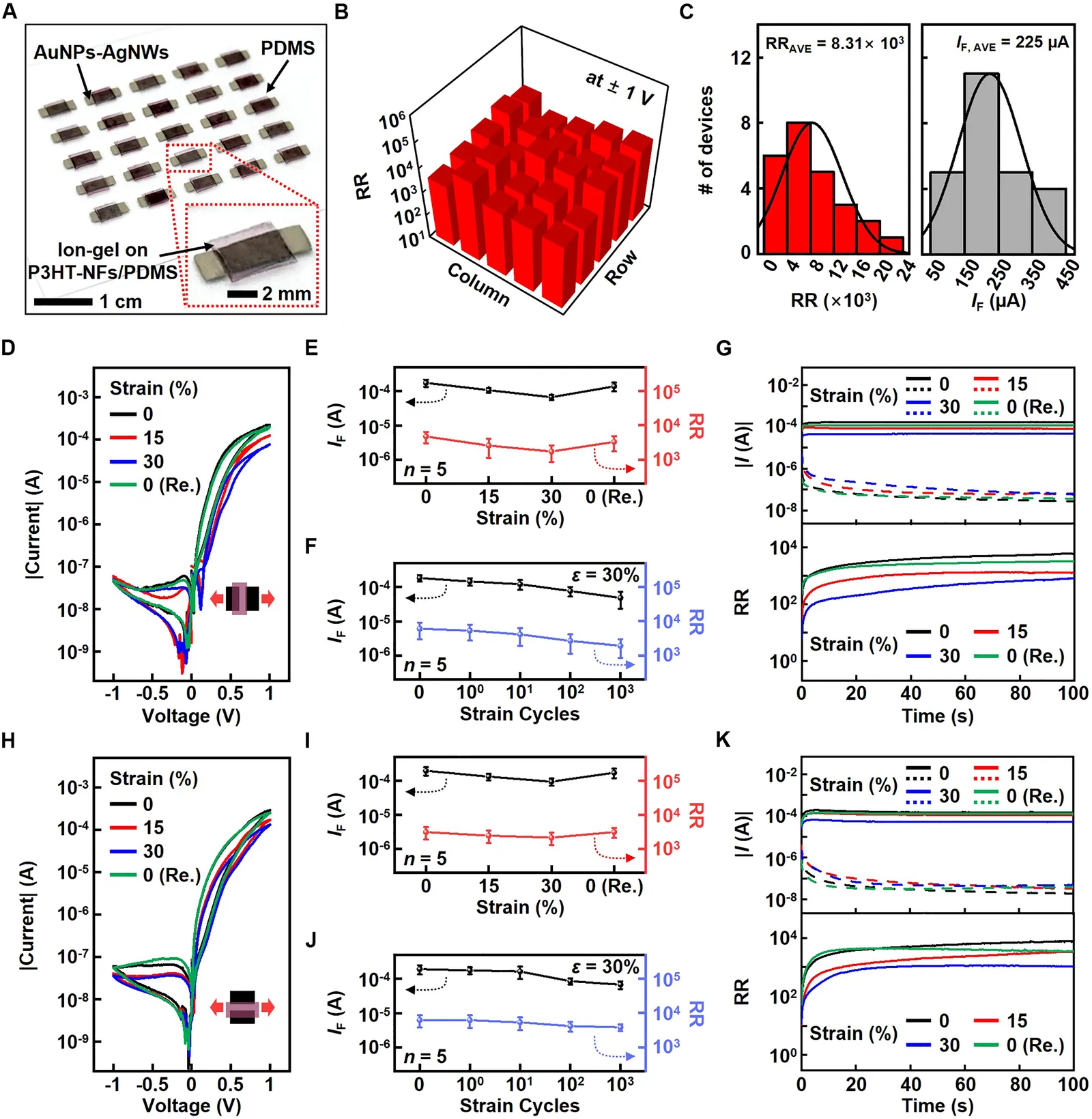
Electrical performance and strain tolerance of the all-soft OECD. (**A**) Optical image of a 5 × 5 all-soft OECD array. Inset shows a magnified optical image of a single all-soft OECD. (**B**) Rectification ratio (RR) map of the 5 × 5 all-soft OECD array measured at ±1 V. (**C**) Statistical distributions of RR and forward current (*I*_F_) for the all-soft OECD array. (**D**) Representative *I-V* characteristics of the all-soft OECD under tensile strains of 0, 15, and 30%, and after release, with strain applied parallel to the channel length direction. (**E** and **F**) Extracted *I*_F_ and RR of the all-soft OECD as functions of tensile strain (E) and the number of repeated strain cycles at 30% tensile strain (F) applied parallel to the channel length direction. (**G**) Current responses measured at constant biases of +1 V (solid lines) and −1 V (dashed lines) (top), and the corresponding RR values (bottom), under tensile strains of 0, 15, and 30%, and after release, with strain parallel to the channel length direction. (**H**) Representative *I-V* characteristics of the all-soft OECD under tensile strains of 0, 15, and 30%, and after release, with strain applied perpendicular to the channel length direction. (**I** and **J**) Extracted *I*_F_ and RR of the all-soft OECD as functions of tensile strain (I) and the number of repeated strain cycles at 30% tensile strain (J) applied perpendicular to the channel length direction. (**K**) Current responses measured at constant biases of +1 V (solid lines) and − 1 V (dashed lines) (top), and the corresponding RR values (bottom), under tensile strains of 0, 15, and 30%, and after release, with strain applied perpendicular to the channel length direction.

The electrical characteristics of the all-soft OECD were evaluated under uniaxial stretching applied parallel and perpendicular to the channel length direction to examine their mechanical robustness. Representative current-voltage (*I-V*) characteristics measured under different tensile strains up to 30% applied parallel to the channel length direction are shown in Fig. 2D, demonstrating robust diode operation under mechanical deformation. This strain range was selected as a reproducible and practically relevant testing condition for wearable soft electronics, rather than as a fundamental mechanical limit of the device platform. The corresponding variations in *I*_F_ and RR extracted from the *I-V* curves are plotted in Fig. 2E. Although both parameters show a modest decrease with increasing strain up to 30%, the values largely recover upon strain release, indicating reversible and strain-tolerant operation. This strain-tolerant rectification is attributed to the optimized ultrathin ion-gel, which preserves the effective electrochemically active DoA by maintaining complete coverage of the target active region under tensile strain (fig. S10).

Mechanical durability was further assessed through cyclic stretching tests at 30% strain. As shown in Fig. 2F and fig. S17, both *I*_F_ and RR remain stable over 1000 stretching cycles with only minor degradation, confirming reliable operation under repeated mechanical loading. To evaluate operational stability under sustained electrical bias, time-dependent currents were measured under constant forward and reverse biases at different strain levels. Figure 2G shows that, after an initial electrochemical charging transient, the current stabilizes and the RR remains stable over time across different applied strain levels, confirming stable diode operation under prolonged bias and mechanical deformation. Square-wave bias measurements further clarified the electrochemical response regime of the all-soft OECD under strain (fig. S18).

Comparable strain tolerance is observed under tensile strain applied perpendicular to the channel length direction. The corresponding electrical characteristics remain stable under deformation (Fig. 2, H to J, and fig. S19), and the time-dependent responses under constant bias similarly exhibit negligible time dependence at various strains (Fig. 2K and fig. S20). These results establish the all-soft OECD as a mechanically robust and electrically reliable electrochemical diode platform, overcoming the material and architectural limitations of previously reported soft diodes and enabling fully deformable systems suitable for future human-centric and skin-interfaced applications.

### All-soft rectifiers

Rectifiers convert alternating electrical signals into unidirectional outputs, serving as fundamental building blocks for signal conditioning and power management in electronic systems (*43*). In human-centric electronics, rectification is particularly important for achieving stable and reliable operation in mechanically compliant, skin-conformal, and dynamically deformable devices that directly interface with the human body (*23*). Figure 3 (A and B) present the device architecture and circuit diagram of an all-soft half-wave rectifier composed of an OECD and a soft resistor, in which the entire circuit is constructed exclusively from soft and deformable components. The soft resistor was prepared by drop-casting a P3HT-NFs/PDMS composite onto an AgNWs/PDMS electrode with a predefined channel geometry. The corresponding device image and terminal configuration are shown in Fig. 3C, confirming a well-defined and fully soft rectifier structure. Owing to the intrinsic mechanical compliance of the constituent materials, the half-wave rectifier preserves its structural integrity under tensile deformation, as evidenced by optical images obtained in the pristine and stretched states (Fig. 3D). Furthermore, an all-soft full-wave rectifier was further fabricated by integrating four all-soft OECDs into a bridge configuration with a soft resistor, as shown in Fig. 3 (E and F). The corresponding device layout and terminal configuration are shown in Fig. 3G, while optical images in Fig. 3H confirm stable device geometry under applied tensile strain.

**Fig. 3.**
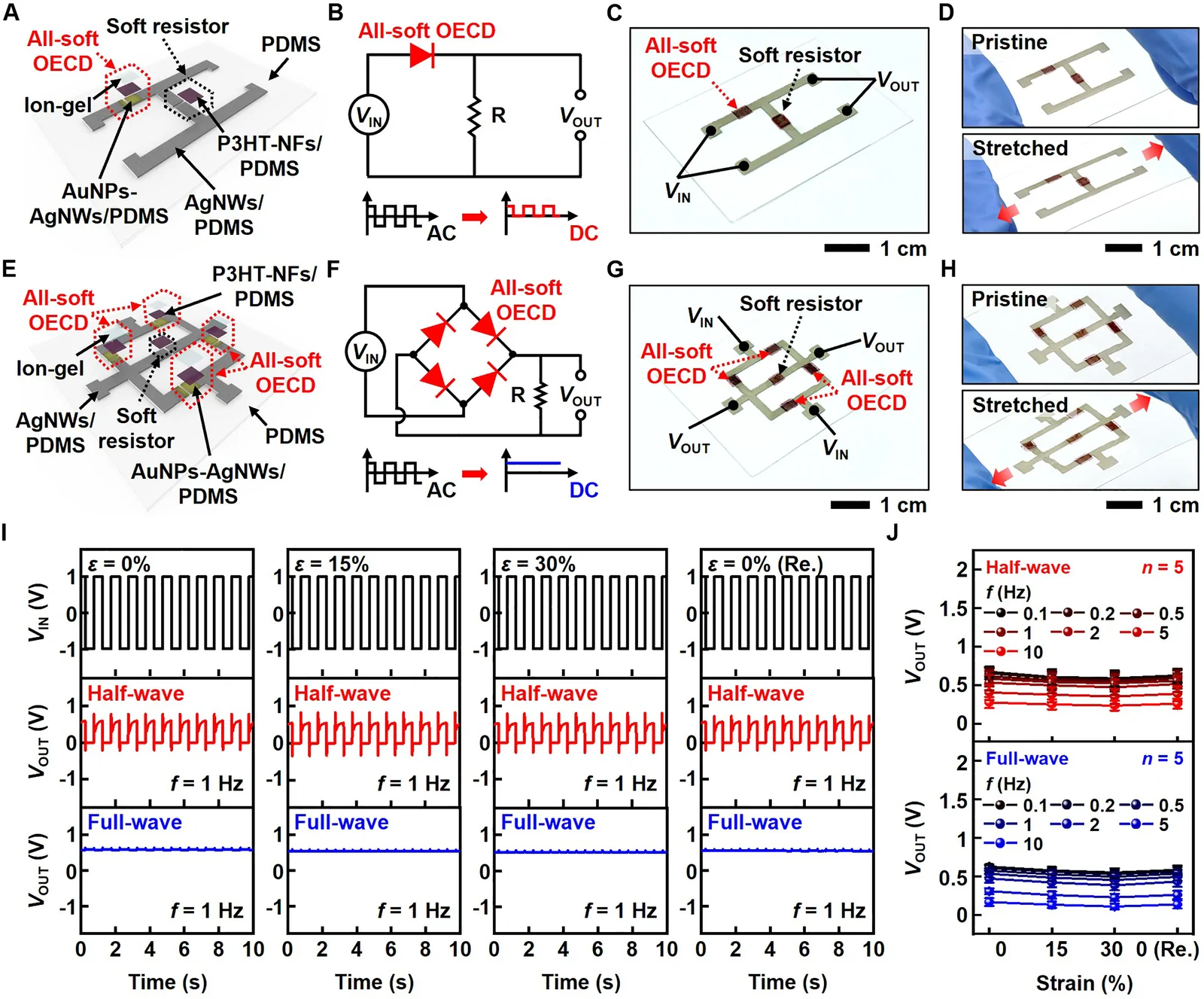
All-soft rectifiers based on the OECD. (**A** and **B**) Exploded schematic illustration (A) and circuit diagram (B) of the all-soft half-wave rectifier. (**C** and **D**) Optical images of the all-soft half-wave rectifier (C), and the device in the pristine and stretched states (D). (**E** and **F**) Exploded schematic illustration (E) and circuit diagram (F) of the all-soft full-wave rectifier. (**G** and **H**) Optical images of the all-soft full-wave rectifier (G), and the device in the pristine and stretched states (H). (**I**) Representative rectified output voltage (*V*_OUT_) signals of the all-soft half-wave and full-wave rectifiers under tensile strains of 0, 15, and 30%, and after release, measured using a digital input voltage (*V*_IN_) of ±1 V at 1 Hz. (**J**) *V*_OUT_ of the all-soft half-wave rectifier (top) and full-wave rectifier (bottom) as a function of tensile strain at different frequencies of the digital input signal.

The rectifying performance of both half-wave and full-wave rectifiers was evaluated by applying an AC input voltage (*V*_IN_) with an amplitude of ±1 V. As shown in Fig. 3I, the half-wave and full-wave rectified output voltage (*V*_OUT_) signals are clearly observed and remain stable under tensile strains of 0, 15, and 30%, as well as after strain release. The frequency dependence of the rectified *V*_OUT_ was further investigated (fig. S21). As shown in Fig. 3J, although the *V*_OUT_ gradually decreases with increasing input frequency, reliable rectification is consistently maintained for both circuit configurations across the examined frequency range and strain levels. Similar configuration-dependent rectification behavior was also observed under sinusoidal *V*_IN_ of ±1 V, confirming stable operation under analog input conditions for both rectifiers (figs. S22 to S24). The extracted cutoff frequencies further clarify the low-frequency operating regime of both rectifiers (fig. S25), while higher bandwidth may be achieved by reducing ion-transport distance, lowering RC delay, and optimizing the active-layer and ion-gel geometries (*44*, *45*). These results demonstrate that the all-soft OECD retains mechanically deformable rectifying functionality while being readily integrated into more complex circuit architectures, reinforcing its significance as a key building block for power management in future human-centric electronic systems.

### All-soft logic gates

Boolean operations are fundamental to digital signal processing in modern electronic systems and are implemented using logic gates (*46*). Although logic circuits are predominantly realized using transistor-based CMOS architectures in conventional electronics, simpler logic architectures can be advantageous in soft electronic systems. Thus, all-soft diode-based logic gates offer reduced circuit complexity and therefore become attractive for human-centric electronic systems in which mechanical compliance is prioritized over high integration density (*25*). Figure 4 (A and B) shows the schematic illustrations of device architecture and circuit diagram of an all-soft OR gate constructed by integrating two all-soft OECDs with a soft resistor. An optical image of the fabricated device is presented in Fig. 4C, where two independent input nodes (*V*_IN,A_ and *V*_IN,B_), a single output node (*V*_OUT_), and a common ground (GND) are clearly defined. Owing to the intrinsic mechanical compliance of all constituent materials, the OR gate preserves its structural integrity under tensile deformation, as shown in Fig. 4D. The logic operation of the all-soft OR gate was evaluated by applying digital input voltages of 0 and 1 V to *V*_IN,A_ and *V*_IN,B_ under different tensile strains. These voltage levels directly represent logic “0” and “1”, enabling straightforward evaluation of logic behavior. As shown in Fig. 4E, the *V*_OUT_ follows the expected OR logic response for all input combinations (0,0), (1,0), (0,1), and (1,1). This behavior is consistently maintained under tensile strains of 0, 15, and 30%, as well as after strain release. The strain-dependent *V*_OUT_ levels corresponding to each input condition are plotted in Fig. 4F, and the resulting truth table is summarized in fig. S26A, confirming that the logic states remain clearly distinguishable despite minor variations in output amplitude under mechanical deformation.

**Fig. 4.**
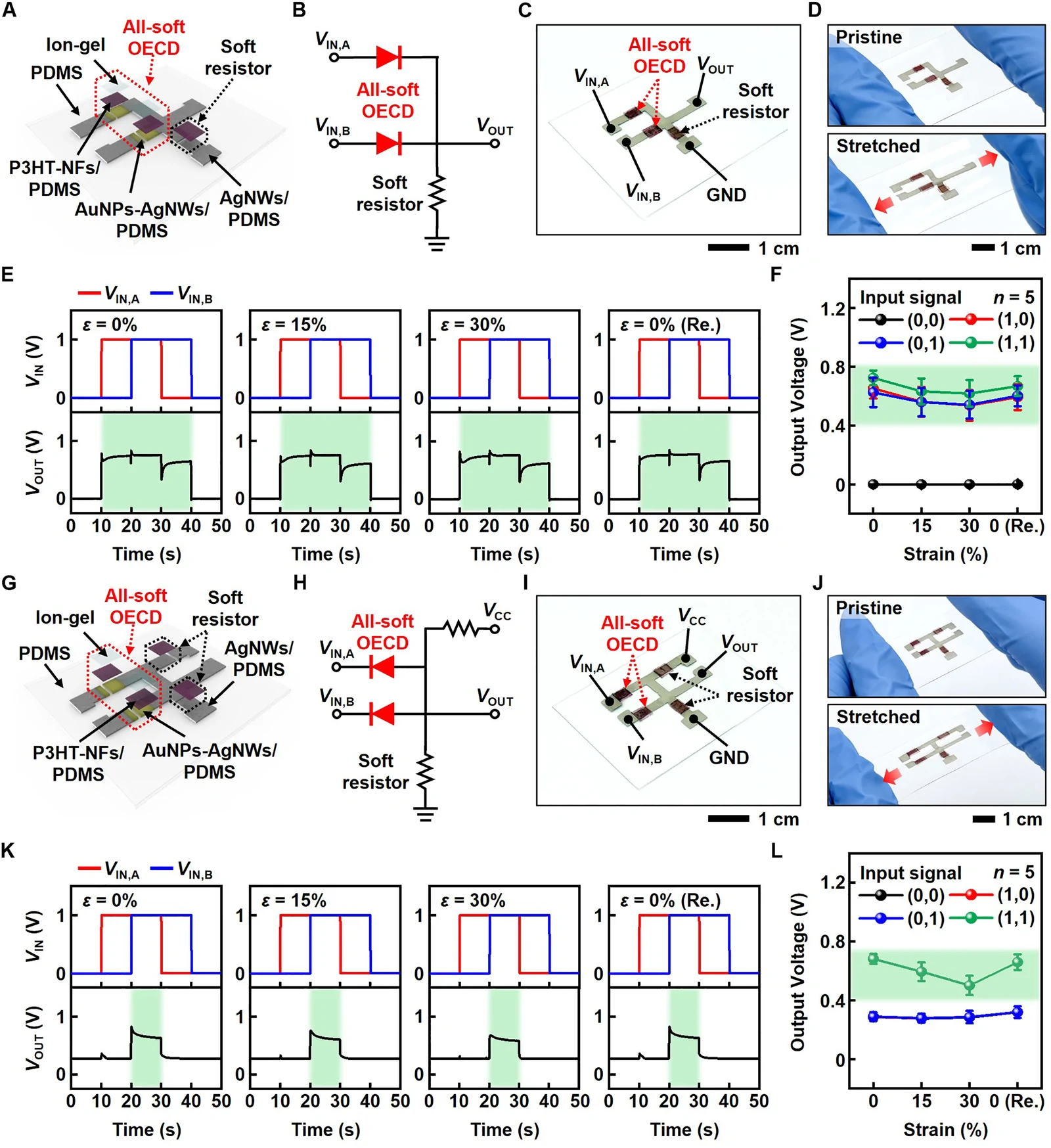
All-soft logic gates based on the OECD. (**A** and **B**) Exploded schematic illustration (A) and circuit diagram (B) of the all-soft OR gate. (**C**) Optical image of the all-soft OR gate. (**D**) Optical images of the all-soft OR gate in the pristine and stretched states. (**E**) Representative digital input voltages and corresponding output voltage signals of the all-soft OR gate under tensile strains of 0, 15, and 30%, and after release. (**F**) Output voltages of the all-soft OR gate as a function of tensile strain for different input signal combinations. (**G** and **H**) Exploded schematic illustration (G) and circuit diagram (H) of the all-soft AND gate. (**I**) Optical image of the all-soft AND gate. (**J**) Optical images of the all-soft AND gate in the pristine and stretched states. (**K**) Representative digital input voltages and corresponding output voltage signals of the all-soft AND gate under tensile strains of 0, 15, and 30%, and after release. (**L**) Output voltages of the all-soft AND gate as a function of tensile strain for different input signal combinations.

An all-soft AND gate was further developed using the same material platform and fabrication strategy. The schematic illustration, corresponding circuit diagram, and optical image of the fabricated AND gate are shown in Fig. 4 (G to J), respectively. In this configuration, an external supply voltage (*V*_CC_) is applied through a soft resistor to enable AND logic operation. The logic performance of the all-soft AND gate is presented in Fig. 4K. A logic “1” output voltage is generated only when both *V*_IN,A_ and *V*_IN,B_ are set to 1 V, consistent with the expected AND logic behavior. This operation remains stable under tensile strains of 0, 15, and 30%, as well as after strain release. The corresponding strain-dependent *V*_OUT_ levels are plotted in Fig. 4L, and the truth table is shown in fig. S26B, demonstrating reliable preservation of logic states under mechanical deformation. These results demonstrate that all-soft OECDs enable robust Boolean logic operations at the circuit level while maintaining stable functionality under substantial tensile strain, highlighting their potential as deformable signal-processing elements for future human-centric electronic systems.

### Cross-talk suppression in deformable sensor arrays

In multiplexed soft sensor arrays, electrical coupling between adjacent pixels can create unintended current pathways, commonly referred to as sneak paths. These parasitic pathways generate output signals from unaddressed pixels and consequently reduce the spatial accuracy of the array. Incorporating a diode into each sensing unit can regulate the direction of current flow and electrically isolate the addressed pixel, as illustrated by the representative circuit configuration in fig. S27. Therefore, the all-soft OECD serves as a key element for isolating individual sensing units in deformable sensor arrays.

As a proof-of-concept for practical applicability, we demonstrated an all-soft OECD-integrated pressure-sensing pixel designed to emulate a deformable sensor-array architecture. The unit pixel is composed entirely of soft electronic components, including an all-soft OECD, an all-soft OECT, an all-soft pressure sensor based on an ion-gel-coated porous PDMS sensing layer fixed with PDMS adhesive on AuNPs-AgNWs/PDMS electrode, and an all-soft resistor, as shown in Fig. 5A. In this configuration, the soft resistor and pressure sensor form a voltage divider that generates a pressure-dependent gating signal. The OECT actively reads out this high-impedance signal as an amplified current output with reduced sensitivity to downstream loading, while the OECD blocks unintended signal propagation through sneak paths. The fabricated device with clearly defined terminals is shown in Fig. 5B, and the corresponding circuit schematic is presented in Fig. 5C. In this unit pixel, the soft resistor was formed using P3HT-NFs/PDMS (*17*, *24*, *25*), while the pressure sensor was prepared using an ion-gel-coated porous PDMS structure (figs. S28 to S30) (*16*, *47*). The fabricated pressure sensor exhibits linear pressure-dependent responses up to 10 kPa and maintains its sensing characteristics under tensile strain (fig. S31). In addition, stable voltage-divider behavior was confirmed, and optimized output characteristics were obtained under an appropriate bias condition (fig. S32).

**Fig. 5.**
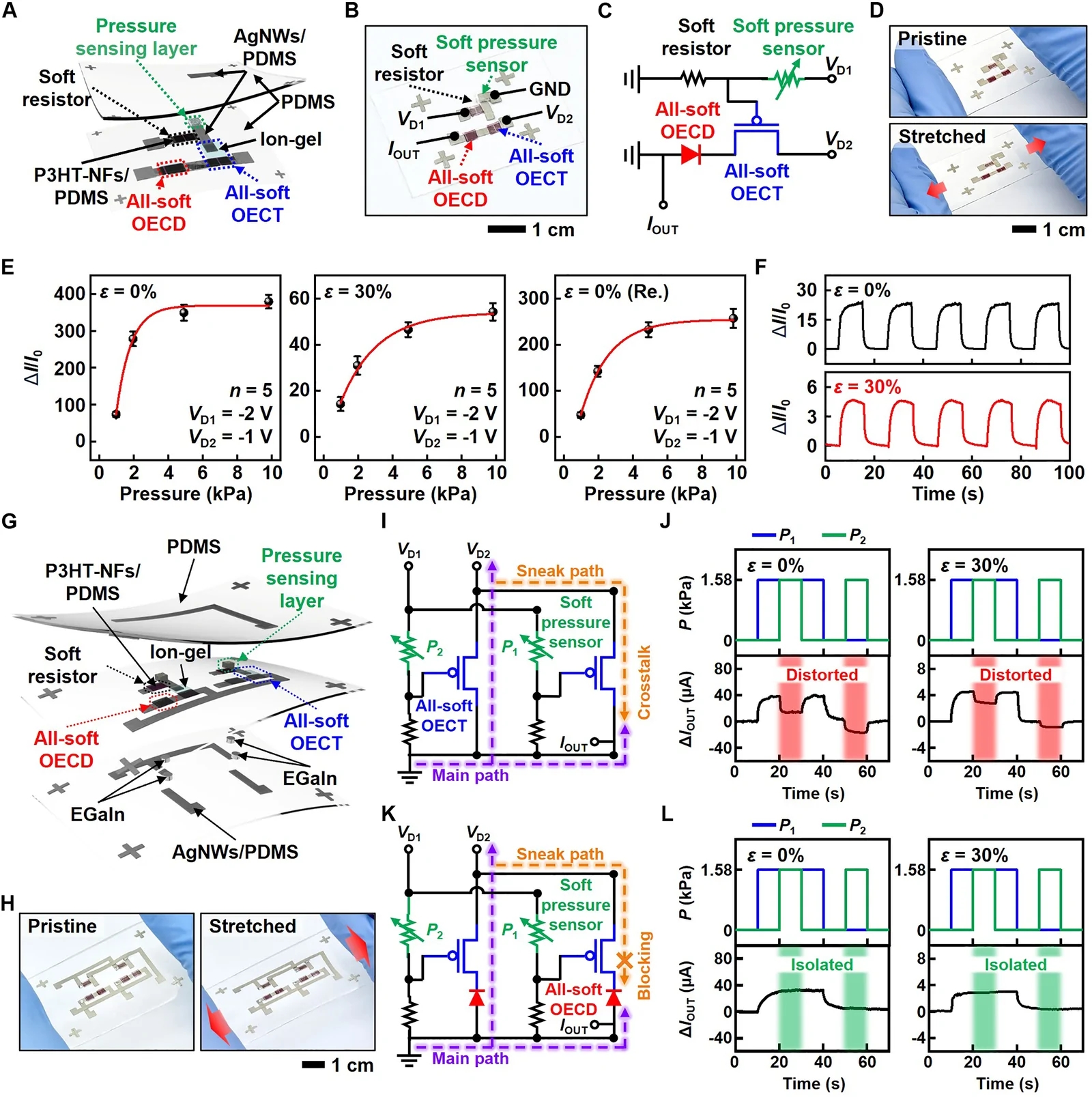
All-soft OECD-integrated pressure-sensing pixels with cross-talk suppression. (**A**) Exploded schematic illustration of the all-soft pressure-sensing pixel composed of an all-soft OECD, all-soft OECT, soft pressure sensor, and soft resistor. (**B**) Optical image of the fabricated sensing pixel. (**C**) Circuit diagram of the integrated device architecture. (**D**) Optical images of the sensing pixel in the pristine and stretched states. (**E**) Relative current change (Δ*I*/*I*_0_) of the sensing pixel as a function of applied pressure under tensile strains of 0 and 30%, and after release. (**F**) Dynamic pressure responses of the sensing pixel under a repeated pressure of 1.58 kPa at 0 and 30% tensile strain, measured at V_D1_ and V_D2_ of −2 and − 1 V, respectively. (**G**) Exploded schematic illustration of a two-pixel deformable sensing architecture for evaluating cross-talk suppression. (**H**) Optical images of the two-pixel device and its stretched state. (**I**) Circuit diagram of the two-pixel system without the all-soft OECD, illustrating sneak-path formation. (**J**) Applied pressure sequences (top) and measured output current changes (bottom) for the two-pixel system without the all-soft OECD at 0 and 30% tensile strain, showing distorted outputs caused by cross-talk. (**K**) Circuit diagram of the two-pixel system with the all-soft OECD, blocking sneak path. (**L**) Applied pressure sequences (top) and measured output current changes (bottom) for the two-pixel system with the all-soft OECD at 0 and 30% tensile strain, showing cross-talk-free operation.

To verify the role of the all-soft OECD within the integrated system, we first examined its electrical interaction with the all-soft OECT. When the OECD is connected in the forward direction, the OECT retains its normal transfer characteristics without substantial degradation (fig. S33). In contrast, reverse connection of the OECD effectively suppresses current flow, confirming its function as a directional blocking element against unintended signal transmission (fig. S34). Importantly, these behaviors remain stable under tensile strain, demonstrating robust electrical compatibility among the integrated soft components (fig. S35).

The integrated sensing pixel maintains its structural integrity under mechanical deformation without noticeable damage, as shown in Fig. 5D. The pressure-sensing characteristics of the integrated pixel were further optimized with respect to the operating bias conditions (fig. S36). Figure 5E shows that the calibration curves of the sensing pixel exhibit clear pressure-dependent modulation, and this sensing behavior is consistently maintained under tensile strain up to 30%. Although a slight decrease is observed under strain, the sensing response remains clearly distinguishable, and further optimization is possible but beyond the scope of this study. The device also exhibits stable dynamic responses under repeated pressure, maintaining consistent performance over 100 pressure cycles under tensile strains of 0, and 30% (Fig. 5F and fig. S37).

To directly evaluate cross-talk suppression, we constructed a two-pixel configuration in which neighboring sensing units are electrically coupled, as schematically illustrated in Fig. 5G, with the detailed geometry and dimensions provided in fig. S38. An optical image with corresponding terminals of the fabricated two-pixel device is presented in fig. S39. The device maintains mechanical deformability under tensile strain, as shown in Fig. 5H. In the absence of the all-soft OECD, sneak paths form between adjacent pixels, leading to substantial cross-talk (Fig. 5I). Experimentally, this appears as output signals from the neighboring pixel even when pressure is applied to only one pixel, regardless of strain condition (Fig. 5J). In contrast, incorporation of the all-soft OECD effectively blocks these sneak paths (Fig. 5K), and the corresponding measurements confirm that the output signal remains confined to the addressed pixel without interference from the adjacent unit (Fig. 5L). Quantitatively, the cross-talk signal ratio, obtained by comparing the unintended and intended signal changes from the same measured pixel, decreased from 63.1% to 0.314% at 0% strain and from 40.0% to 0.697% at 30% strain after OECD integration. These results clearly demonstrate that the all-soft OECD functions as a key signal-isolation component in deformable soft electronic systems, enabling cross-talk-free operation while maintaining mechanical robustness and seamless integration with other soft electronic components.

## DISCUSSION

We have successfully developed an all-soft OECD that employs mechanical deformability with high rectification performance at low operating voltages. Through the combination of an asymmetric active-layer geometry and a conformal ultrathin ion-gel interface, the device preserves the electrochemical asymmetry required for diode operation under tensile deformation. Distinct from conventional soft diodes based on interfacial barriers or liquid-electrolyte-mediated transport, this platform operates through bulk electrochemical doping in a solid-state configuration compatible with deformable systems and maintains stable electrical characteristics under strain and repeated cycling. Beyond the individual device, the all-soft OECD enables deformable rectifiers and logic gates and serves as a signal-isolation element that suppresses sneak-path-induced cross-talk in deformable sensor arrays. These results establish electrochemical diodes as practical circuit-level components for soft electronics and provide a robust framework for future integrated human-centric electronic systems.

## MATERIALS AND METHODS

### Materials

Dichloromethane (>99%), gold chloride trihydrate (HAuCl_4_·3H_2_O; >99.9%), poly(3-hexylthiophene-2,5-diyl) (P3HT; molecular = weight 50,000 – 100,000), poly(vinylidene fluoride-co-hexafluoropropylene), (PVDF-HFP; molecular weight = ∼400,000), acetone (>99%), 1-Ethyl-3-methylimidazolium bis(trifluoromethylsulfonyl)imide (EMIM-TFSI), Diiodomethane (DIM; >99%), and eutectic gallium-indium (EGaIn) were purchased from Sigma-Aldrich. Silver nanowires (AgNWs) ink (average diameter = 75–105 nm, average length = 40–80 μm, 1 wt% in isopropyl alcohol) was purchased from Zhejiang Kechuang Advanced Materials. Polydimethylsiloxane (PDMS; Sylgard 184 silicone elastomer kit) was purchased from Dow-corning. Ammonia (NH_4_OH; 25.0–29.0%) was purchased from Duksan Chemicals corporation. Sodium chloride (NaCl; >99.5%) was purchased from Samchun Chemical corporation.

### Preparation of P3HT-NFs/PDMS soft semiconducting composite solution

P3HT was dissolved in dichloromethane at a concentration of 0.5 mg/ml by heating at 80°C for 20 min until a clear, transparent orange solution was obtained. The solution was then stored at −25°C for 1 hour to induce the formation of P3HT nanofibrils (P3HT-NFs) through self-assembly by π-π stacking (*37*, *48*). Separately, a PDMS precursor in dichloromethane (10:1 (w/w) prepolymer/curing agent, 20 mg/ml) was prepared and added to the P3HT-NFs solution at a volume ratio of 1:10, yielding a final P3HT-NFs/PDMS weight ratio of 1:4. The resulting P3HT-NFs/PDMS soft semiconducting composite solution was stored at −25°C until use.

### Preparation of AuNPs-AgNWs/PDMS soft electrode

To prepare the AuNPs-AgNWs/PDMS soft electrode, glass substrates were sequentially cleaned with acetone, IPA, and deionized (DI) water, followed by dehydration on a hot plate at 110°C for 5 min. A polyimide tape-based shadow mask patterned using a programmable cutting machine (Silhouette Portrait 3) was then laminated onto the substrates. AgNWs ink was drop-cast onto the masked substrates and dried at 60°C for 20 min. After removal of the shadow mask, the patterned AgNWs were annealed at 200°C for 30 min to improve conductivity, and the electrode channels were subsequently defined using a razor blade. Degassed PDMS precursor (10:1 (w/w) prepolymer/curing agent) was spin-cast onto the patterned AgNWs at 300 rpm for 60 s and cured at 100°C for 1 hour. Because the viscous PDMS precursor penetrated the porous AgNWs network while the nanowires remained more densely distributed near the bottom surface, a partially embedded AgNWs/PDMS electrode was obtained after peeling off the cured film from the glass substrate. To form AuNPs on AgNWs, 0.5 mM HAuCl_4_ solution was drop-cast onto the exposed AgNWs channels and allowed to react for 4 min via galvanic replacement (*38*). The electrode was then sequentially immersed in aqueous NH_4_OH and DI water for 1 min each to remove the AgCl byproduct, followed by N_2_-assisted drying and dehydration on a hot plate at 110°C for 5 min to complete the preparation of the AuNPs-AgNWs/PDMS soft electrode.

### Fabrication of the all-soft OECD, rectifiers, and logic gates

The all-soft OECD was fabricated using first preparing the AuNPs-AgNWs/PDMS soft electrode as both the anode and cathode. The sonicated P3HT-NFs/PDMS soft semiconducting composite solution was then drop-cast onto the channel region through an asymmetrically detached polyimide film-based shadow mask to form a patterned soft semiconducting layer. The sample was subsequently annealed at 110°C for 20 min. Separately, an ion-gel precursor solution composed of PVDF-HFP, dehydrated EMIM-TFSI, and acetone (1:4:7 (w/w)) was prepared by magnetic stirring at 70°C for 1 hour. The homogeneous ion-gel precursor was spin-coated at 2000 rpm for 60 s onto a custom-made UV-O_3_-treated PDMS stamp prepared by curing a 10:1 (w/w) prepolymer/curing agent mixture. The coated stamp was then placed onto the P3HT-NFs/PDMS layer and maintained in contact for at least 1 s before gentle detachment of the PDMS stamp, leaving a thin ion-gel layer conformally transferred onto the channel to complete the fabrication of the all-soft OECD. The optimized ultrathin ion-gel was manually transfer-printed successfully for all devices in the tested batch (25/25 devices). For the 5 × 5 OECD array, the ultrathin ion-gel layers were sequentially transfer-printed onto individual OECDs to maintain uniform ion-gel thickness and coverage across the array. For the all-soft rectifiers and logic gates, the P3HT-NFs/PDMS composite was first drop-cast onto appropriately patterned soft electrodes to form the semiconducting layers of the all-soft OECDs and the soft resistors, followed by annealing at 110°C for 20 min. The ion-gel layer was then selectively transfer-printed onto the diode channels using the same method described above to complete the all-soft OECDs, which were subsequently integrated with the soft resistors to fabricate the all-soft rectifiers and logic gates.

### Fabrication of the all-soft pressure-sensing pixel

The all-soft pressure-sensing pixel unit was fabricated on appropriately patterned soft electrodes by integrating an ion-gel-coated porous PDMS structure used as the all-soft pressure sensor with the all-soft OECD and soft resistor prepared using the methods described above, together with an all-soft OECT fabricated by the same procedure as the all-soft OECD except that the P3HT-NFs/PDMS semiconducting layer was symmetrically patterned across the channel. For fabrication of the ion-gel-coated porous PDMS structure, finely ground NaCl powder was uniformly mixed with degassed PDMS precursor (10:1 (w/w) prepolymer/curing agent) at a weight ratio of 10:1 (NaCl/PDMS). The resulting mixture was cast onto a glass substrate and compressed using another glass substrate separated by spacers to form a film of uniform thickness, followed by curing at 60°C for 3 hours. The solidified PDMS/NaCl composite was then detached from the substrate and immersed in deionized water at 80°C for 72 hours to dissolve the NaCl template, with the water refreshed every 8 hours (*49*). The obtained porous PDMS was dried at 100°C for 1 hour, cut into 2 × 2 mm^2^ pieces, and immersed in a preheated ion-gel precursor solution composed of PVDF-HFP, dehydrated EMIM-TFSI, and acetone [1:4:7 (w/w)] for 1 min. Excess precursor was then removed using tweezers and dust-free tissue, followed by drying for 1 min to form the ion-gel-coated porous PDMS structure. The prepared porous structure was then placed onto the designated gate terminal region and fixed using PDMS adhesive. Finally, a soft AgNWs/PDMS top electrode was laminated onto the pressure sensor to complete the fabrication of the all-soft pressure-sensing pixel.

### Characterization

All *I*-*V* measurements of the diodes were carried out using a semiconductor analyzer (4200-SCS, Keithley Instruments) with a probe station (MST 8000 C, MS Tech.) and a custom-made stretcher to characterize the performance under different tensile strains. The ionic conductivities of the EMIM-TFSI electrolyte and the ion-gel were measured using a potentiostat (CHI 604E, CH Instruments) in a two-electrode system, with ITO glass serving as the electrode. Electrochemical impedance spectroscopy (EIS) was subsequently performed using the same equipment in a three-electrode system, comprising a drop-cast P3HT-NFs/PDMS film-coated soft AuNPs-AgNWs/PDMS, Pt wire, and Ag/AgCl (3 M KCl) as working, counter and reference electrodes, respectively. The spectrum was analyzed by ZView software to evaluate the capacitance by the fitted equivalent circuit *R*_s_(*R*_p_||*Q*), where *R*_s_, *R*_p_ and *Q* represent a series resistor, a parallel resistor, and a parallel constant phase element (*50*). The surface morphology of the gel-coated porous PDMS was examined using field emission scanning electron microscopy (FE-SEM, SU7000, Hitachi). The OR, AND logic gates, rectifiers, pressure sensor, and the all-soft pressure-sensing pixel were characterized by Keysight B2912B (Keysight Technologies Inc.) and TBS2104B Digital Oscilloscope (Tektronix Inc.) with the applied voltages from a D.C. power supply (GPS-3303, GW Instek), and a function generator (DG4102, RIGOL Technologies). The pressure sensitivity of the pressure sensor and transfer characteristics of the all-soft pressure-sensing pixel were obtained using a force gauge (M7–50, Mark-10 Corp.).

## Supplementary Materials

### This PDF file includes:

Note S1 Tables S1 and S2 Figs. S1 to S39 References
