# Supplementary material for: All-soft organic electrochemical diodes based on asymmetric active layers for signal isolation in deformable electronics
Source: Sci Adv. 2026 Oct 21;12(43):eaei3178. doi: 10.1126/sciadv.aei3178 (PMC13685924; doi:10.1126/sciadv.aei3178)
Supplement: Supplementary file 1 [file sciadv.aei3178_sm.pdf]

Science Advances
AAAS

Supplementary Materials for

All-soft organic electrochemical diodes based on asymmetric active layers for signal isolation in deformable electronics

Sehyun Kim *et al.*

Corresponding author: Kyoseung Sim, kyos@unist.ac.kr

Sci. Adv. **12**, eaei3178 (2026)
DOI: 10.1126/sciadv.aei3178

This PDF file includes:

Note S1
Tables S1 and S2
Figs. S1 to S39
References

Supplementary Text

Note S1. Calculation of surface energy

The surface energies of bare PDMS, UV-O_3 treated PDMS, PVDF-HFP, and P3HT-NFs/PDMS were evaluated by contact angle measurements using Owens-Wendt model, which separates the total surface energy into polar and dispersive components, as expressed by the following equations (S1) and (S2) (*51, 52*).

$$\gamma_L(1+\cos\theta) = 2\cdot\left(\sqrt{\gamma_S^D\cdot\gamma_L^D}+\sqrt{\gamma_S^P\cdot\gamma_L^P}\right) \tag{S1}$$

$$\gamma_S = \gamma_S^D + \gamma_S^P \tag{S2}$$

Here, θ is measured static contact angle measured using deionized (DI) water and diiodomethane (DIM) at room temperature after the droplet shape stabilized. γ_L is the total surface tension of the probe liquid and γ_L^D and γ_L^P are its dispersive and polar components, respectively, and the values of γ_L, γ_L^D, and γ_L^P were determined from reported values of 72.8, 21.8, and 51,0 mJ/m^2 for DI water and 50.8, 50.8, and 0 mJ/m^2 for DIM, respectively (*52, 53*). γ_S is the total solid surface energy, and γ_S^D and γ_S^P are its dispersive and polar components for bare PDMS, UV-O_3 treated PDMS, PVDF-HFP, and P3HT-NFs/PDMS, as summarized in Table S1 and Fig. 1E.

Supplementary Figures

Table S1. Solid polar (γ_S^P) and dispersive (γ_S^D) components, and surface energy (γ_S) of PDMS stamp, PVDF-HFP, and P3HT-NFs/PDMS.

Surface	γ_S^D	γ_S^P	γ_S
**PDMS stamp**	**25.3**	**13.0**	**38.3**
**PVDF-HFP**	**19.6**	**0.0104**	**19.6**
**P3HT-NFs/PDMS**	**27.7**	**0.0616**	**27.8**

Table S2. Comparison of diode performance of the all-soft OECD with previously reported all-soft diodes for deformable electronics.

Types of diode	Active medium	Applied voltage (V)	Rectification ratio (RR)	Ref
Electrolyte	PVDF-HFP/EMIM-TFSI/P3HT-NFs/PDMS	1	2.23×10^4	This work
Polymeric semiconductor	PEDOT:PSS:ION E/DPP4T-oSi10/PEDOT:PSS:PSSNa:ION E/PEIE	2	540	23
Polymeric semiconductor	AuNPs-AgNWs/P3HT-NFs/EGaIn	5	8.37×10^4	24
Polymeric semiconductor	PEDOT:PSS/P3HT-NFs/EGaIn	3	6.15×10^4	25
Electrolyte	PSPA/PDMAPAA-Q	4	31	26
Electrolyte	ES/AT	0.35	550	27
Electrolyte	PAAc(Mm^+)-co-PAAm/PDAC(Cl^-)-co-PAAm	5	140	28
Electrolyte	PAAm-PSS/PAAm-PDAC	4.5	18.5	39
Electrolyte	PIE/NIE	0.4	22	40
Electrolyte	CS/QC	4.5	123	41
Electrolyte	pBBEs/nBBEs	0.3	46	42

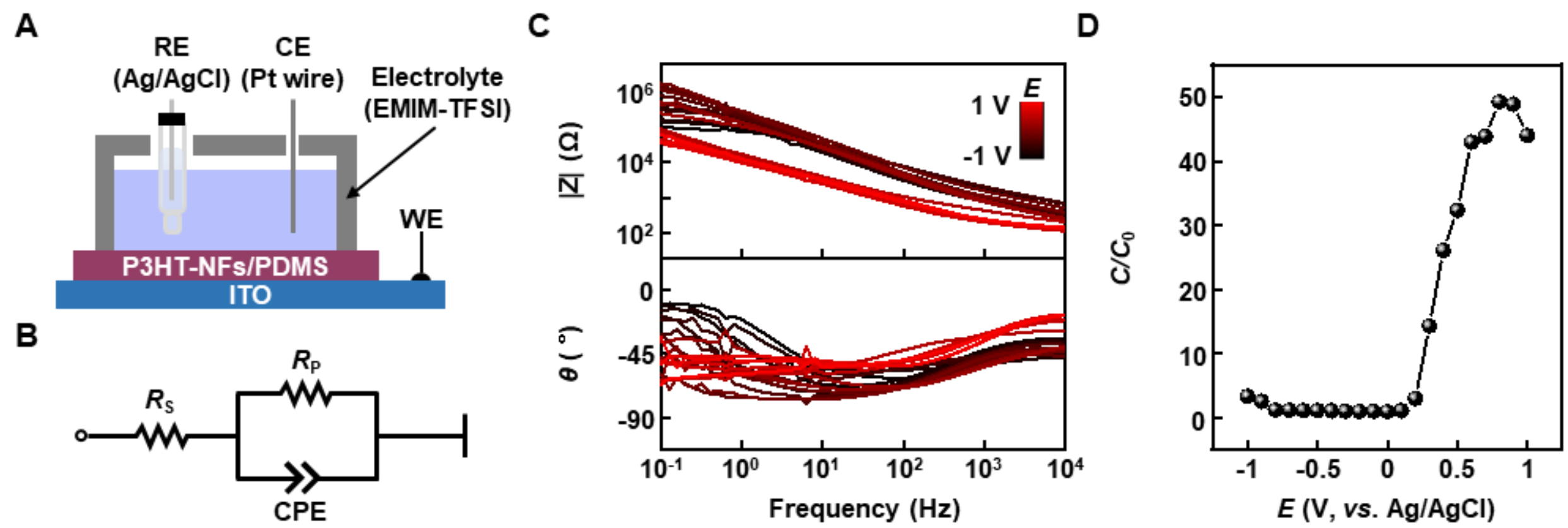

Fig. S1. Electrochemical impedance spectroscopy analysis of P3HT-NFs/PDMS with EMIM-TFSI. (**A**) Schematic illustration of EIS measurement configuration. (**B**) Equivalent circuit consisting of electrolyte resistance (R_S), and polarization resistance (R_P) and constant phase element (CPE) of P3HT-NFs/PDMS. (**C**) Bode (top) and phase (bottom) plots at different potential (*E*) ranging from -1 to 1 V. (**D**) Calculated C/C_0 (*C*: capacitance under *E*, C_0: capacitance under *E* of 0 V) as a function of *E*. The Bode and phase plots show systematic *E*-dependent changes in the impedance (|Z|) response, with lower |Z| values and a more pronounced capacitive phase response under positive *E* than under negative *E*, indicating enhanced electrochemical charging at positive potential. Consistent with this trend, the extracted C/C_0 markedly increases under positive *E*, reflecting anion-driven bulk electrochemical accumulation within the P3HT-NFs/PDMS layer, whereas the minimal C/C_0 variation under negative *E* indicates limited cation accumulation and surface-dominated charge compensation. These results indicate that ionic accommodation in the P3HT-NFs/PDMS layer is strongly polarity dependent over the investigated potential range (*54-56*).

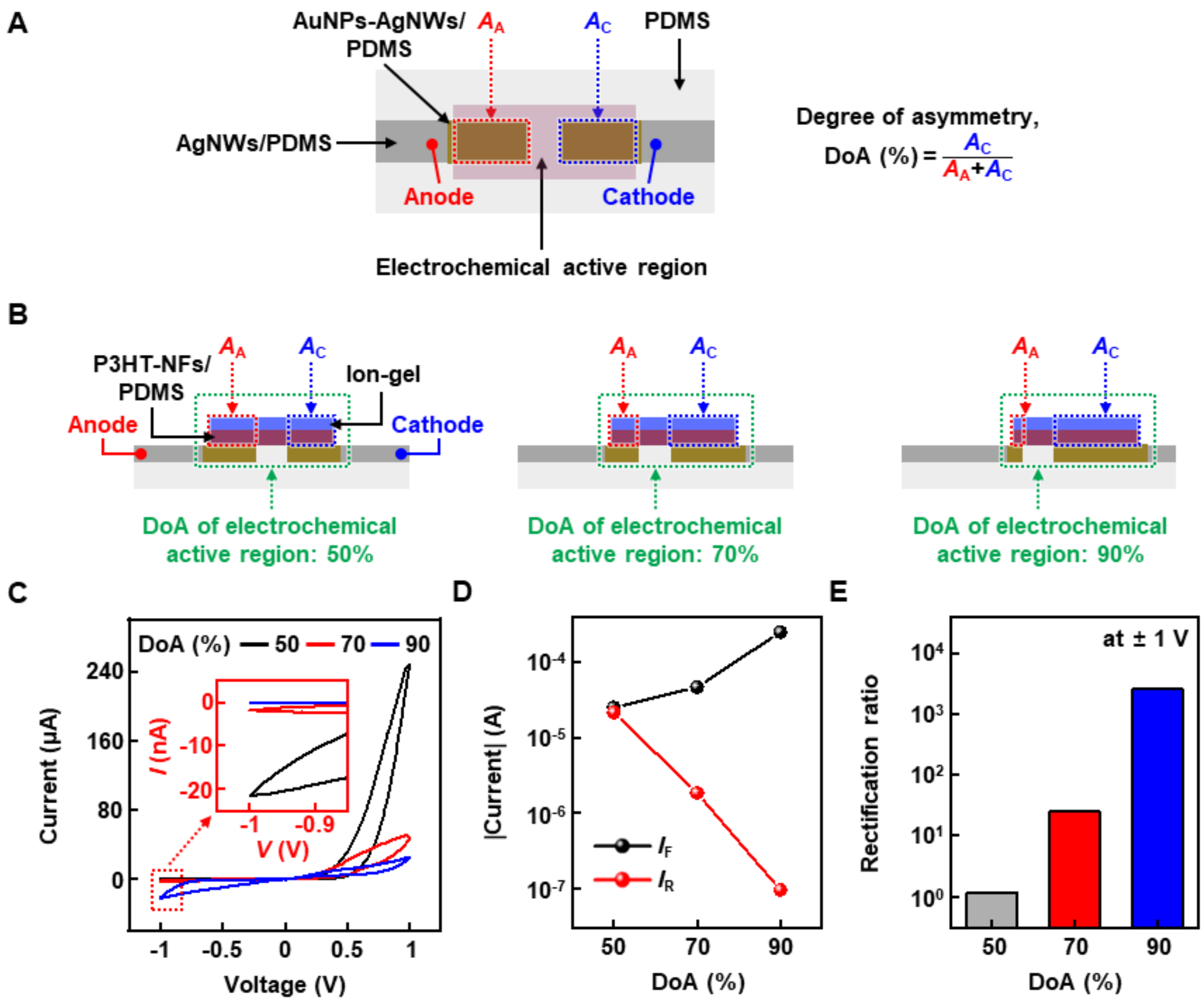

Fig. S2. The electrical characterization of DoA-dependent behavior of the all-soft OECDs. (**A**) Schematic illustration of defining a DoA (A_A: active-anode contact area, A_C: active-cathode contact area). (**B**) Schematic illustration of the device configurations with different DoAs of 50, 70, and 90%. (**C**) current-voltage (*I-V)* characteristics of the all-soft OECDs with different DoAs of 50, 70, and 90%. (**D and E**) Extracted I_F and I_R (I_F: forward current under +1 V, I_R: reverse current under -1 V) (D), and the resulting calculated RR (E) of the all-soft OECDs as a function of DoAs.

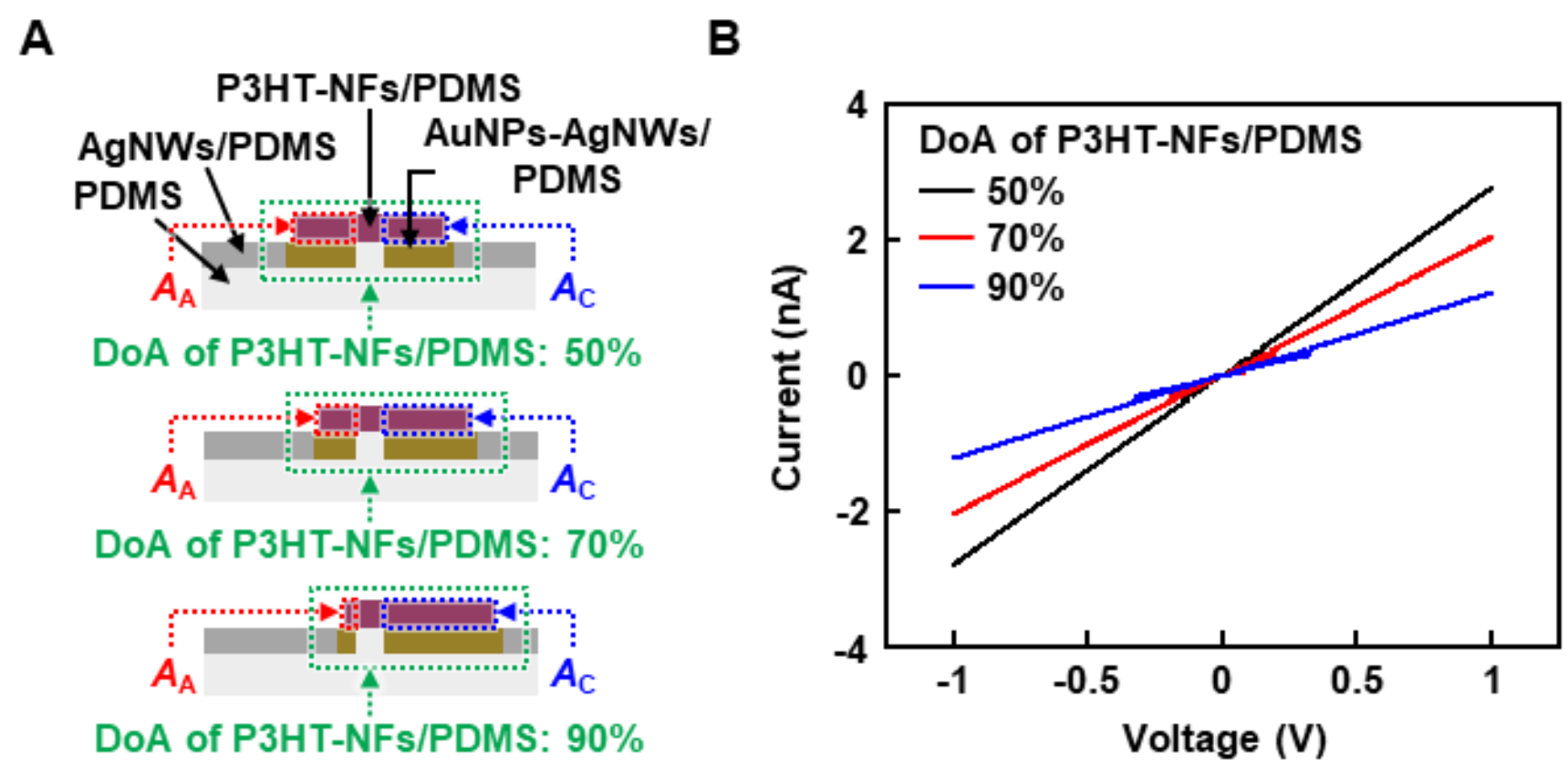

Fig. S3. The electrical characterization of DoA-dependent behavior of the all-soft OECDs without the ion-gel. (**A**) Schematic illustration of the device configurations with different DoAs of 50, 70, and 90%. (**B**) *I-V* characteristics of the devices with different DoAs of 50, 70, and 90%.

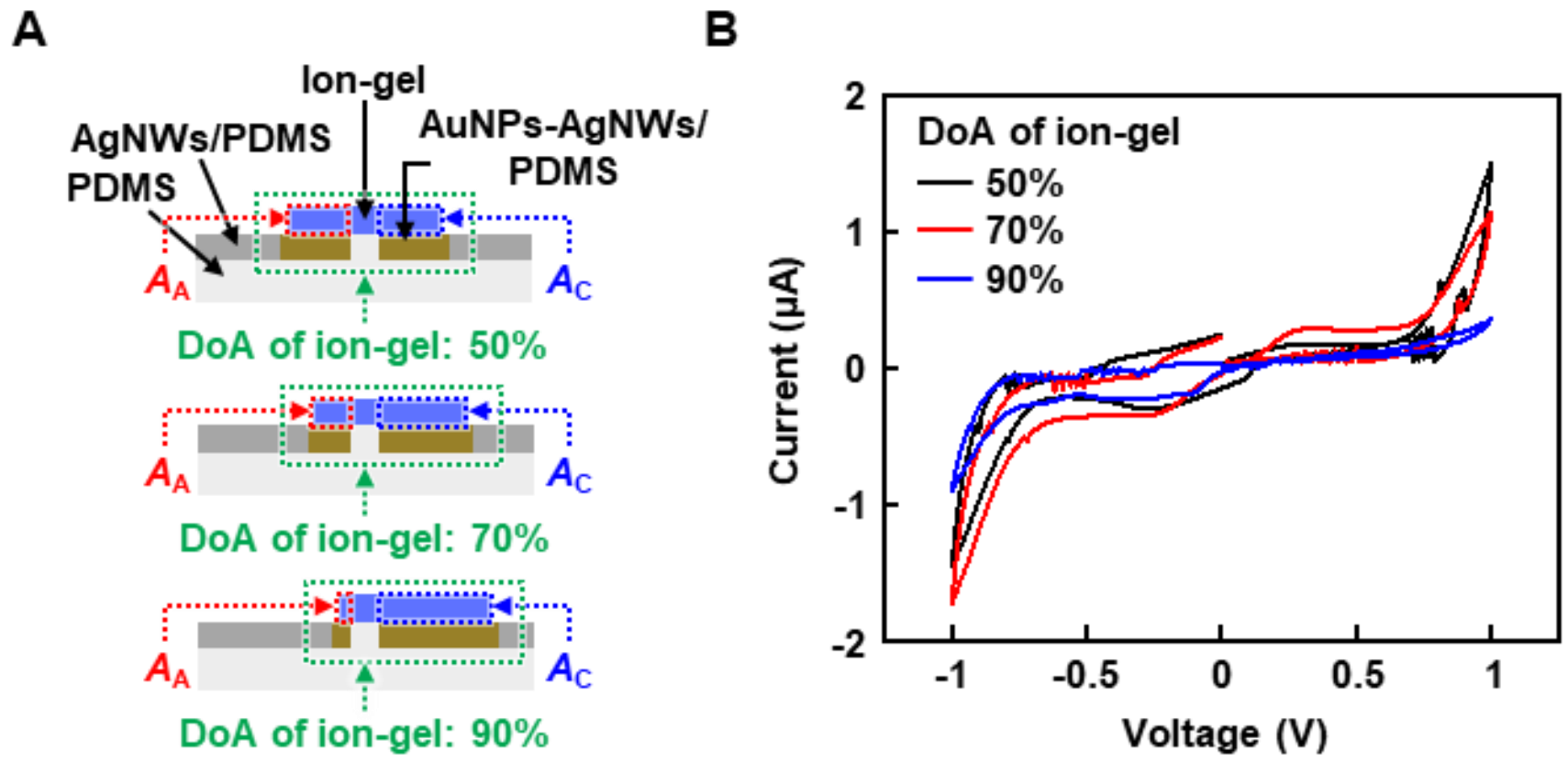

Fig. S4. The electrical characterization of DoA-dependent behavior of the all-soft OECDs without P3HT-NFs/PDMS. (**A**) Schematic illustration of the device configurations with different DoAs of 50, 70, and 90%. (**B**) *I-V* characteristics of the devices with different DoAs of 50, 70, and 90%.

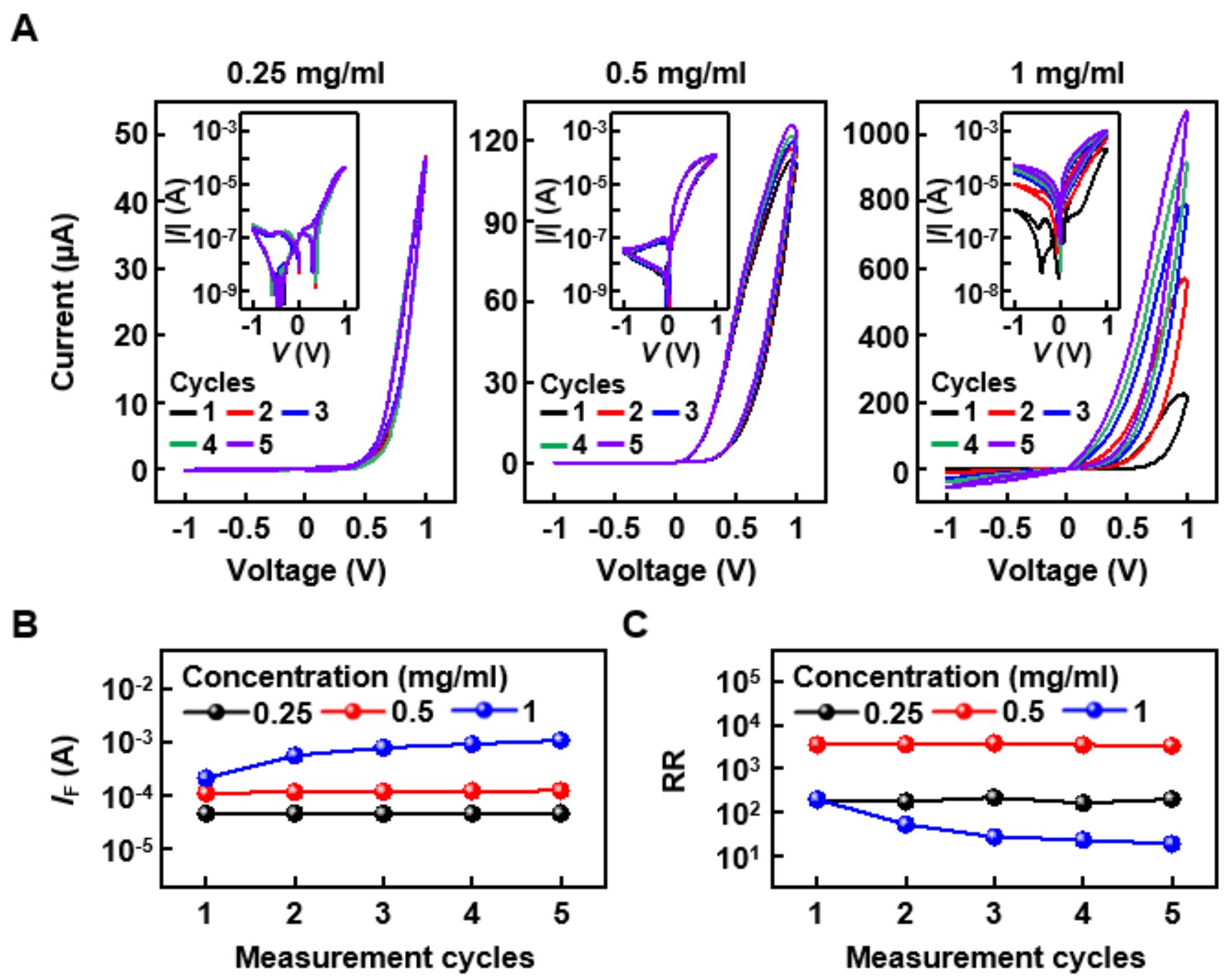

Fig. S5. Optimization of P3HT-NFs/PDMS solution concentration for all-soft OECDs. (**A**) *I*-*V* characteristics of all-soft OECDs prepared using different P3HT-NFs/PDMS solution concentration, corresponding to measured P3HT-NFs/PDMS active-layer thicknesses of 0.123, 0.286, and 0.534 µm, during five repeated voltage sweeps. (**B and C**) Extracted I_F (B) and RR (C) as functions of repeated sweep cycles for devices prepared using different P3HT-NFs/PDMS solution concentrations. The thickness of the P3HT-NFs/PDMS active layer in the all-soft OECD was measured using a three-dimensional optical surface profiler (NV-3000, Nanosystem).

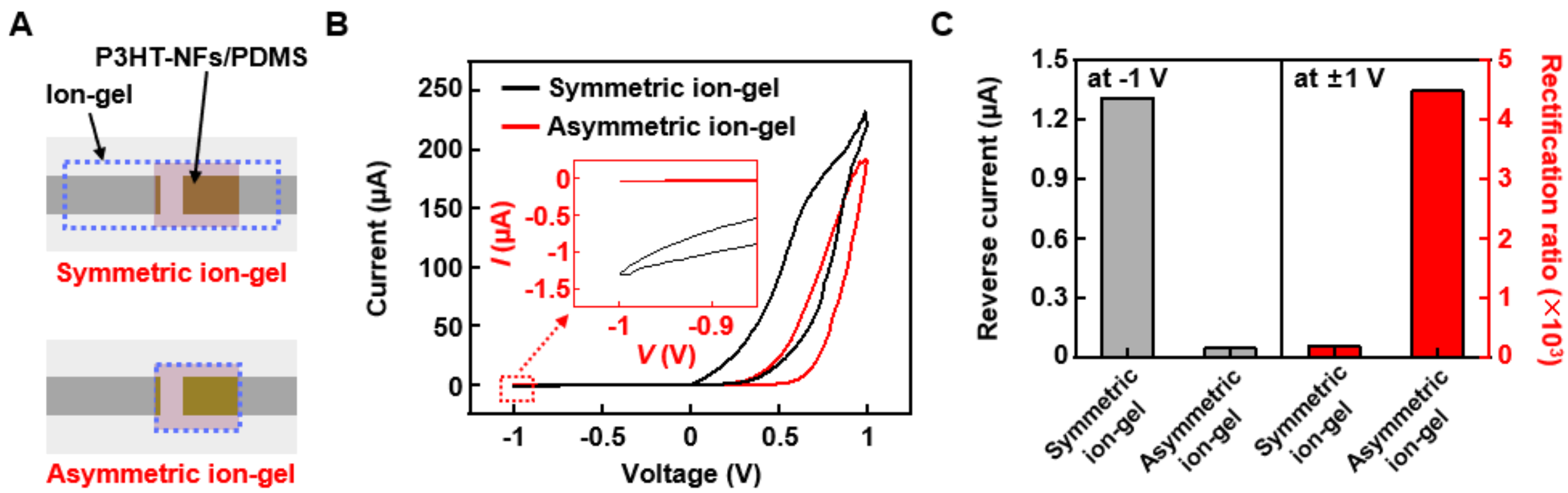

Fig. S6. Comparison of the all-soft OECD performance with symmetric and asymmetric ion-gel configurations. (**A**) Schematics of symmetric (top) and asymmetric (bottom) ion-gel configurations. (**B**) *I-V* characteristics of the symmetric and asymmetric ion-gel configurations. (**C**) Extracted I_R and calculated RR of symmetric and asymmetric ion-gel configurations. The symmetric ion-gel configuration enhances electrical double layer-induced capacitive currents at the electrode/ion-gel interface under reverse bias, resulting in an increased I_R and a reduced RR.

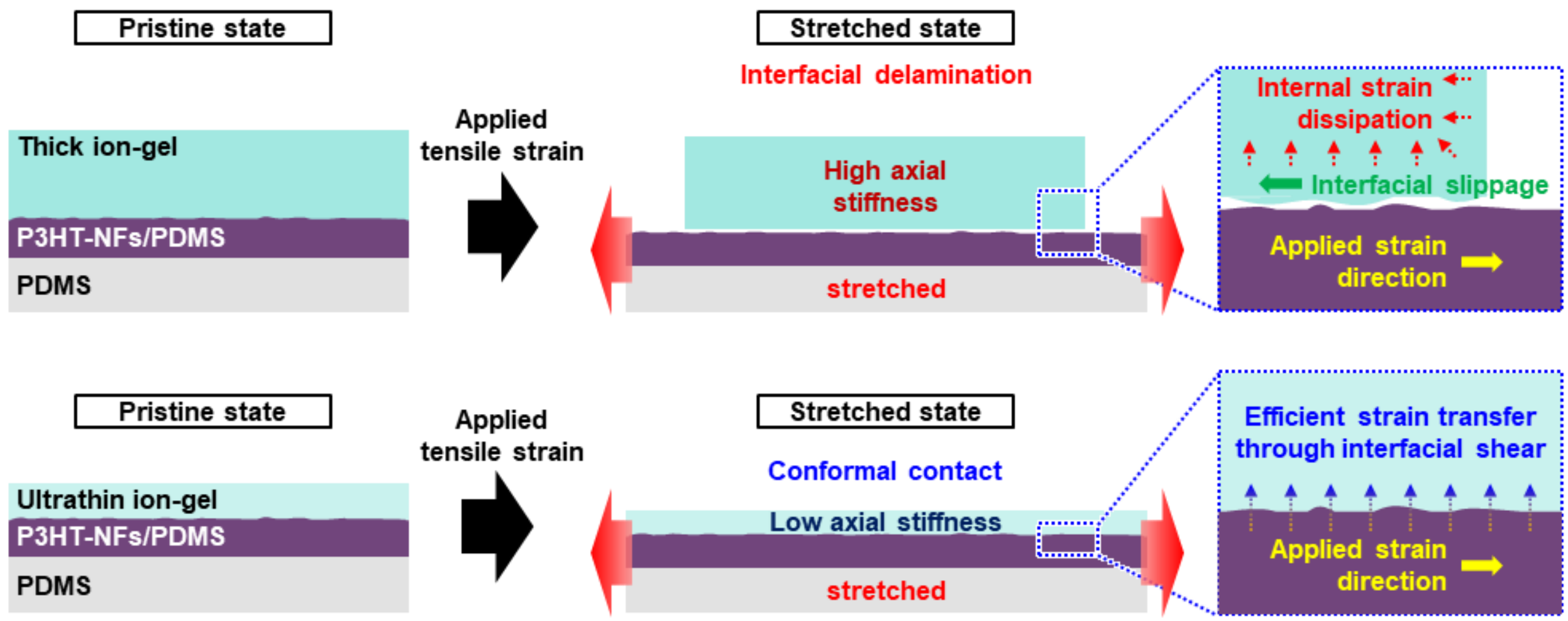

Fig. S7. Schematic illustration of the deformation behaviors of thick (top) and ultrathin (bottom) ion-gels under uniaxial stretching.

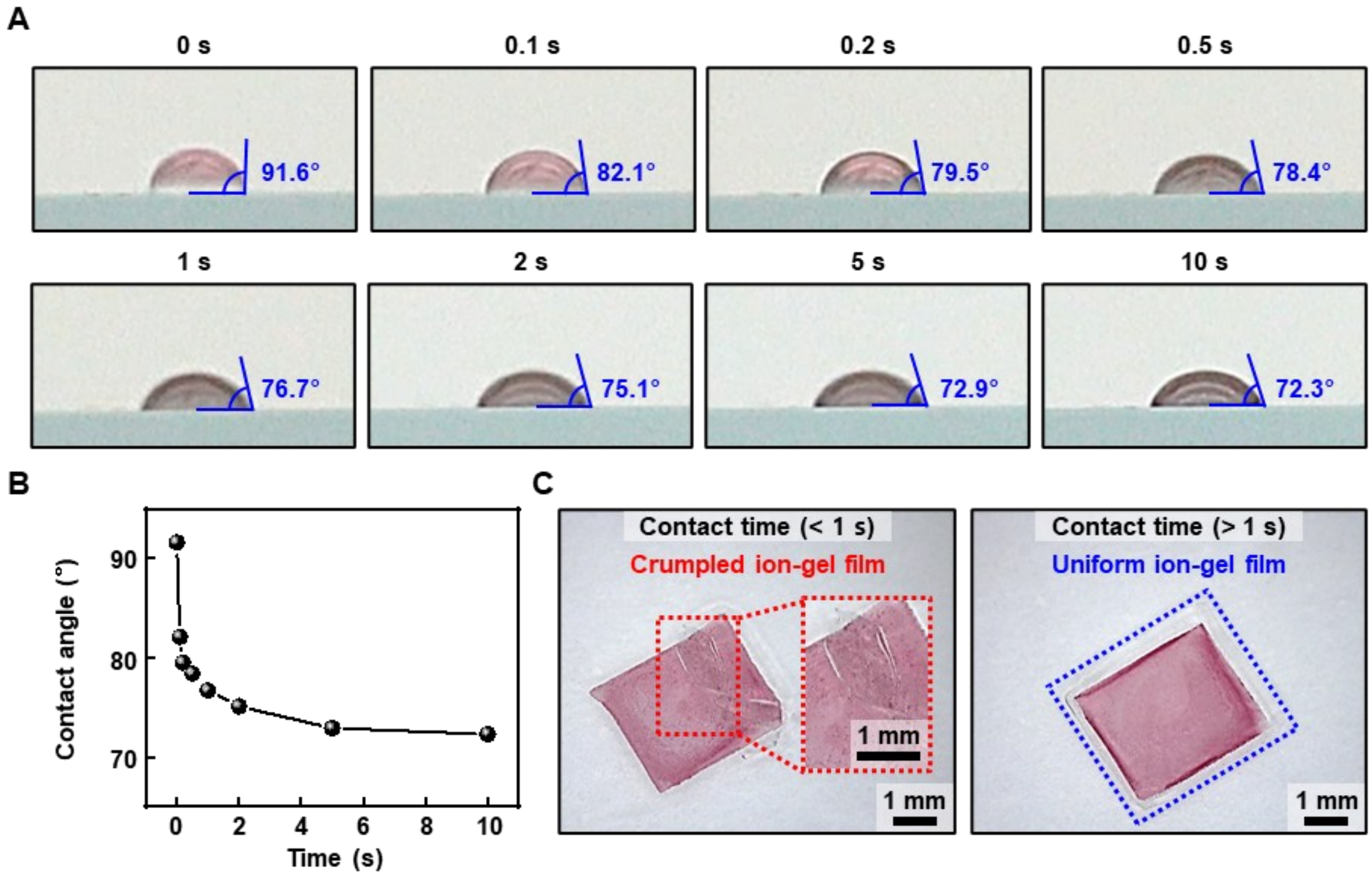

Fig. S8. Time-dependent wetting of the ionic liquid on the P3HT-NFs/PDMS layer for uniform ion-gel transfer. (**A**) Optical images of the ionic liquid droplets on the P3HT-NFs/PDMS layer at different times after dispensing, showing the evolution of the contact angle. (**B**) Extracted contact angle of the ionic liquid on the P3HT-NFs/PDMS layer as a function of time. (**C**) Optical microscopy images of transferred ion-gel films obtained by immediate peel off after stamp contact (left) and by peel off after a 1 s contact time (right). The gradual decrease in contact angle indicates progressive wetting of the ionic liquid on the P3HT-NFs/PDMS layer, which is expected to increase the effective interfacial contact for uniform transfer (*57*). Accordingly, providing sufficient contact time between the ion-gel and the P3HT-NFs/PDMS surface promotes ionic-liquid wetting, facilitates intimate interfacial contact, and thereby enables successful transfer of a uniform ion-gel film (*58*).

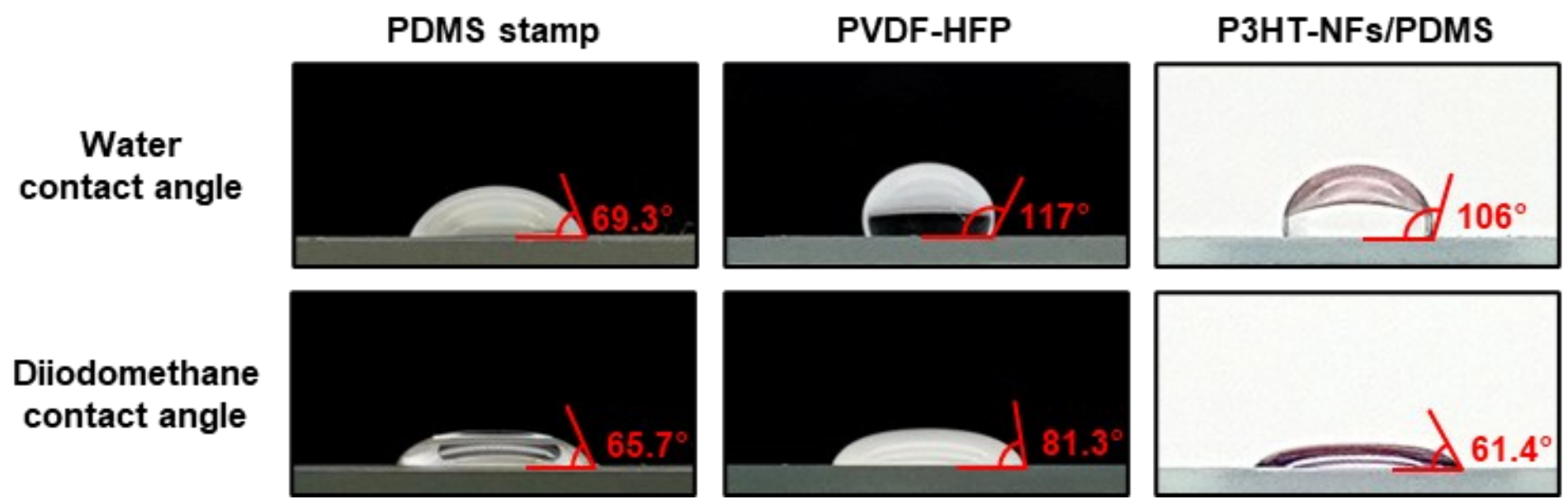

Fig. S9. Static contact angles of water and diiodomethane on PDMS stamp, PVDF-HFP, and P3HT-NFs/PDMS. These measurements were used to estimate the polar and dispersive components of solid surface free energy (γ_S^P and γ_S^D) and thereby determine the total surface energy of each surface.

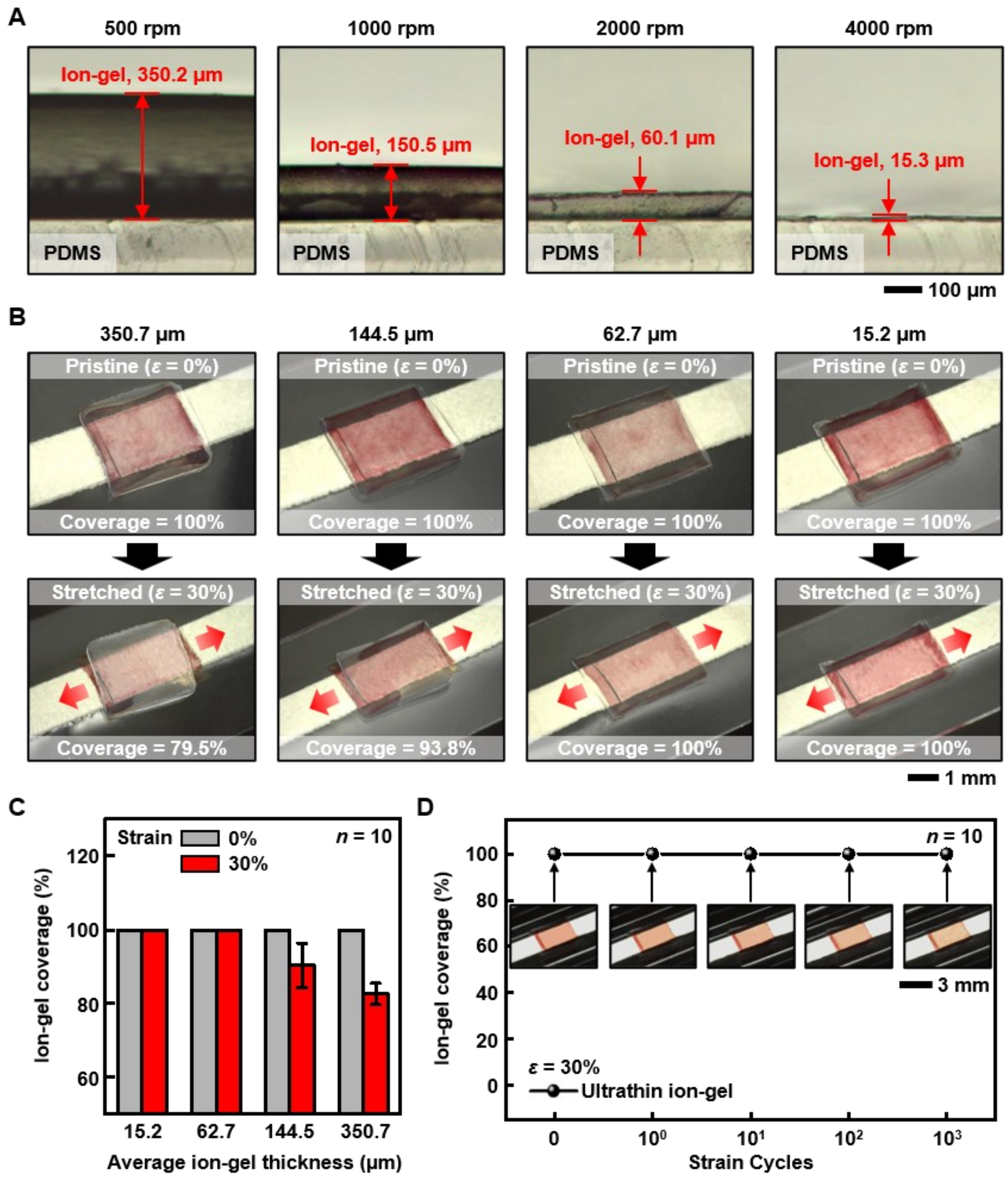

Fig. S10. Effect of ion-gel thickness on deformation stability and channel coverage in the all-soft OECD. (**A**) Optical microscopic cross-sectional images of ion-gel layers with different thicknesses formed on the PDMS substrate. (**B**) Representative optical images of the all-soft OECDs with different average ion-gel thickness, in the pristine state (top) and under 30% tensile strain (bottom). (**C**) Ion-gel coverage of all-soft OECDs as functions of the average ion-gel thickness under tensile strains of 0 and 30%, obtained from 10 devices. (**D**) Quantitative analysis of ultrathin ion-gel coverage on P3HT-NFs/PDMS layer as functions of the number of repeated stain cycles at 30% tensile strain. The ion-gel coverage on the P3HT-NFs/PDMS layer was quantified for each device and tensile deformation. The ultrathin ion-gel with an average thickness of 62.7 μm maintained complete coverage of the P3HT-NFs/PDMS layer under 30% tensile strain and after 1,000 repeated tensile cycles at 30% strain, thereby establishing this thickness as the optimized ion-gel condition for all subsequent devices.

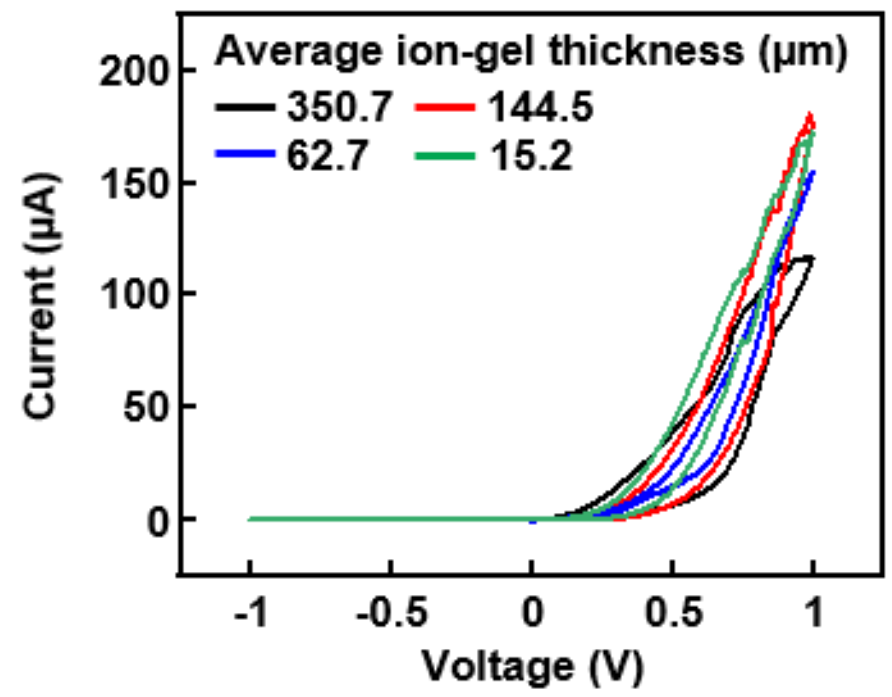

Fig. S11. ***I-V*** **characteristics of the all-soft OECDs fabricated with different ion-gel thickness.**

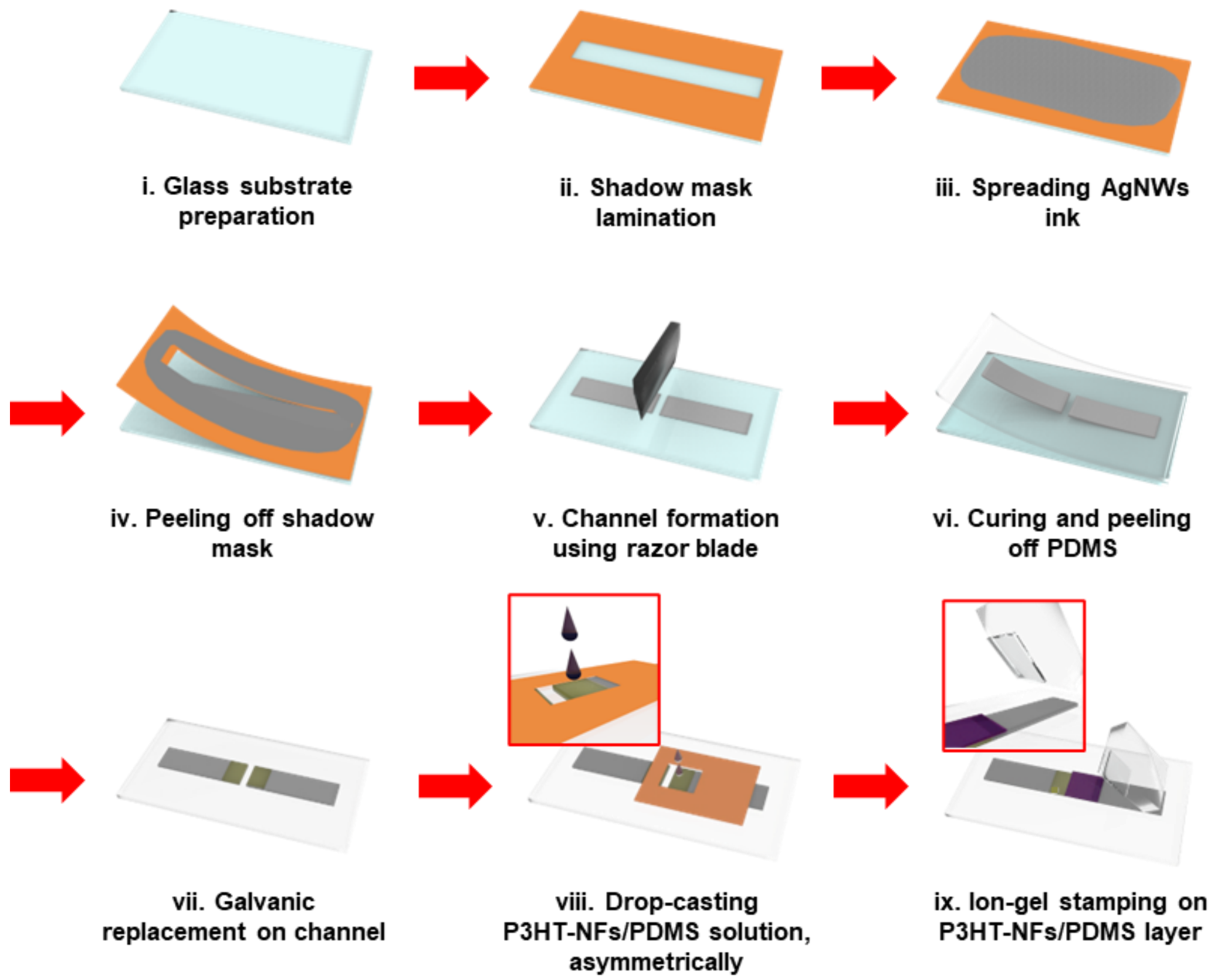

Fig. S12. Schematic illustration of fabrication process for the all-soft OECD.

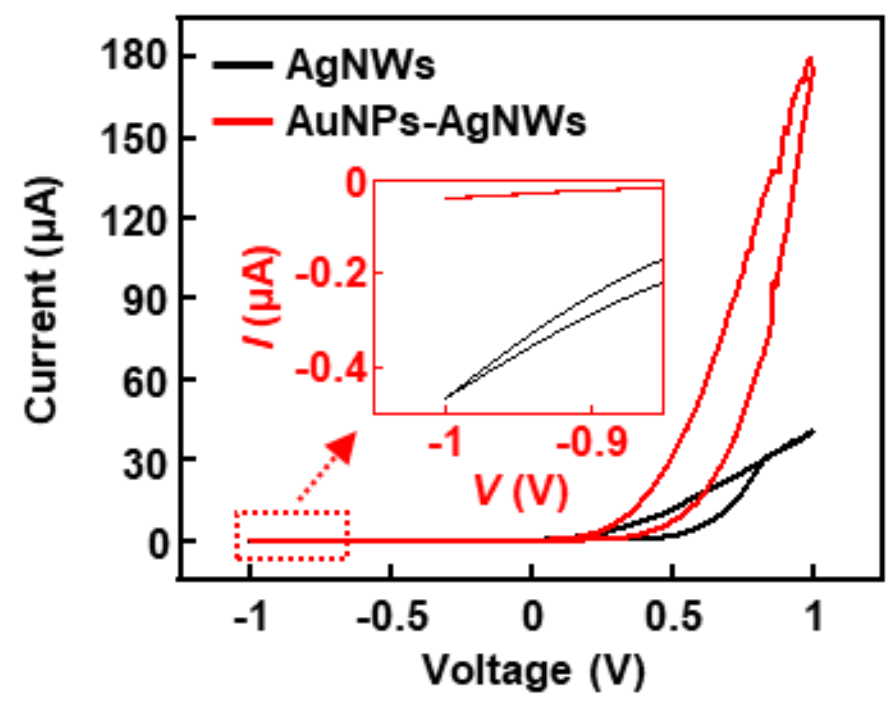

Fig. S13. ***I*-*V* characteristics of the all-soft OECDs fabricated with different electrode materials of AgNWs and AuNPs-AgNWs.**

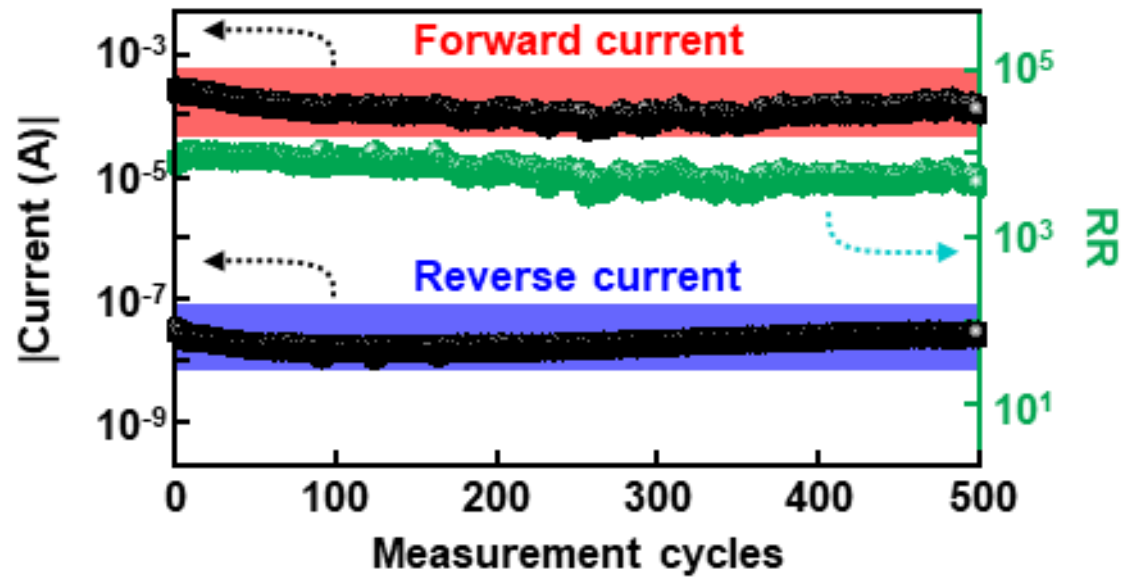

Fig. S14. Extracted I_F, I_R and calculated RR of the all-soft OECD over repeated measurement cycles.

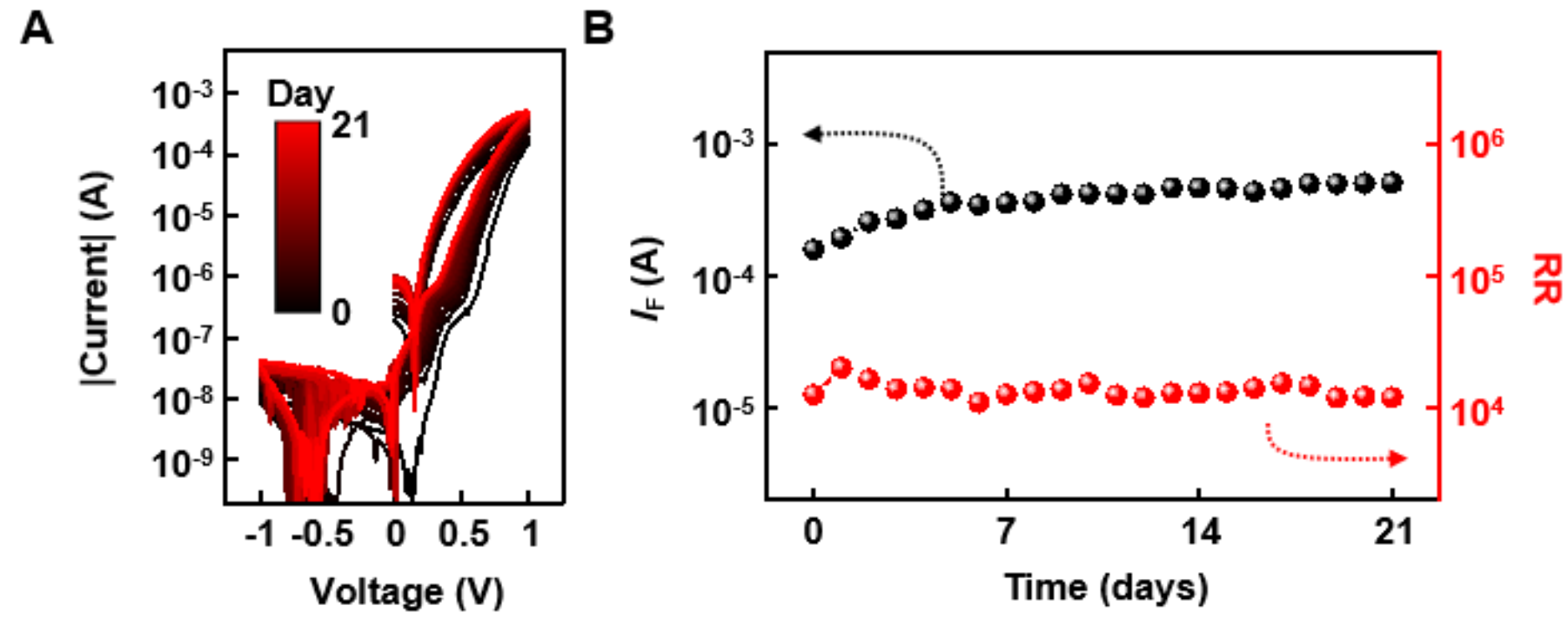

Fig. S15. The ambient stability of the all-soft OECD. (**A**) *I-V* characteristics of the all-soft OECD for 21 days at an ambient environment. (**B**) Extracted I_F and RR of the all-soft OECD as functions of storage times at an ambient environment.

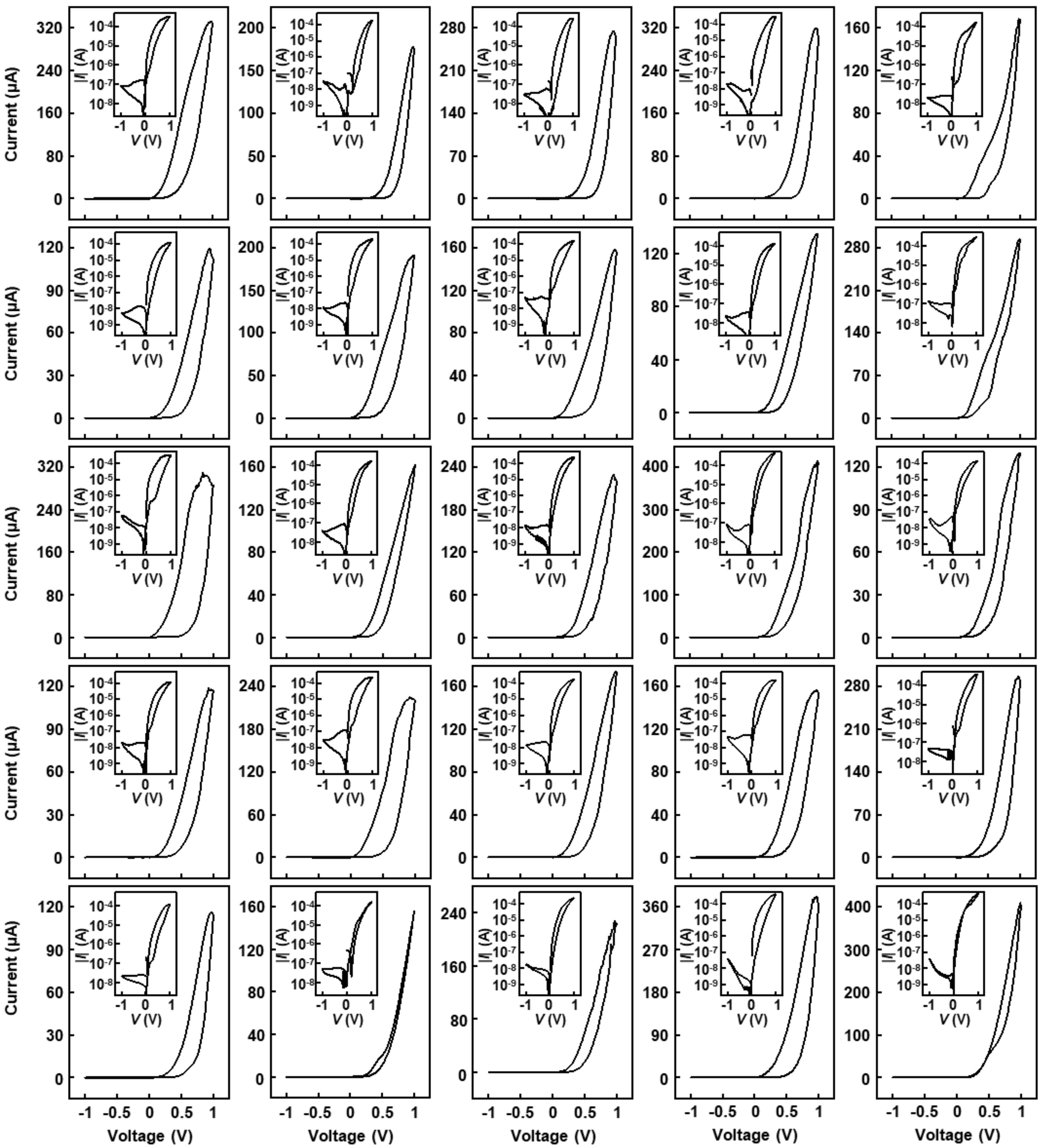

Fig. S16. ***I-V*** **characteristics and corresponding** ***I***$_{\mathrm{F}}$ **and RR of the individual diodes in the 5 × 5 all-soft OECD array.**

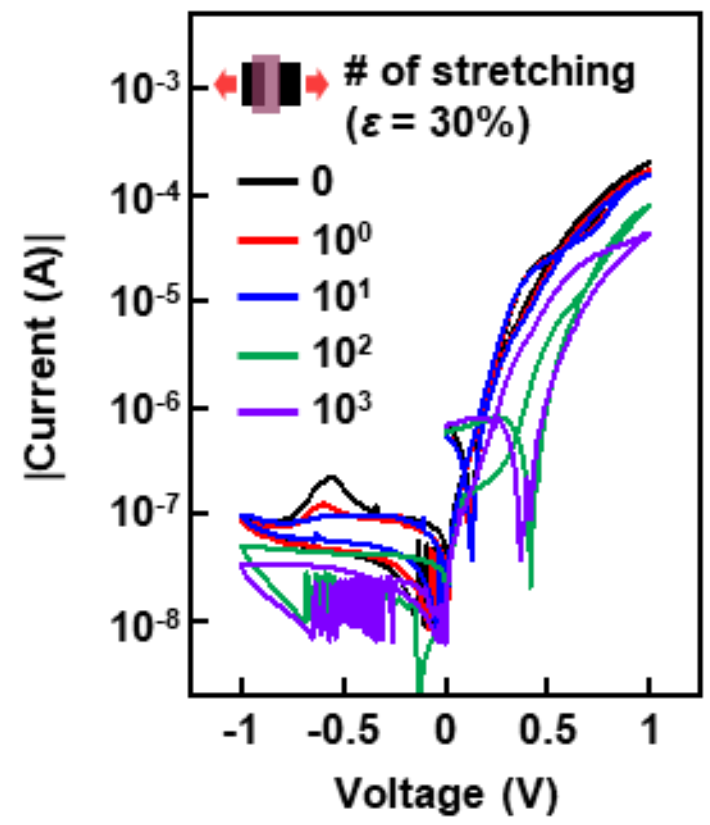

Fig. S17. Representative *I-V* characteristics of the all-soft OECD after repetitive strain cycles at 30% tensile strain parallel to the channel length direction.

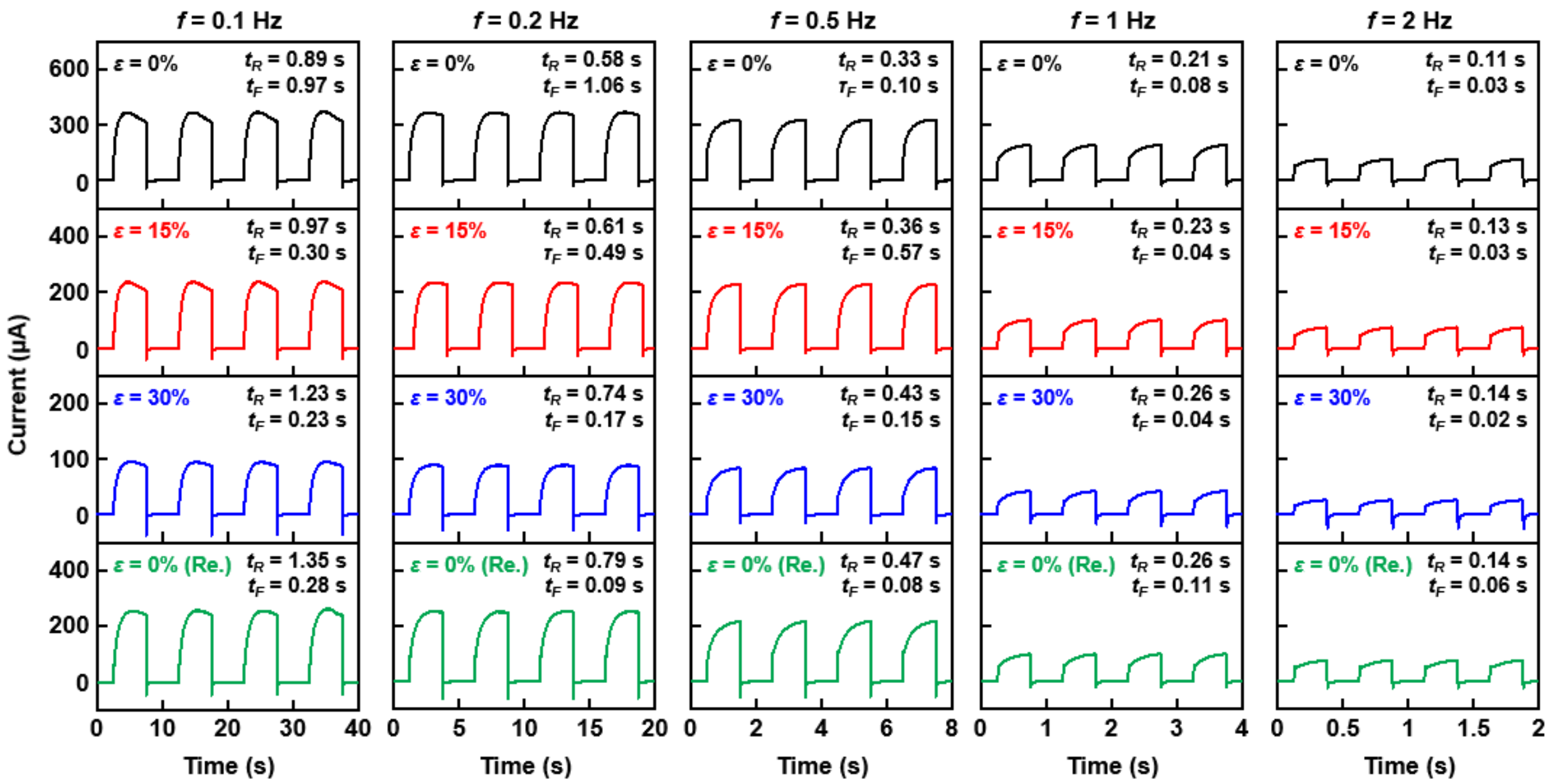

Fig. S18. Dynamic response and corresponding response time (t_R: rise time, t_F: fall time) of the all-soft OECDs under tensile strains of 0, 15 and 30%, and after release, with stain applied parallel to the channel direction measured using a square-wave bias of ±1 V at various frequency (*f*) of 0.1, 0.2, 0.5, 1, and 2 Hz. t_R and t_F represent the turn-on response into the forward-conducting state and the turn-off response into the reverse-bias state and were extracted from the 10-90% current rise and 90-10% current decay, respectively. The reduced current modulation at 1 and 2 Hz is attributed to the shortened bias duration, which limits complete ion-coupled doping/dedoping and RC charging of the soft electrochemical structure.

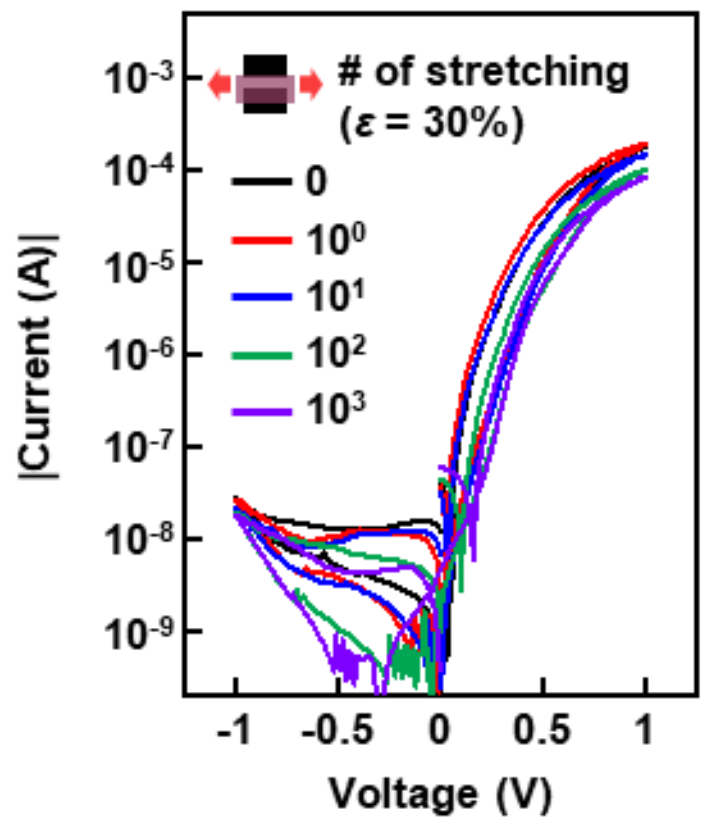

Fig. S19. Representative *I-V* characteristics of the all-soft OECD after repetitive strain cycles at 30% tensile strain perpendicular to the channel length direction.

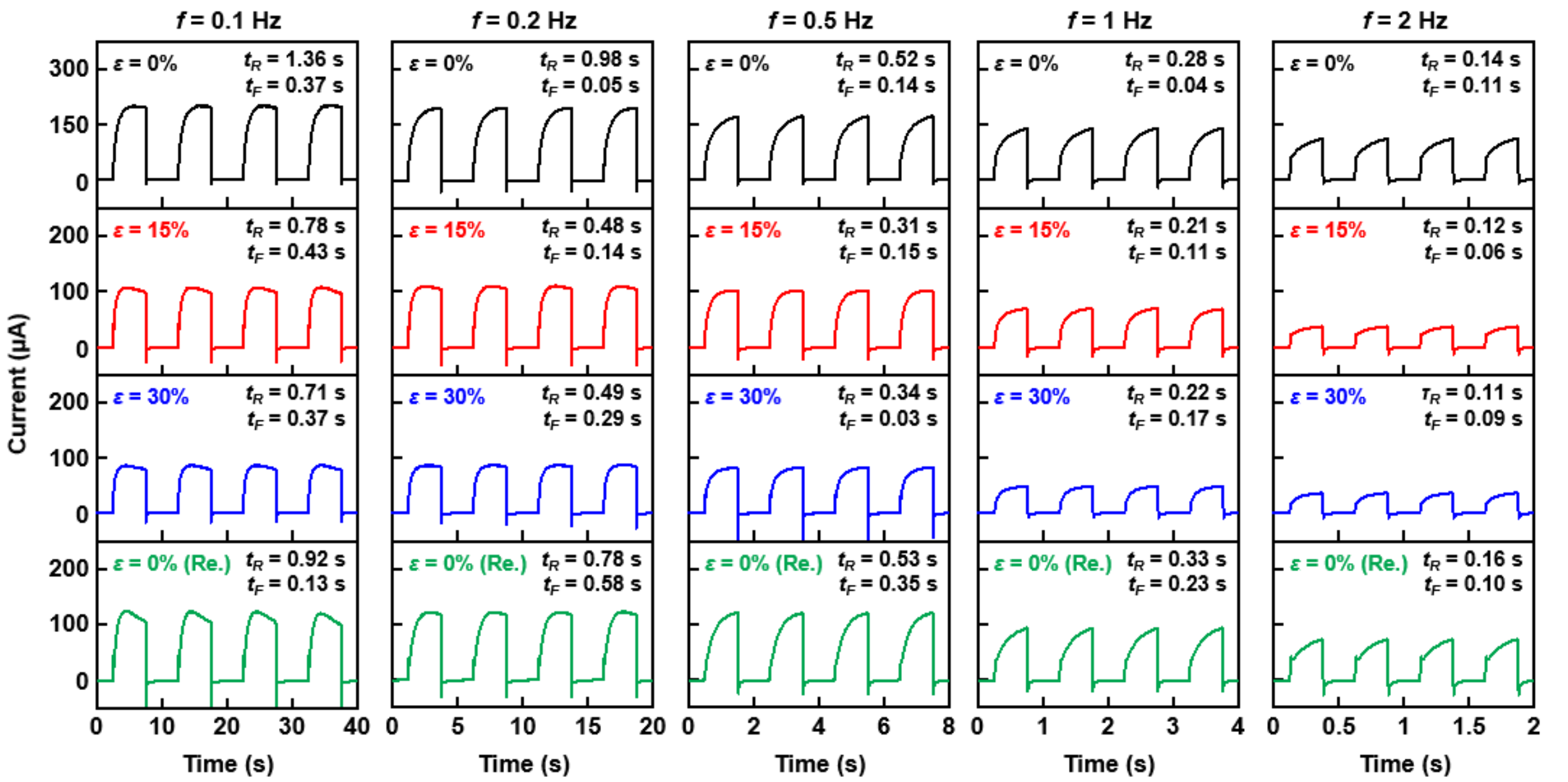

Fig. S20. Dynamic response and corresponding response time (t_R: rise time, t_F: fall time) of the all-soft OECDs under tensile strains of 0, 15 and 30%, and after release, with stain applied perpendicular to the channel direction measured using a square-wave bias of ±1 V at various *f* of 0.1, 0.2, 0.5, 1, and 2 Hz.

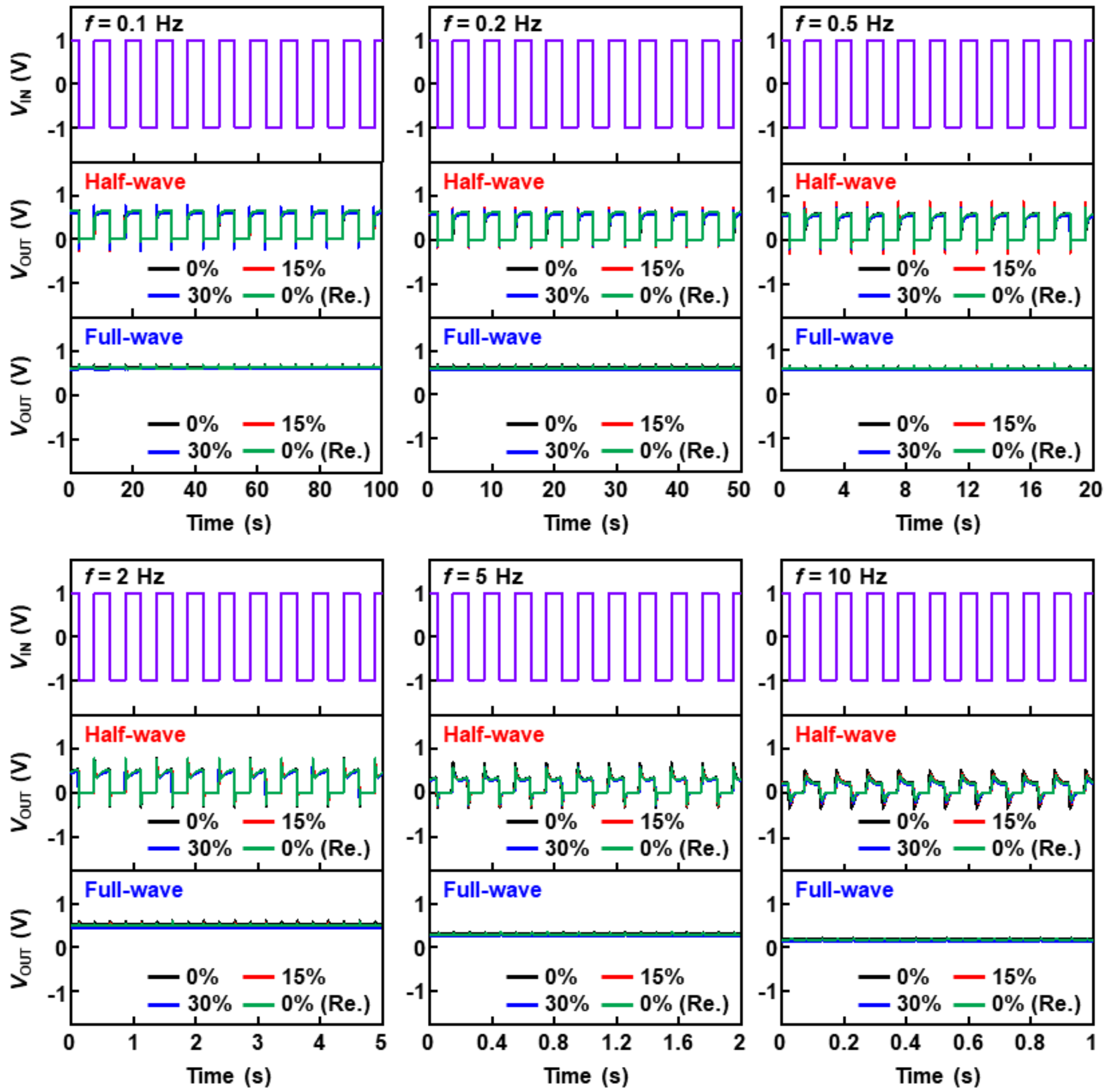

Fig. S21. Representative rectified output voltage (V_{OUT}) of the all-soft half-wave and full-wave rectifiers under tensile strains of 0, 15 and 30%, and after release, measured using a digital input voltage (V_{IN}) of ±1 V at f of 0.1, 0.2, 0.5, 2, 5, and 10 Hz.

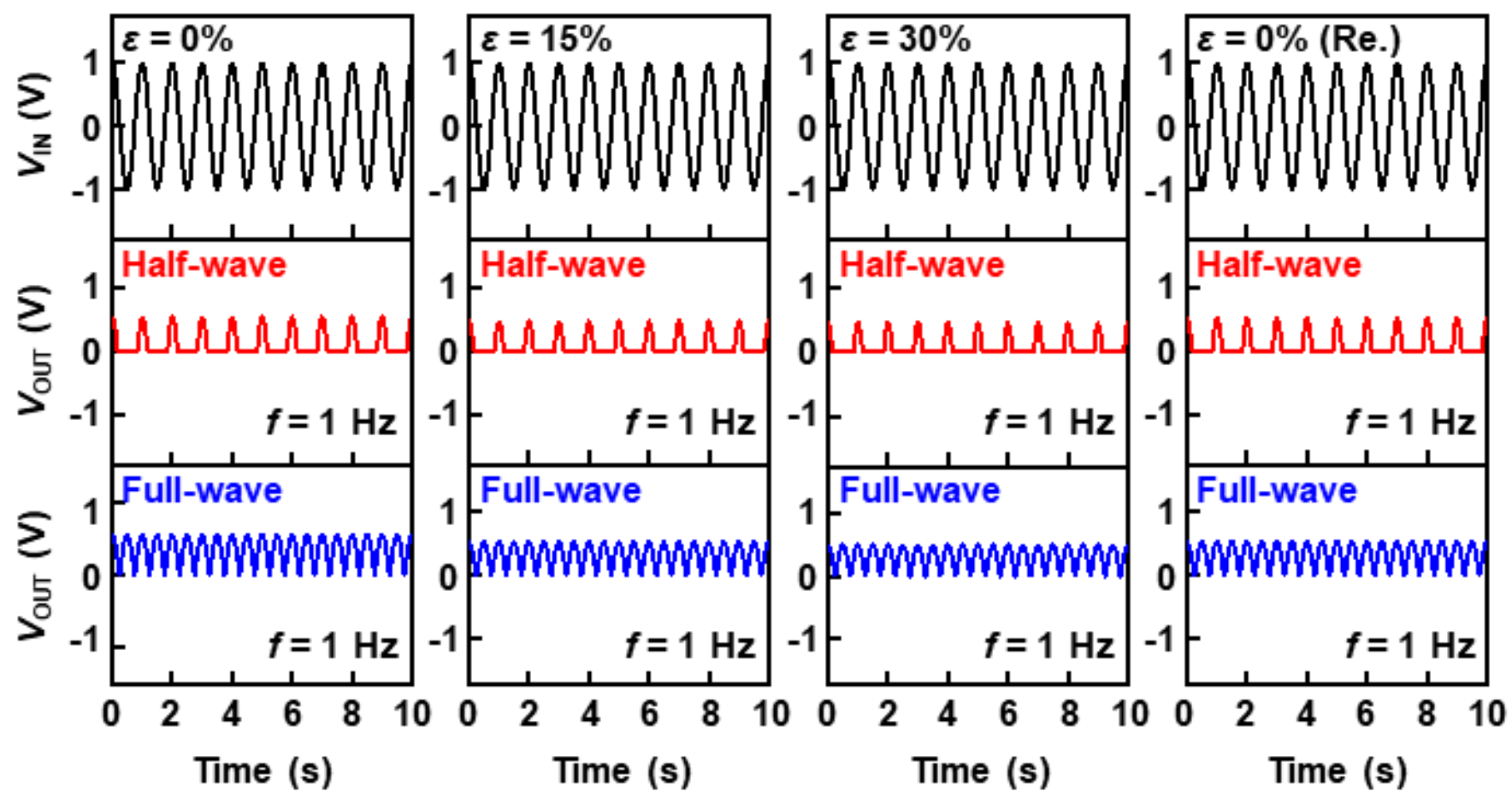

Fig. S22. Representative rectified V_{OUT} signals of the all-soft half-wave and full-wave rectifiers under tensile strains of 0, 15, and 30%, and after release, measured using a sinusoidal V_{IN} of ±1 V at 1 Hz.

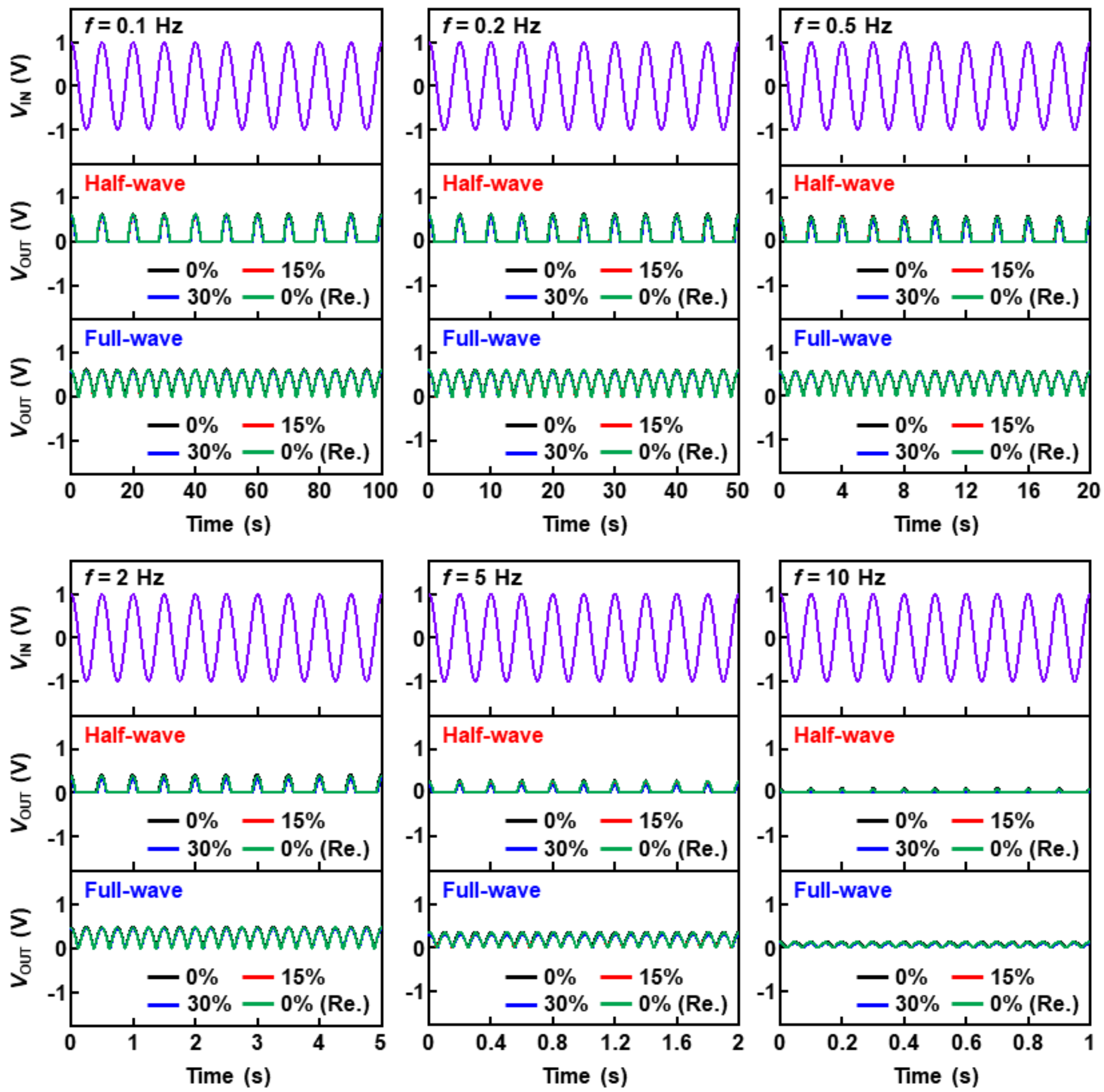

Fig. S23. Representative rectified V_{OUT} of the all-soft half-wave and full-wave rectifiers under tensile strains of 0, 15 and 30%, and after release, measured using a sinusoidal V_{IN} of ±1 V at various f of 0.1, 0.2, 0.5, 2, 5, and 10 Hz.

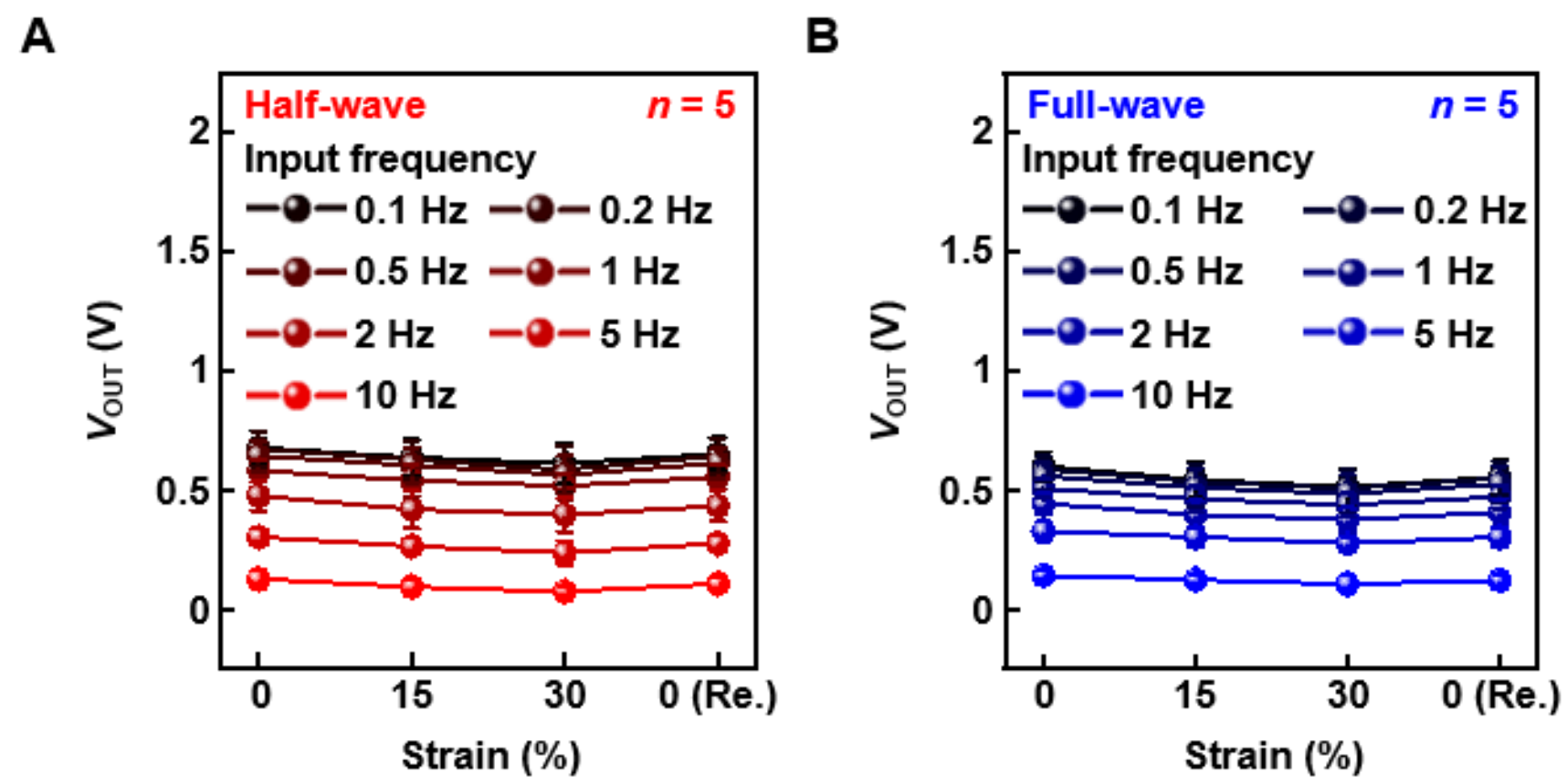

Fig. S24. V_{OUT} of the all-soft rectifiers as a function of tensile strain at different frequencies of the sinusoidal V_{IN} input signal. (**A**) The all-soft half-wave rectifier. (**B**) The all-soft full-wave rectifier.

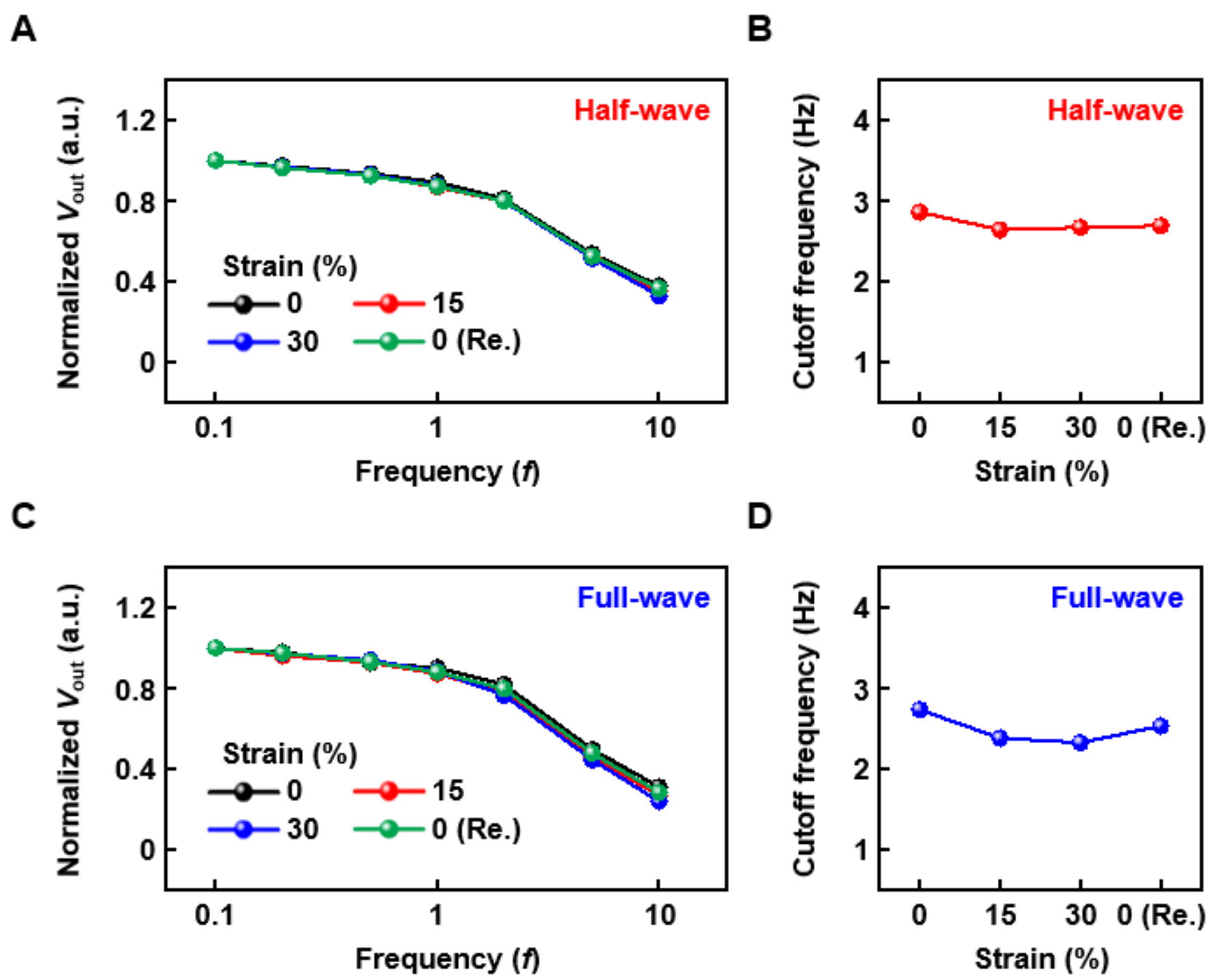

Fig. S25. Frequency-dependent rectification characteristics and extracted -3 dB cutoff frequencies of the all-soft rectifiers. (**A**) Normalized V_{OUT} ($V_{OUT}/V_{OUT,\ 0.1\ Hz}$) of the half-wave rectifier as a function of input frequency under tensile strains of 0, 15, and 30%, and after release. (**B**) Extracted -3 dB cutoff frequencies of the half-wave rectifier under the corresponding strain states. (**C**) Normalized V_{OUT} of the full-wave rectifier as a function of input frequency under tensile strains of 0, 15, and 30%, and after release. (**D**) Extracted -3 dB cutoff frequencies of the full-wave rectifier under the corresponding strain states.

A

All-soft OR gate

$V_{IN,A}$	$V_{IN,B}$	V_{OUT} (0%)	V_{OUT} (15%)	V_{OUT} (30%)	V_{OUT} (0% Re.)
0	0	0	0	0	0
1	0	1	1	1	1
0	1	1	1	1	1
1	1	1	1	1	1

B

All-soft AND gate

$V_{IN,A}$	$V_{IN,B}$	V_{OUT} (0%)	V_{OUT} (15%)	V_{OUT} (30%)	V_{OUT} (0% Re.)
0	0	0	0	0	0
1	0	0	0	0	0
0	1	0	0	0	0
1	1	1	1	1	1

Fig. S26. Truth tables of the all-soft logic gates. (**A**) Truth table of the all-soft OR gate. (**B**) Truth table of the all-soft AND gate.

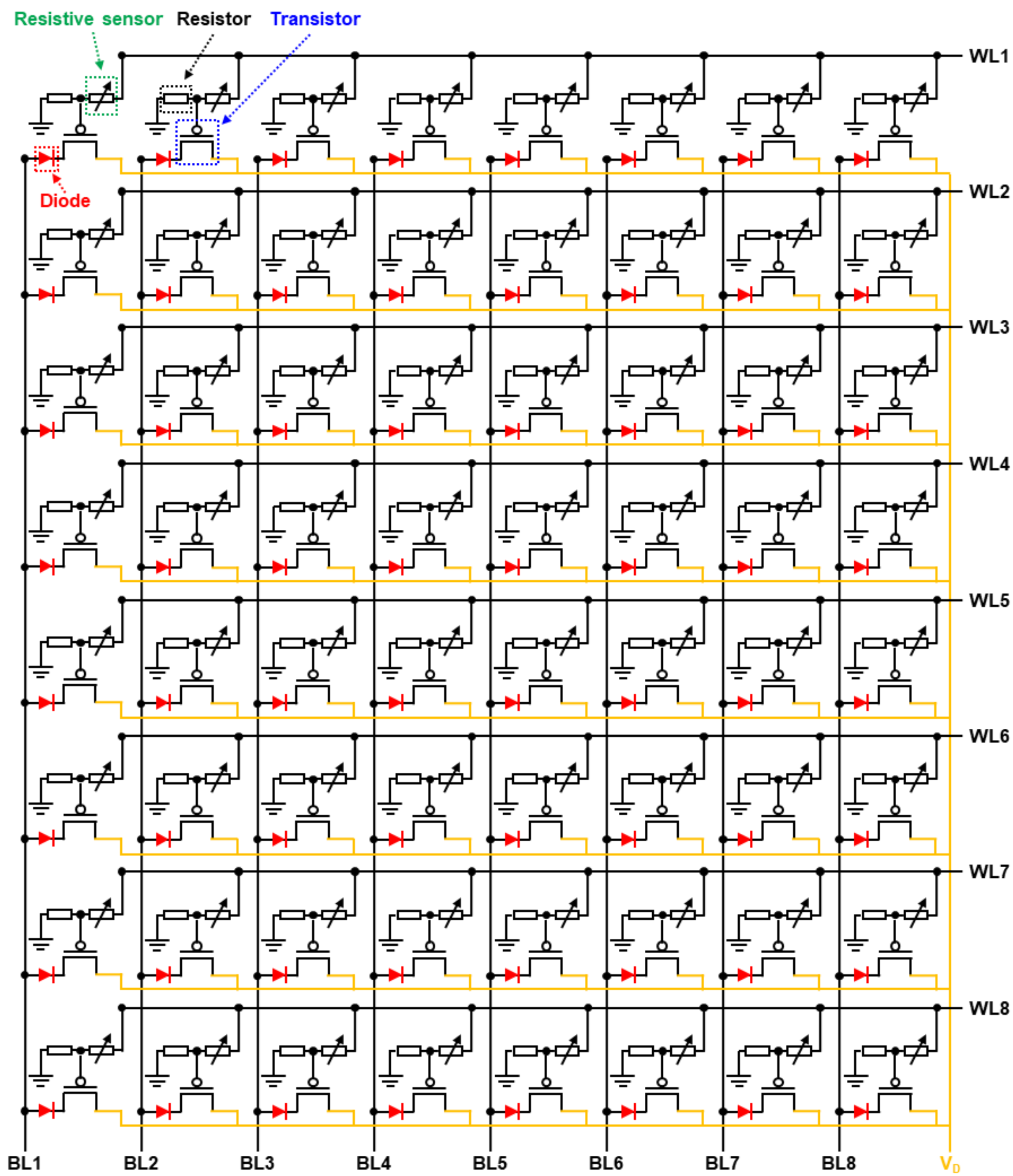

Fig. S27. Representative example of a diode-integrated circuit configuration in sensor arrays consisting of a resistive sensor, a transistor, and a diode for crosstalk suppression between neighboring pixels.

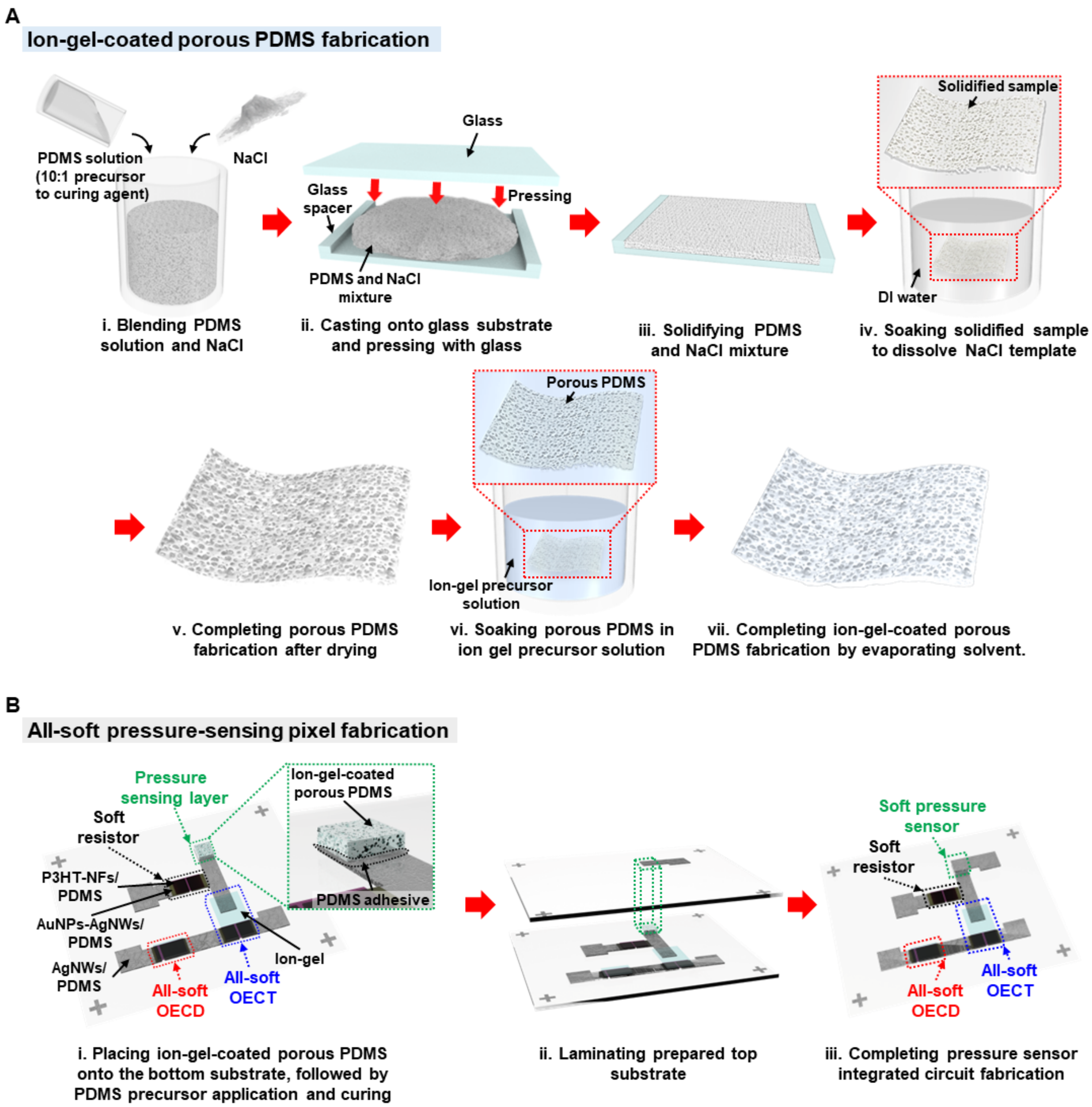

Fig. S28. Schematic fabrication process of the all-soft pressure-sensing pixel. (**A**) Ion-gel-coated porous PDMS fabrication. (**B**) All-soft pressure-sensing pixel fabrication.

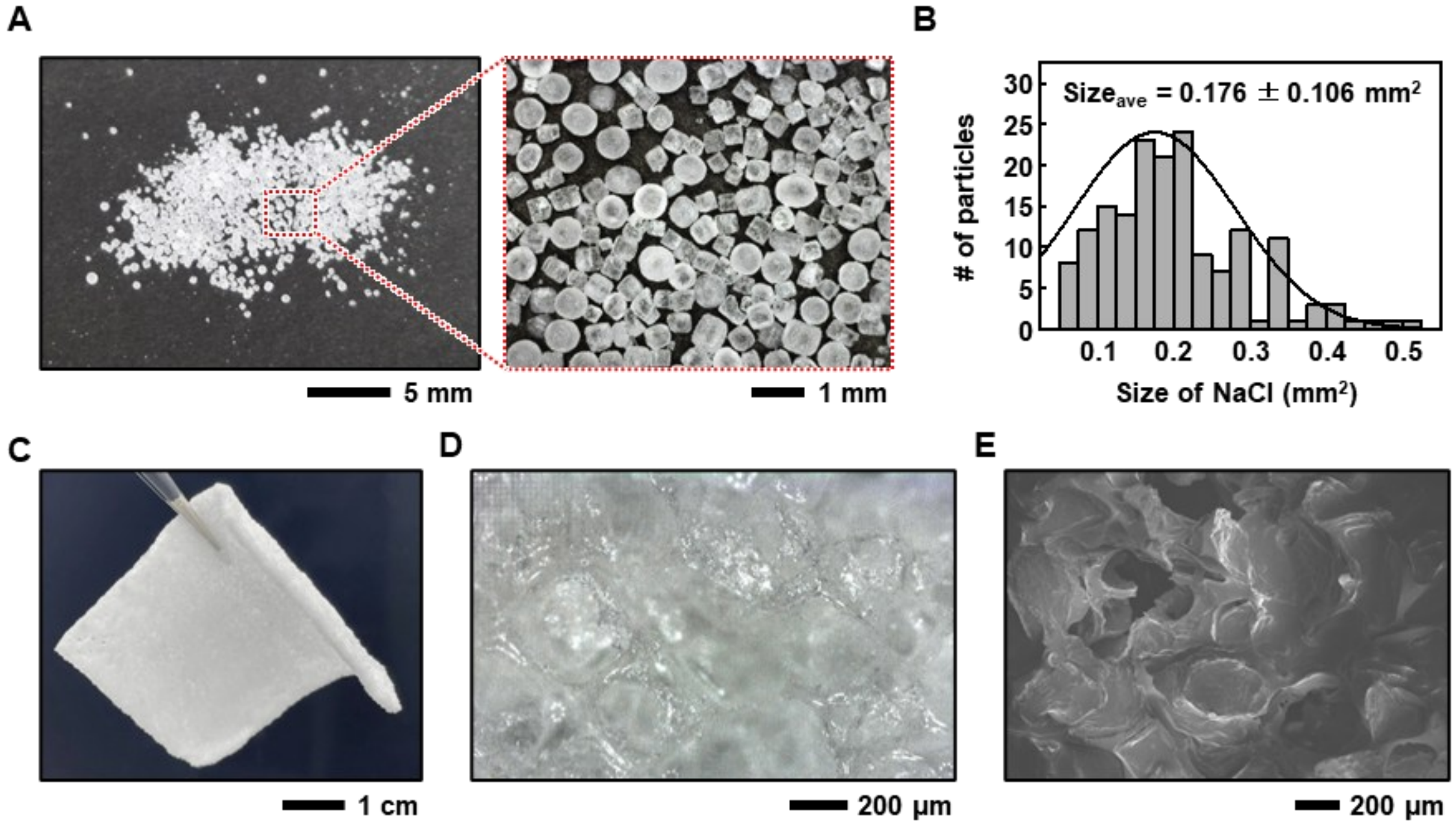

Fig. S29. Morphological characterization of the ion-gel-coated porous PDMS structure used for the soft pressure sensor. (**A**) Optical images of the NaCl particles used as the sacrificial template with a magnified view. (**B**) Statistical distribution of the NaCl template particles. (**C**) Optical image of the ion-gel-coated porous PDMS structure. (**D**) Optical microscopic image of the ion-gel-coated porous PDMS structure. (**E**) Scanning electron microscopy (SEM) image of the ion-gel-coated porous PDMS structure. NaCl was employed as a sacrificial template for the porous PDMS structure because it is soluble in water, whereas cured PDMS is not. Therefore, immersion of the PDMS/NaCl composite in water selectively dissolves the NaCl particles while leaving the PDMS matrix intact, resulting in the formation of a porous PDMS network. Subsequent ion-gel coating on the porous PDMS produced the ion-gel-coated porous PDMS structure. This figure shows the successful formation of the porous PDMS morphology and the final ion-gel-coated porous structure used for the soft pressure sensor.

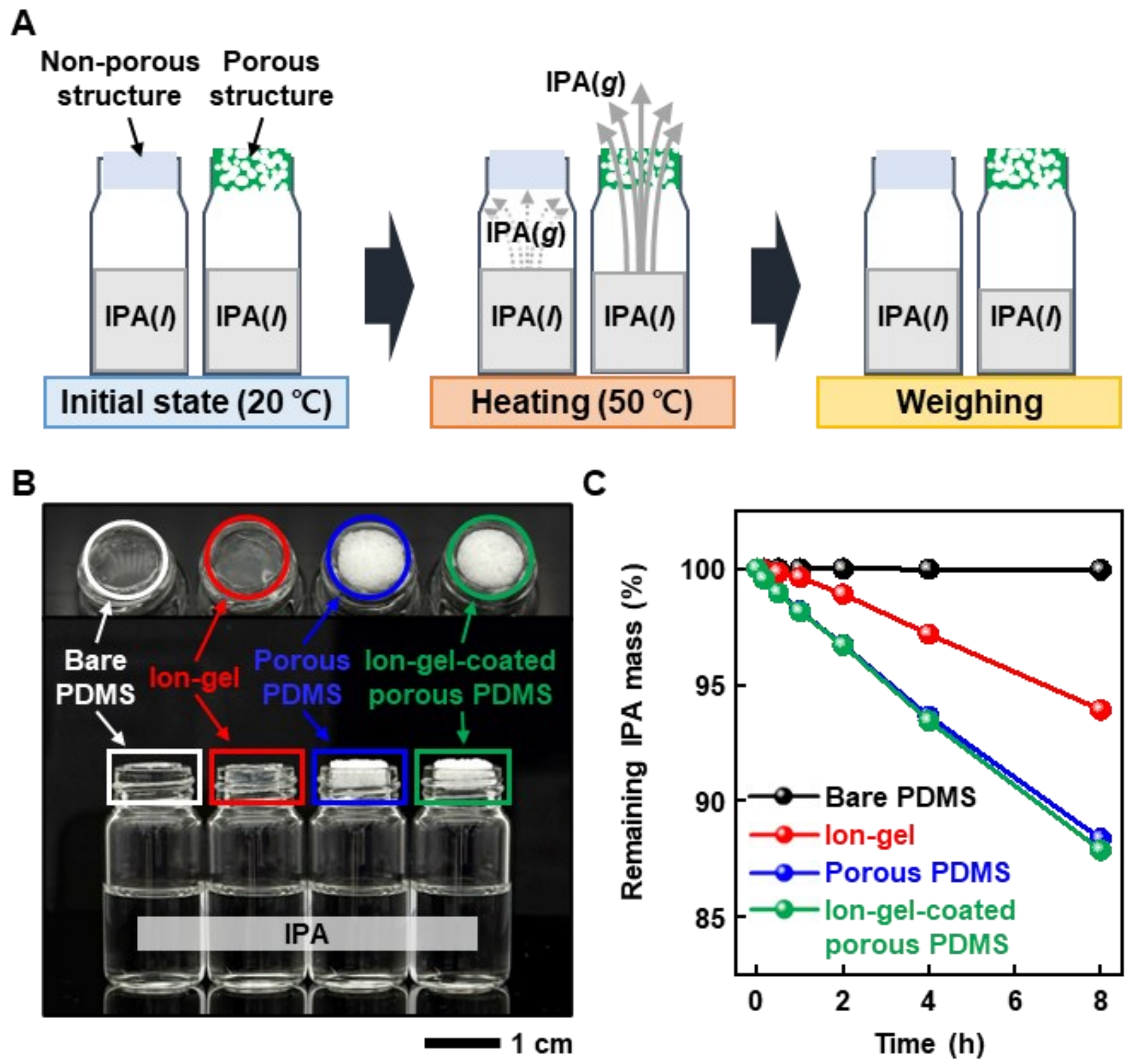

Fig. S30. Evaluation of the porous structure of the ion-gel-coated porous PDMS by solvent-vapor permeation. (**A**) Schematic illustration of the solvent-vapor permeation test. (**B**) Optical images of bare PDMS, ion-gel, porous PDMS, and ion-gel-coated porous PDMS used for the sealing materials (top) and the corresponding isopropyl alcohol (IPA)-filled vials sealed with each material (bottom). (**C**) Remaining IPA mass of the sealed vials as a function of heating time at 50 °C. The ion-gel coating process is not expected to completely fill the pore volume because the porous PDMS was first infiltrated with the ion-gel precursor and then subjected to removal of the excess precursor. Under this process, the precursor is primarily retained on the internal porous PDMS framework and subsequently solidified during solvent evaporation, thereby preserving the interconnected pore volume (*49*). To verify that the porous structure was preserved after ion-gel coating, a solvent-vapor permeation test was performed using IPA-filled vials sealed with bare PDMS, ion-gel, porous PDMS, and ion-gel-coated porous PDMS. Bare PDMS and dense ion-gel suppressed IPA evaporation, whereas porous PDMS showed rapid mass loss because its interconnected pore network allowed IPA vapor to readily escape through the sealing layer. Importantly, the ion-gel-coated porous PDMS exhibited a mass-loss behavior similar to that of porous PDMS, indicating that the porous structure was largely retained after coating.

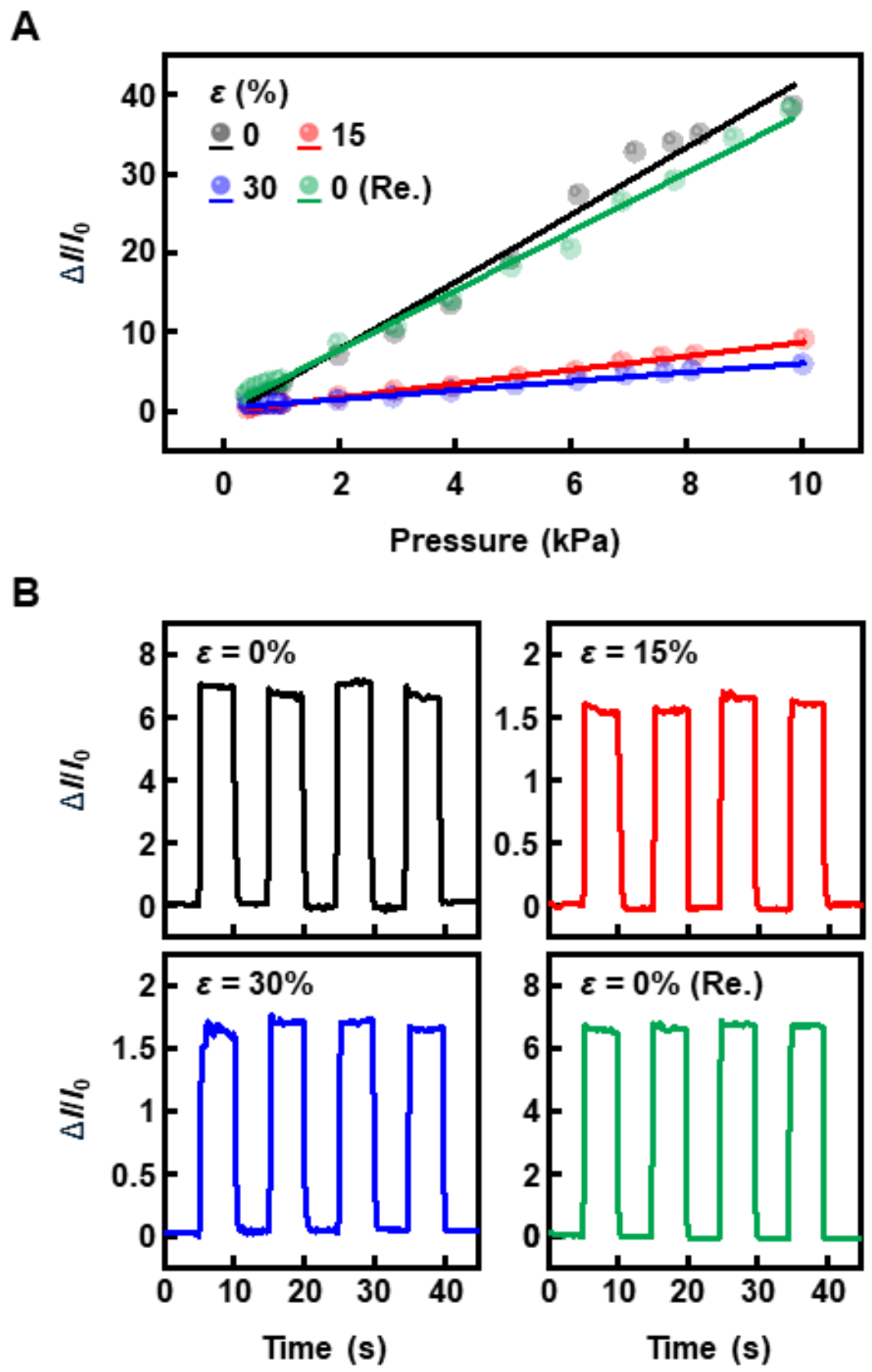

Fig. S31. Pressure-sensing characteristics of the ion-gel-coated porous PDMS pressure sensor. (**A**) Relative current change ($\Delta I/I_0$) of the ion-gel-coated porous PDMS pressure sensor as a function of applied pressure under tensile strains of 0, 15, 30%, and after release, measured at a bias of 1 V. (**B**) Dynamic response of the pressure sensor to a repeated pressure of 1.58 kPa under tensile strains of 0, 15, and 30%, and after release, measured at a bias of 1 V.

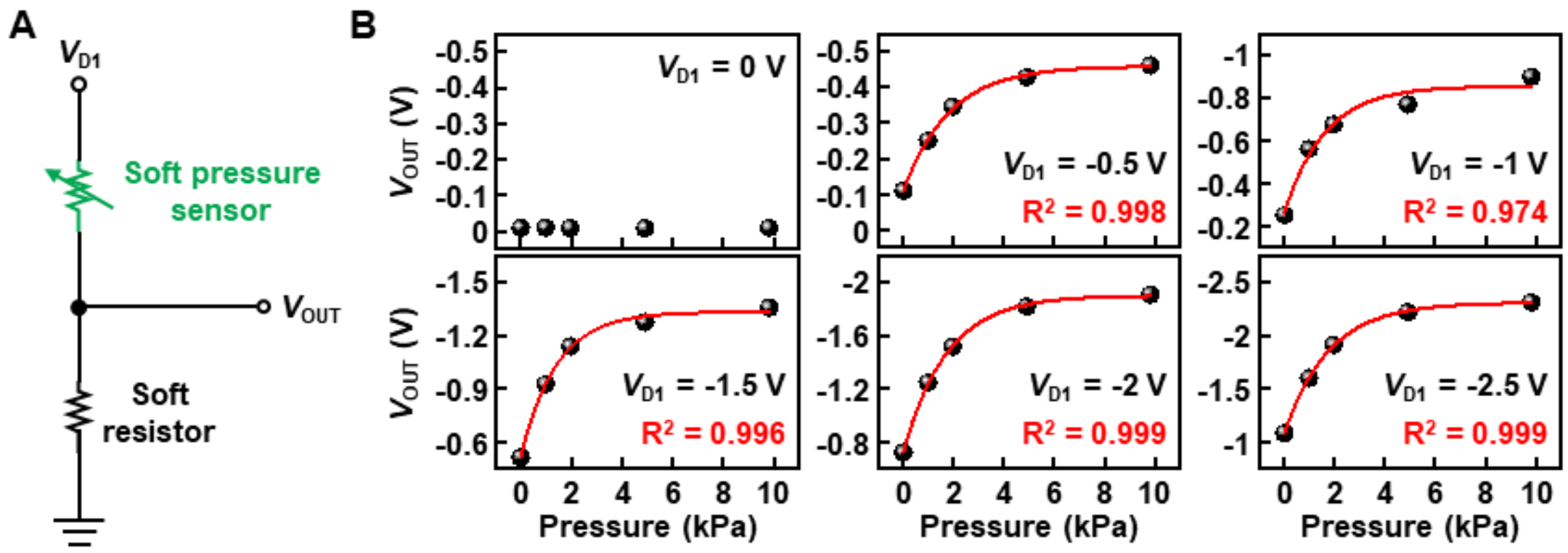

Fig. S32. Output characteristics of the voltage-divider incorporating the ion-gel-coated porous PDMS pressure sensor under different bias conditions. (**A**) Circuit diagram of a voltage divider composed of a soft pressure sensor and the soft resistor. (**B**) V_{OUT} of the voltage-divider as a function of pressure under different applied bias conditions. Symbols represent the experimental data and solid red lines represent empirical fits using a single-exponential function ($V_{OUT} = V_{SAT} + A \cdot \exp(-p/\tau_P)$; V_{SAT}: saturated voltage, A: amplitude, P: applied pressure, τ_P: pressure constant).

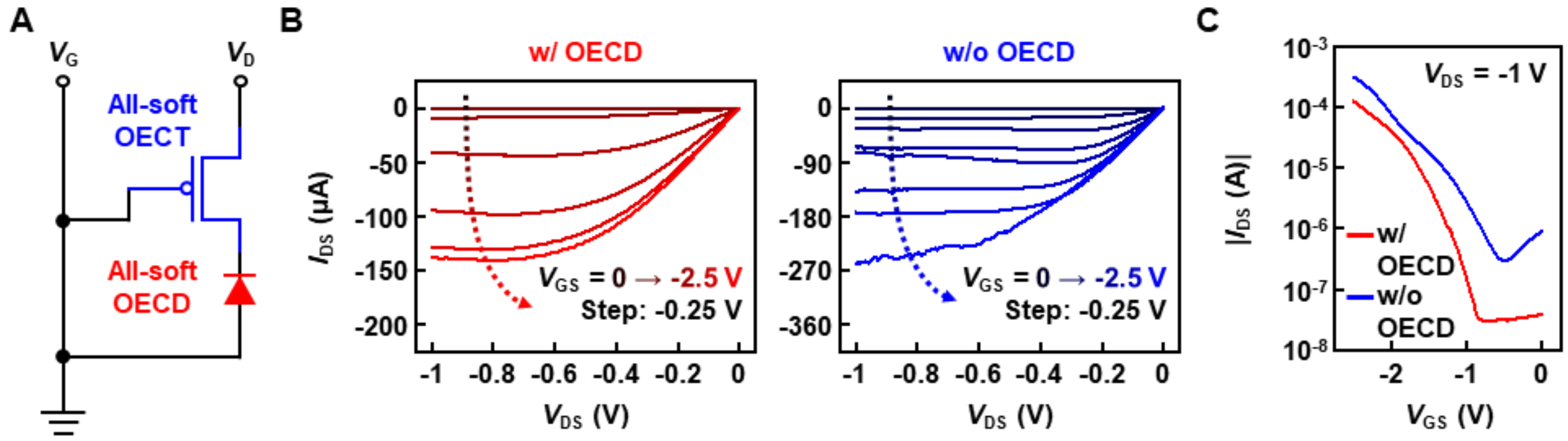

Fig. S33. Comparison of electrical characteristics of the all-soft OECT and the all-soft OECT integrated with the all-soft OECD. (**A**) Circuit diagram of all-soft OECT integrated with the OECD. (**B**) Output characteristics of the all-soft OECT with (left) and without (right) the all-soft OECD. (**C**) Transfer characteristics of all-soft OECT with and without the all-soft OECD.

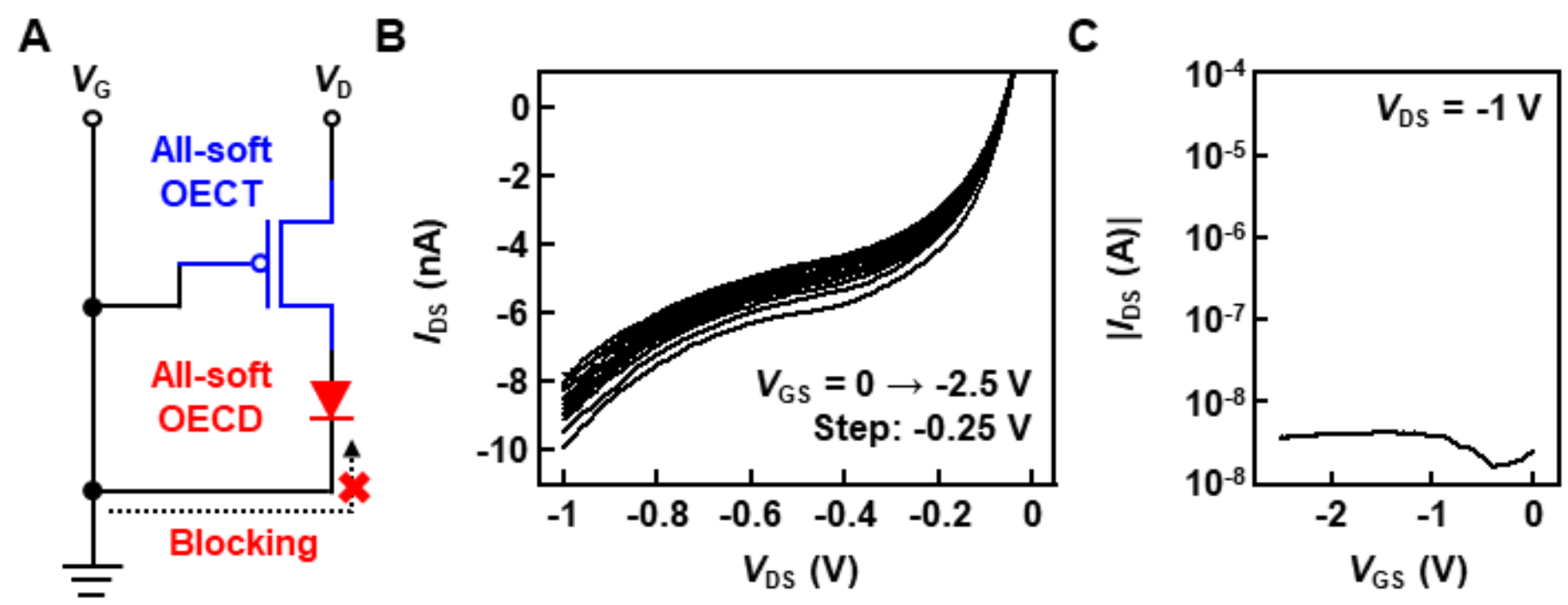

Fig. S34. The electrical characterization of the all-soft OECT integrated with a reverse-connected all-soft OECD. (**A**) Circuit diagram of the all-soft OECT with the reverse-connected all-soft OECD. (**B and C**) Output (B) and transfer (C) characteristics of the all-soft OECT with the reverse-connected all-soft OECD.

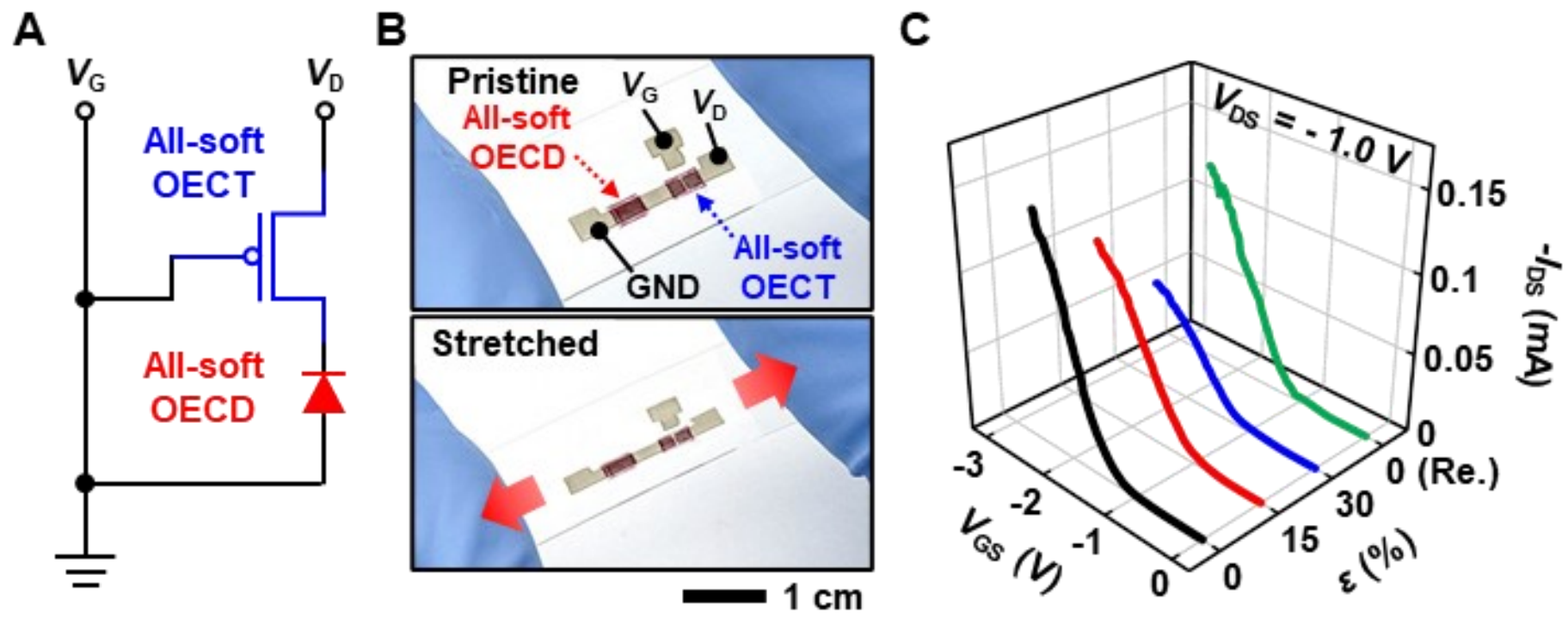

Fig. S35. Electrical characterization of the all-soft OECT integrated with the all-soft OECD under tensile strain. (**A**) Circuit diagram of the all-soft OECT with the OECD. (**B**) Optical images of the all-soft OECT with the OECD in the pristine and stretched states. (**C**) Transfer characteristics of the all-soft OECT with the OECD under tensile strains of 0, 15, and 30%, and after release.

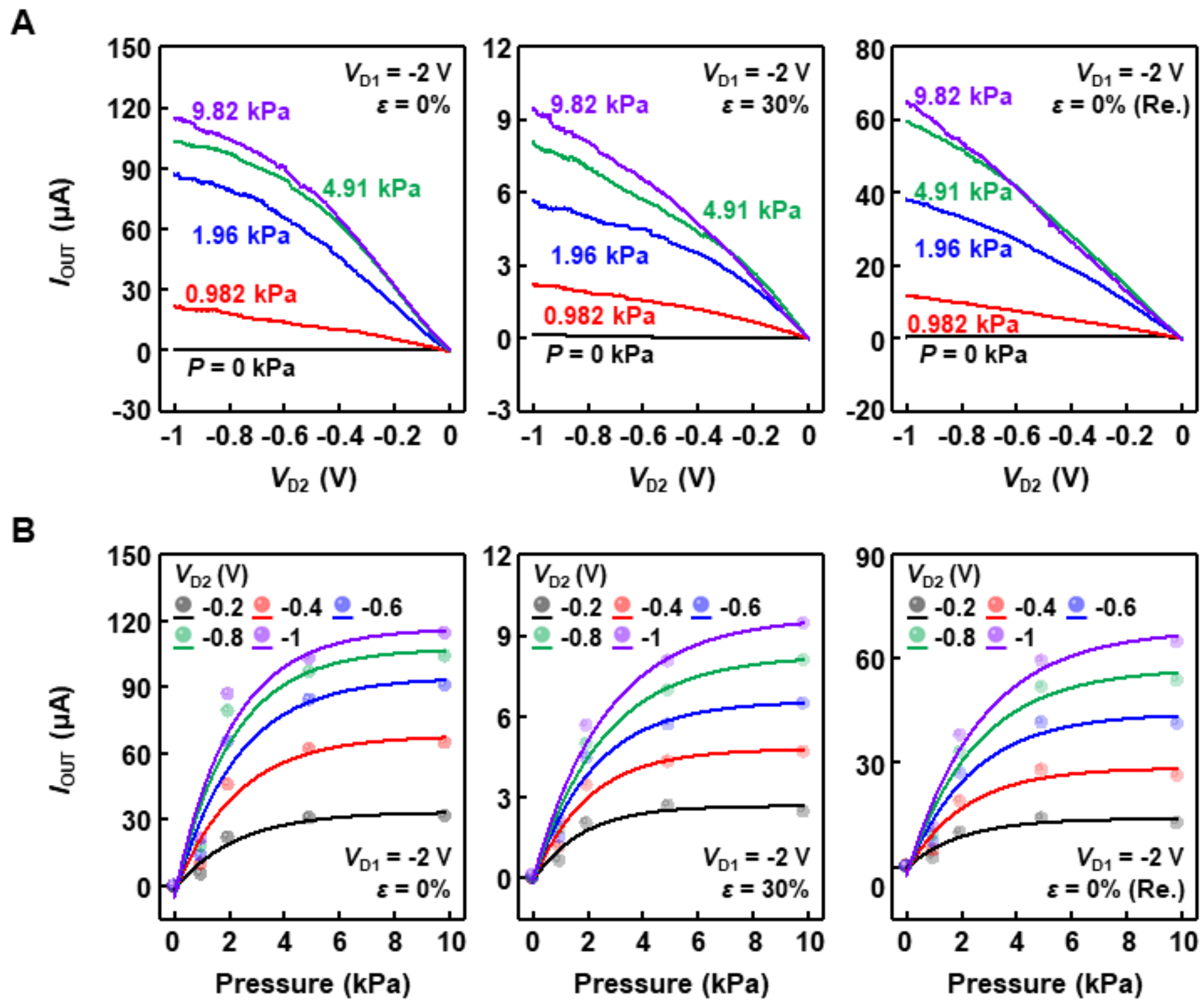

Fig. S36. Electrical characteristics of the all-soft pressure-sensing pixel integrating the OECD, OECT, and pressure sensor. (**A**) I_{OUT} as a function of V_{D2} at different applied pressure (0, 0.982, 1.96, 4.91, and 9.82 kPa) for the all-soft pressure-sensing pixel under tensile strains of 0 and 30%, and after release, measured at a fixed V_{D1} of -2 V. (**B**) Extracted calibration curves of I_{OUT} as a function of pressure at different V_{D2} (-0.2, -0.4, -0.6, -0.8, and -1 V) under the same mechanical conditions. Symbols represent the extracted I_{OUT} and solid lines represent empirical fits using a single-exponential function ($I_{OUT} = I_{OUT} + A\cdot\exp(p/\tau_P)$; I_{SAT}: saturated current).

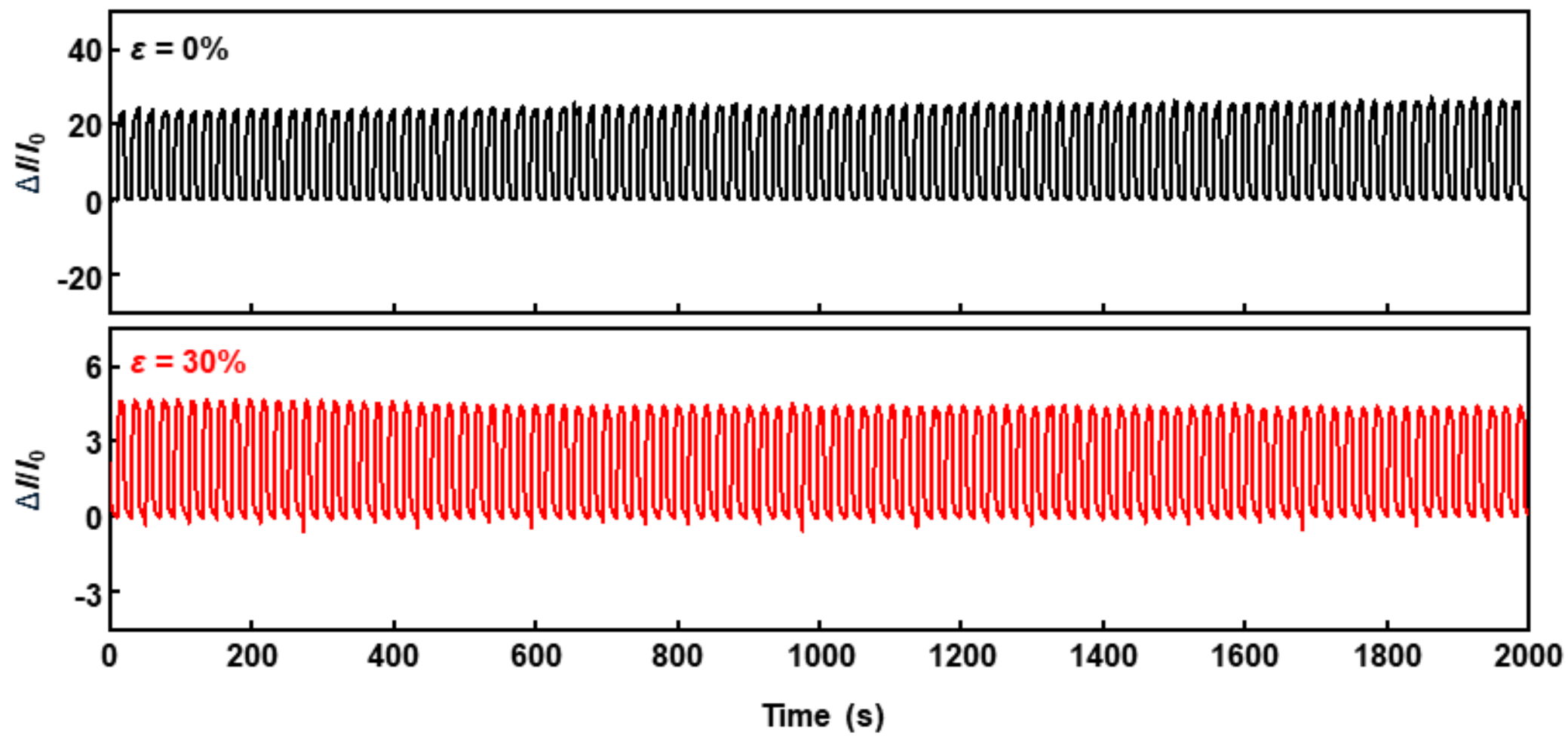

Fig. S37. Dynamic pressure response of the all-soft pressure-sensing pixel under cyclic pressure of 1.58 kPa during 100 pressure cycles at 0 and 30% tensile strain, measured at V_{D1} and V_{D2} of -2 and -1 V, respectively.

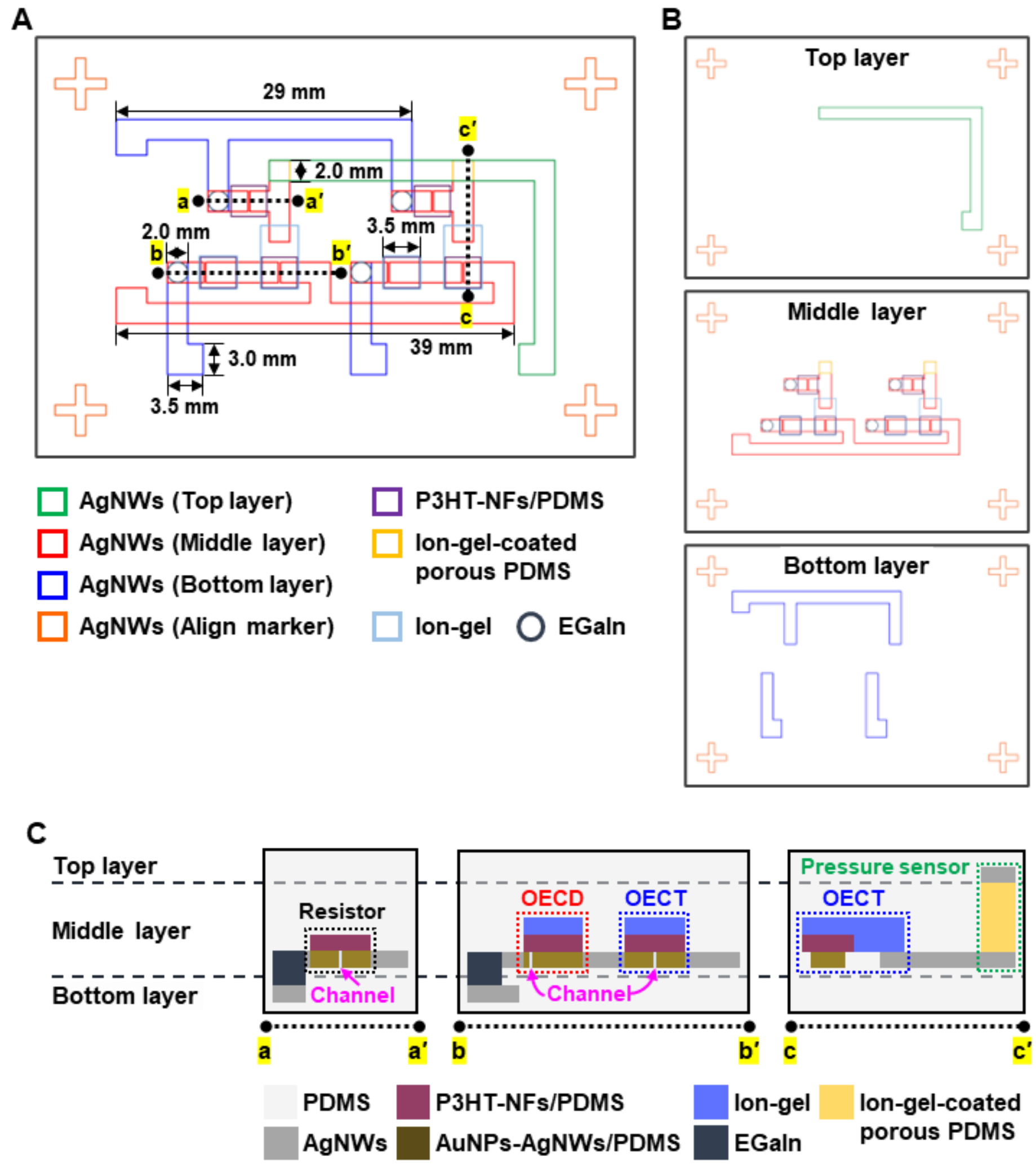

Fig. S38. Detailed structural design of the two-pixel configuration for crosstalk evaluation in the all-soft pressure-sensing array. (**A and B**) Overlaid (A) and layer-by-layer (B) layouts of the two-pixel configuration with the geometry dimensions and device components. (**C**) Schematic illustration of cross-sectional views along the a-a'. b-b', and c-c' lines showing the stacked structure of the integrated sensing pixels.

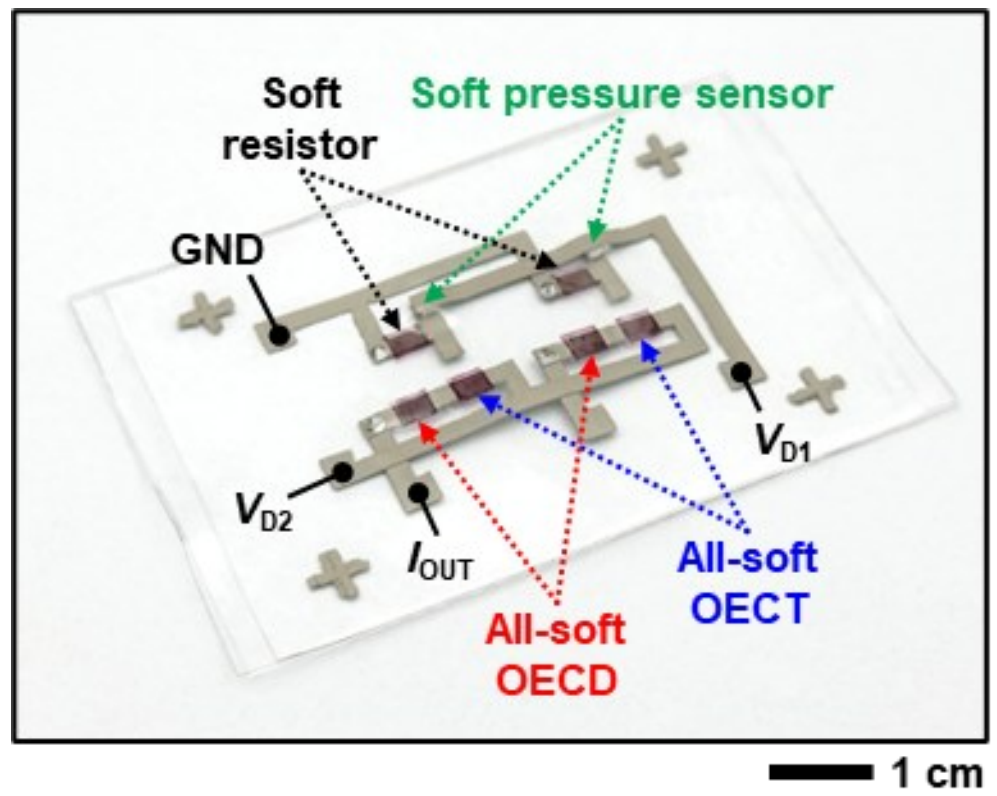

Fig. S39. Optical image of the two-pixel pressure-sensing device.

REFERENCES

1. D. Son, J. Kang, O. Vardoulis, Y. Kim, N. Matsuhisa, J. Y. Oh, J. W. F. To, J. Mun, T. Katsumata, Y. Liu, A. F. McGuire, M. Krason, F. Molina-Lopez, J. Ham, U. Kraft, Y. Lee, Y. Yun, J. B.-H. Tok, Z. Bao, An integrated self-healable electronic skin system fabricated via dynamic reconstruction of a nanostructured conducting network. *Nat. Nanotechnol.* **13**, 1057–1065 (2018).

2. F. Ershad, A. Thukral, J. Yue, P. Comeaux, Y. Lu, H. Shim, K. Sim, N.-I. Kim, Z. Rao, R. Guevara, L. Contreras, F. Pan, Y. Zhang, Y.-S. Guan, P. Yang, X. Wang, P. Wang, X. Wu, C. Yu, Ultra-conformal drawn-on-skin electronics for multifunctional motion artifact-free sensing and point-of-care treatment. *Nat. Commun.* **11**, 3823 (2020).

3. S. Na, J. Yeom, Y. Chang, Y. Kwon, C. Park, Y. E. Shin, Y. J. Park, H. Ko, Stretchable skin hydration sensor based on hygroscopic and ion conductive polymer composites. *Chem. Eng. J.* **455**, 140957 (2023).

4. Y. Lee, X. Tian, J. Park, D. H. Nam, Z. Wu, H. Choi, J. Kim, D.-W. Park, K. Zhou, S. W. Lee, T. A. Tabish, X. Cheng, S. Emaminejad, T.-W. Lee, H. Kim, A. Khademhosseini, Y. Zhu, Rapidly self-healing electronic skin for machine learning-assisted physiological and movement evaluation. *Sci. Adv.* **11**, eads1301 (2025).

5. H. U. Chung, B. H. Kim, J. Y. Lee, J. Lee, Z. Xie, E. M. Ibler, K. Lee, A. Banks, J. Y. Jeong, J. Kim, C. Ogle, D. Grande, Y. Yu, H. Jang, P. Assem, D. Ryu, J. W. Kwak, M. Namkoong, J. B. Park, Y. Lee, D. H. Kim, A. Ryu, J. Jeong, K. You, B. Ji, Z. Liu, Q. Huo, X. Feng, Y. Deng, Y. Xu, K.-I. Jang, J. Kim, Y. Zhang, R. Ghaffari, C. M. Rand, M. Schau, A. Hamvas, D. E. Weese-Mayer, Y. Huang, S. M. Lee, C. H. Lee, N. R. Shanbhag, A. S. Paller, S. Xu, J. A. Rogers, Binodal, wireless epidermal electronic systems with in-sensor analytics for neonatal intensive care. *Science* **363**, eaau0780 (2019).

6. Y. Lee, J. W. Chung, G. H. Lee, H. Kang, J.-Y. Kim, C. Bae, H. Yoo, S. Jeong, H. Cho, S.-G. Kang, J. Y. Jung, D.-W. Lee, S. Gam, S. G. Hahm, Y. Kuzumoto, S. J. Kim, Z. Bao, Y. Hong, Y. Yun, S. Kim, Standalone real-time health monitoring patch based on a stretchable organic optoelectronic system. *Sci. Adv.* **7**, eabg9180 (2021).

7. C. H. Xu, Y. Song, J. R. Sempionatto, S. A. Solomon, Y. Yu, H. Y. Y. Nyein, R. Y. Tay, J. H. Li, W. Z. Heng, J. H. Min, A. Lao, T. K. Hsiai, J. A. Sumner, W. Gao, A physicochemical-sensing electronic skin for stress response monitoring. *Nat. Electron.* **7**, 168–179 (2024).

8. L. Li, X. Ye, Z. Ji, M. Zheng, S. Lin, M. Wang, J. Yang, P. Zhou, Z. Zhang, B. Wang, H. Wang, Y. Wang, Paintable, Fast Gelation, Highly Adhesive Hydrogels for High-fidelity Electrophysiological Monitoring Wirelessly. *Small* **21**, e2407996 (2025).

9. K. Sim, F. Ershad, Y. Zhang, P. Yang, H. Shim, Z. Rao, Y. Lu, A. Thukral, A. Elgalad, Y. Xi, B. Tian, D. A. Taylor, C. Yu, An epicardial bioelectronic patch made from soft rubbery materials and capable of spatiotemporal mapping of electrophysiological activity. *Nat. Electron.* **3**, 775–784 (2020).

10. S. Wang, Y. Nie, H. Zhu, Y. Xu, S. Cao, J. Zhang, Y. Li, J. Wang, X. Ning, D. Kong, Intrinsically stretchable electronics with ultrahigh deformability to monitor dynamically moving organs. *Sci. Adv.* **8**, eabl5511 (2022).

11. S. Jin, H. Choi, D. Seong, C.-L. You, J.-S. Kang, S. Rho, W. B. Lee, D. Son, M. Shin, Injectable tissue prosthesis for instantaneous closed-loop rehabilitation. *Nature* **623**, 58–65 (2023).

12. K. H. Jung, J. Hyun, M. W. Jeong, Y. S. Koo, T. U. Nam, N. T. P. Vo, J. Park, J. An, D.-H. Lee, S.-W. Kim, J. Yang, J.-K. Yoon, S. H. Bhang, J. Y. Oh, A biocompatible elastomeric organic transistor for implantable electronics. *Nat. Electron.* **8**, 831–843 (2025).

13. H. Shim, K. Sim, F. Ershad, P. Yang, A. Thukral, Z. Rao, H.-J. Kim, Y. Liu, X. Wang, G. Gu, L. Gao, X. Wang, Y. Chai, C. Yu, Stretchable elastic synaptic transistors for neurologically integrated soft engineering systems. *Sci. Adv.* **5**, eaax4961 (2019).

14. G. Jin, Y. Sun, J. Geng, X. Yuan, T. Chen, H. Liu, F. Wang, L. Sun, Bioinspired soft caterpillar robot with ultra-stretchable bionic sensors based on functional liquid metal. *Nano Energy* **84**, 105896 (2021).

15. H. Bai, Y. S. Kim, R. F. Shepherd, Autonomous self-healing optical sensors for damage intelligent soft-bodied systems. *Sci. Adv.* **8**, eabq2104 (2022).

16. J. Lee, H. Park, S. Kim, C. Liu, Z. Li, K. Sim, Irreproducible SEBS wrinkling based on spin evaporation enabling identifiable artificial finger pad electronics. *Nat. Commun.* **16**, 2225 (2025).

17. H. Shim, K. Sim, B. Wang, Y. Zhang, S. Patel, S. Jang, T. J. Marks, A. Facchetti, C. Yu, Elastic integrated electronics based on a stretchable n-type elastomer-semiconductor-elastomer stack. *Nat. Electron.* **6**, 349–359 (2023).

18. D. Zhong, C. Wu, Y. Jiang, Y. Yuan, M.-G. Kim, Y. Nishio, C.-C. Shih, W. Wang, J.-C. Lai, X. Ji, T. Z. Gao, Y.-X. Wang, C. Xu, Y. Zheng, Z. Yu, H. Gong, N. Matsuhisa, C. Zhao, Y. Lei, D. Liu, S. Zhang, Y. Ochiai, S. Liu, S. Wei, J. B.-H. Tok, Z. Bao, High-speed and large-scale intrinsically stretchable integrated circuits. *Nature* **627**, 313–320 (2024).

19. Q. Su, Q. Zou, Y. Li, Y. Z. Chen, S. Y. Teng, J. T. Kelleher, R. Nith, P. Cheng, N. Li, W. Liu, S. L. Dai, Y. D. Liu, A. Mazursky, J. Xu, L. H. Jin, P. Lopes, S. H. Wang, A stretchable and strain-unperturbed pressure sensor for motion interference-free tactile monitoring on skins. *Sci. Adv.* **7**, eabi4563 (2021).

20. J. Guo, J. Tuo, J. Sun, Z. Li, X. Guo, Y. Chen, R. Cai, J. Zhong, L. Xu, Stretchable Multimodal Photonic Sensor for Wearable Multiparameter Health Monitoring. *Adv. Mater.* **37**, e2412322 (2025).

21. H. J. Yang, J.-W. Lee, S. H. Seo, B. Jeong, B. Lee, W. J. Do, J. H. Kim, J. Y. Cho, A. Jo, H. J. Jeong, S. Y. Jeong, G.-H. Kim, G.-W. Lee, Y.-E. Shin, H. Ko, J. T. Han, J. H. Park, Fully stretchable self-charging power unit with micro-supercapacitor and triboelectric nanogenerator based on oxidized single-walled carbon nanotube/polymer electrodes. *Nano Energy* **86**, 106083 (2021).

22. S. Wang, S. Xiao, H. Cai, W. Sun, T. Wu, Y. Wang, J. He, S. Yang, Z.-D. Huang, W.-Y. Lai, Elastic Polymer Electrolytes Integrated with In Situ Polymerization-Transferred Electrodes toward Stretchable Batteries. *ACS Energy Lett.* **9**, 3672–3682 (2024).

23. N. Matsuhisa, S. Niu, S. J. K. O'Neill, J. Kang, Y. Ochiai, T. Katsumata, H.-C. Wu, M. Ashizawa, G.-J. N. Wang, D. Zhong, X. Wang, X. Gong, R. Ning, H. Gong, I. You, Y. Zheng, Z. Zhang, J. B.-H. Tok, X. Chen, Z. Bao, High-frequency and intrinsically stretchable polymer diodes. *Nature* **600**, 246–252 (2021).

24. S. Jang, H. Shim, C. J. Yu, Fully rubbery Schottky diode and integrated devices. *Sci. Adv.* **8**, eade4284 (2022).

25. D. Shin, S. Kim, H. Park, Y. Kim, M. Na, D. Kim, K. Sim, Soft Schottky diodes for skin-interfaced electronics enabled by entirely soft components. *Soft Sci.* **4**, 22 (2024).

26. H.-R. Lee, J. Woo, S. H. Han, S.-M. Lim, S. Lim, Y.-W. Kang, W. J. Song, J.-M. Park, T. D. Chung, Y.-C. Joo, J.-Y. Sun, A Stretchable Ionic Diode from Copolyelectrolyte Hydrogels with Methacrylated Polysaccharides. *Adv. Funct. Mater.* **29**, 1806909 (2019).

27. H. J. Kim, B. Chen, Z. Suo, R. C. Hayward, Ionoelastomer junctions between polymer networks of fixed anions and cations. *Science* **367**, 773–776 (2020).

28. P. Xu, X. Wu, Z. Zhang, P. Pan, X. Liu, Enhancing the rectification effect of hydrogel-based stretchable ionic diodes through incorporating cations with high valence. *Microsyst. Nanoeng.* **11**, 139 (2025).

29. Y. Kim, G. Kim, B. Ding, D. Jeong, I. Lee, S. Park, B. J. Kim, I. McCulloch, M. Heeney, M.-H. Yoon, High-Current-Density Organic Electrochemical Diodes Enabled by Asymmetric Active Layer Design. *Adv. Mater.* **34**, e2107355 (2022).

30. G. D. Spyropoulos, J. N. Gelinas, D. Khodagholy, Internal ion-gated organic electrochemical transistor: A building block for integrated bioelectronics. *Sci. Adv.* **5**, eaau7378 (2019).

31. R. Granelli, Z. M. Kovács-Vajna, F. Torricelli, Additive Manufacturing of Organic Electrochemical Transistors: Methods, Device Architectures, and Emerging Applications. *Small* **21**, e2410499 (2025).

32. S. Liu, Y. Rao, H. Jang, P. Tan, N. Lu, Strategies for body-conformable electronics. *Matter* **5**, 1104–1136 (2022).

33. X. Xie, Q. Wang, F. Mushtaq, K. Ao, H. Zhao, W. A. Daoud, Adhesion Reinforcement of Electrode–Electrolyte Interface in Flexible Electrochemical Energy Storage Devices. *Nano-Micro Lett.* **18**, 255 (2026).

34. Z. Zhu, Y. Xia, J. Li, C. Jiang, H. Jiang, Rate dependent shear debonding between a highly stretchable elastomer and a rigid substrate: Delayed debonding and pre-stretch effect. *Eng. Fract. Mech.* **222**, 106743 (2019).

35. Z. Zhu, Z. Yang, F. Yang, C. Yao, H. Jiang, Enhancing interfacial shear debonding resistance by mechanical mismatch. *Int. J. Mech. Sci.* **260**, 108656 (2023).

36. K. H. Lee, M. S. Kang, S. P. Zhang, Y. Y. Gu, T. P. Lodge, C. D. Frisbie, “Cut and Stick” Rubbery Ion Gels as High Capacitance Gate Dielectrics. *Adv. Mater.* **24**, 4457–4462 (2012).

37. K. Sim, Z. Rao, H.-J. Kim, A. Thukral, H. Shim, C. Yu, Fully rubbery integrated electronics from high effective mobility intrinsically stretchable semiconductors. *Sci. Adv.* **5**, eaav5749 (2019).

38. H. Park, S. Kim, J. Lee, K. Kim, H. Na, Y. Kim, D. Kim, D. Shin, B. Kim, K. Sim, Facile strategy for uniform gold coating on silver nanowires embedded PDMS for soft electronics. *npj Flex. Electron.* **8**, 63 (2024).

39. Y. Wang, Z. Wang, Z. Su, S. Cai, Stretchable and transparent ionic diode and logic gates. *Extreme Mech. Lett.* **28**, 81–86 (2019).

40. S. W. Lee, J. Jang, Y. Kim, S. Lee, K. Lee, H. Han, H. Lee, J. W. Oh, H. Kim, T. Kim, M. D. Dickey, C. Park, Intrinsically stretchable ionoelastomer junction logic gate synchronously deformable with liquid metal. *Appl. Phys. Rev.* **9**, 041404 (2022).

41. R. Huo, G. Bao, Z. He, X. Li, Z. Ma, Z. Yang, R. Moakhar, S. Jiang, C. Chung-Tze-Cheong, A. Nottegar, J. Li, Tough Transient Ionic Junctions Printed with Ionic Microgels. *Adv. Funct. Mater.* **33**, 2213677 (2023).

42. X. Wu, P. Xu, Z. Zhang, Q. Yang, X. Huang, P. Pan, X. Liu, Ultrasoft Iontronics: Stretchable Diodes Enabled by Ionically Conductive Bottlebrush Elastomers. *Adv. Funct. Mater.* **35**, 2503859 (2025).

43. J. Semple, S. Rossbauer, C. H. Burgess, K. Zhao, L. K. Jagadamma, A. Amassian, M. A. McLachlan, T. D. Anthopoulos, Radio Frequency Coplanar ZnO Schottky Nanodiodes Processed from Solution on Plastic Substrates. *Small* **12**, 1993–2000 (2016).

44. C. Yan, L. Xiang, Y. Xiao, X. Zhang, Z. Jiang, B. Zhang, C. Li, S. Di, F. Zhang, Lateral intercalation-assisted ionic transport towards high-performance organic electrochemical transistor. *Nat. Commun.* **15**, 10118 (2024).

45. D. Ohayon, V. Druet, S. Inal, A guide for the characterization of organic electrochemical transistors and channel materials. *Chem. Soc. Rev.* **52**, 1001–1023 (2023).

46. S. D. Ha, S. Ramanathan, Adaptive oxide electronics: A review. *J. Appl. Phys.* **110**, 071101 (2011).

47. J. Hwang, Y. Kim, H. Yang, J. H. Oh, Fabrication of hierarchically porous structured PDMS composites and their application as a flexible capacitive pressure sensor. *Comp. Part B Eng.* **211**, 108607 (2021).

48. J. Y. Oh, M. Shin, T. I. Lee, W. S. Jang, Y. Min, J. M. Myoung, H. K. Baik, U. Jeong, Self-Seeded Growth of Poly(3-hexylthiophene) (P3HT) Nanofibrils by a Cycle of Cooling and Heating in Solutions. *Macromolecules* **45**, 7504–7513 (2012).

49. H. L. Wang, T. Chen, B. Zhang, G. Wang, X. Yang, K. Wu, Y. Wang, A Dual-Responsive Artificial Skin for Tactile and Touchless Interfaces. *Small* **19**, e2206830 (2023).

50. S. Wang, X. Chen, C. Zhao, Y. Kong, B. Lin, Y. Wu, Z. Bi, Z. Xuan, T. Li, Y. Li, W. Zhang, E. Ma, Z. Wang, W. Ma, An organic electrochemical transistor for multi-modal sensing, memory and processing. *Nat. Electron.* **6**, 281–291 (2023).

51. D. K. Owens, R. C. Wendt, Estimation of the surface free energy of polymers. *J. Appl. Polym. Sci.* **13**, 1741–1747 (1969).

52. A. V. Prydatko, L. A. Belyaeva, L. Jiang, L. M. C. Lima, G. F. Schneider, Contact angle measurement of free-standing square-millimeter single-layer graphene. *Nat. Commun.* **9**, 4185 (2018).

53. A. Kozbial, Z. Li, C. Conaway, R. McGinley, S. Dhingra, V. Vahdat, F. Zhou, B. D'Urso, H. Liu, L. Li, Study on the Surface Energy of Graphene by Contact Angle Measurements. *Langmuir* **30**, 8598–8606 (2014).

54. J. O. Guardado, A. Salleo, Structural Effects of Gating Poly(3-hexylthiophene) through an Ionic Liquid. *Adv. Funct. Mater.* **27**, 1701791 (2017).

55. Y. Na, F. S. Kim, Nanodroplet-Embedded Semiconducting Polymer Layers for Electrochemically Stable and High-Conductance Organic Electrolyte-Gated Transistors. *Chem. Mater.* **31**, 4759–4768 (2019).

56. J. J. Samuel, A. Garudapalli, C. Gangadharappa, S. R. Mahapatra, S. Patil, N. P. B. Aetukuri, Charge polarity-dependent ion-insertion asymmetry during electrochemical doping of an ambipolar π-conjugated polymer. *Nat. Commun.* **13**, 7788 (2022).

57. Z. Wang, C. Priest, Impact of Nanoscale Surface Heterogeneity on Precursor Film Growth and Macroscopic Spreading of [Rmim][NTf_2] Ionic Liquids on Mica. *Langmuir* **29**, 11344–11353 (2013).

58. K. H. Lee, S. Zhang, Y. Gu, T. P. Lodge, C. D. Frisbie, Transfer Printing of Thermoreversible Ion Gels for Flexible Electronics. *ACS Appl. Mater. Interfaces* **5**, 9522–9527 (2013).
